# Half-Sandwich Ruthenium and Osmium Complexes with Hydrazinocurcuminoid-like Ligands

**DOI:** 10.1021/acs.organomet.5c00082

**Published:** 2025-05-16

**Authors:** Noemi Pagliaricci, Riccardo Pettinari, Fabio Marchetti, Sara Pagliaricci, Massimiliano Cuccioloni, Anna Maria Eleuteri, Agustín Galindo, Farzaneh Fadaei-Tirani, Kseniya Glinkina, Paul J. Dyson

**Affiliations:** † School of Pharmacy, 18959 University of Camerino, via Madonna delle Carceri, 62032 Camerino, MC, Italy; ‡ School of Science and Technology, and, 18959 University of Camerino, via Madonna delle Carceri, 62032 Camerino, MC, Italy; § School of Biosciences and Veterinary Medicine, 18959 University of Camerino, via Madonna delle Carceri, 62032 Camerino, MC, Italy; ∥ Departamento de Química Inorgánica, Facultad de Química, 16778 Universidad de Sevilla, Aptdo 1203, 41071 Sevilla, Spain; ⊥ Institut des Sciences et Ingénierie Chimiques, 27218 École Polytechnique Fédérale de Lausanne (EPFL), 1015 Lausanne, Switzerland

## Abstract

Ruthenium and osmium half-sandwich complexes with hydrazinocurcuminoid ligands, 4,4′-((1*E*,1′*E*)-(1-(pyridin-2-yl)-1*H*-pyrazole-3,5-diyl)­bis­(ethene-2,1-diyl))­bis­(2-methoxyphenol) (HZPcurc) and 4,4′-((1*E*,1′*E*)-(1-(pyridin-2-yl)-1*H*-pyrazole-3,5-diyl)­bis­(ethene-2,1-diyl))­diphenol (HZPbdcurc), have been synthesized and characterized using NMR spectroscopy and mass spectrometry. Two of the complexes were also characterized in the solid state using X-ray diffraction analysis, confirming the pseudo-octahedral “three-legged piano-stool” geometry. Density functional theory (DFT) studies are performed on the ligands to evaluate their coordinating capabilities and on the resulting ruthenium and osmium complexes. The complexes are highly soluble in water and stable under physiological conditions. Their cytotoxicity against MCF-7 human breast adenocarcinoma and A2780 human ovarian carcinoma cells, both normal and cisplatin-resistant, was investigated, and good activity and selectivity with respect to nontumorigenic cells (HEK293T) were observed.

## Graphical abstract

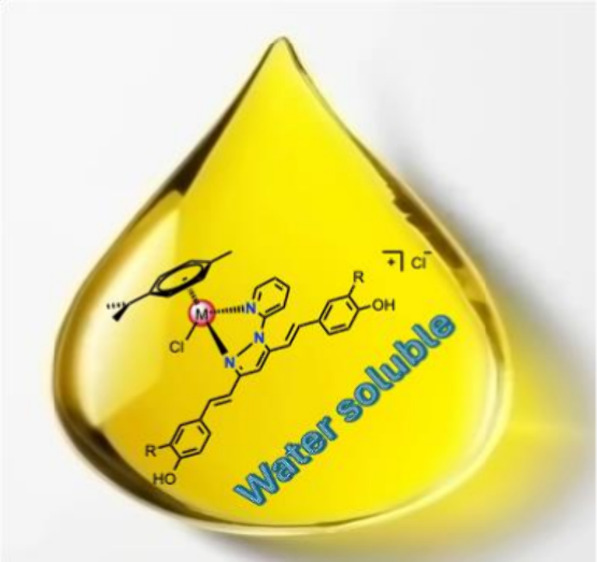

## Introduction

Curcumin and bisdemethoxycurcumin are natural products found in the rhizome of Curcuma longa and have shown therapeutic potential in chemoprevention and cancer treatment.^1–3^ Unfortunately, curcuminoid applications are limited by their low water solubility, which results in poor oral bioavailability and low chemical stability.^4^ In particular, keto–enolic tautomerism in solution was found to be responsible for the rapid degradation of curcumin, and the presence of the methylene group and β-diketone moiety contributes to its instability under physiological conditions. Notably, the α,β-unsaturated ketone, as a Michael acceptor, can form adducts with the −SH groups and generate reactive oxygen species,^5^ and modification at this site may lead to superior anticancer activity. Extending the conjugated system of azo compounds increases the chemical and thermal stability and solubility.^6^ In the past years, five-membered heterocycles have been introduced in the diketone site of curcumin, such as pyrazole, isoxazole, triazole, etc.^7,8^ Some five-membered heterocyclic curcumin derivatives show higher water solubility and more significant biological activity compared to curcumin.^9^ In particular, replacing the central diketone moiety with pyrazole groups to form hydrazinocurcumin (Figure 1), results in higher stability and fascinating biological activity.^10,11^ Unfortunately, hydrazinocurcumin does not have the characteristics to be used as a ligand for the preparation of metal complexes. To overcome this limitation, we introduced a pyridine group capable of forming neutral N,N-donor ligands, which can be coordinated to ruthenium and osmium half-sandwich acceptors.

**Figure 1:**
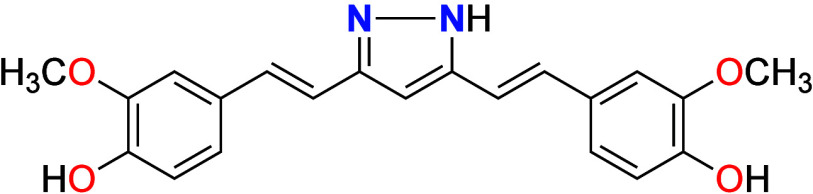
Hydrazinocurcumin.

In recent years, ruthenium­(II) and osmium­(II) arene complexes have emerged as a promising class of chemotherapeutic agents.^12–19^ These complexes exhibit intriguing chemical properties that contribute to their distinct mechanisms of action compared with platinum anticancer agents. Here, we describe the heterocyclization of curcumin and bisdemethoxycurcumin using 2-hydrazinopyridine. The resulting pyrazole-analogous curcuminoid ligands were then used to synthesize Ru­(II) and Os­(II) half-sandwich complexes. They have been fully characterized, and their cytotoxic effect against human cancer and noncancerous cell lines has been evaluated.

## Results and Discussion

Following a similar procedure used to prepare HZPcurc from curcumin,^20^ HZPbdcurc was synthesized from bisdemethoxycurcumin. In brief, the corresponding curcuminoid was treated with 2-hydrazinopyridine in n-butanol and heated to reflux for 8 h in the presence of glacial acetic acid to facilitate the condensation step (Scheme 1). The product was isolated through chromatographic purification, and elemental analysis and ESI-MS spectrometry confirmed the expected structure of the ligand HZPbdcurc. Additionally, the IR spectra show the disappearance of the typical absorption band at 1640 cm^–1^, characteristic of the C=O double bond.^21^ Furthermore, a strong absorption band was found at 1548 cm^–1^ relative to C=N stretching.^22^

**Scheme 1:**
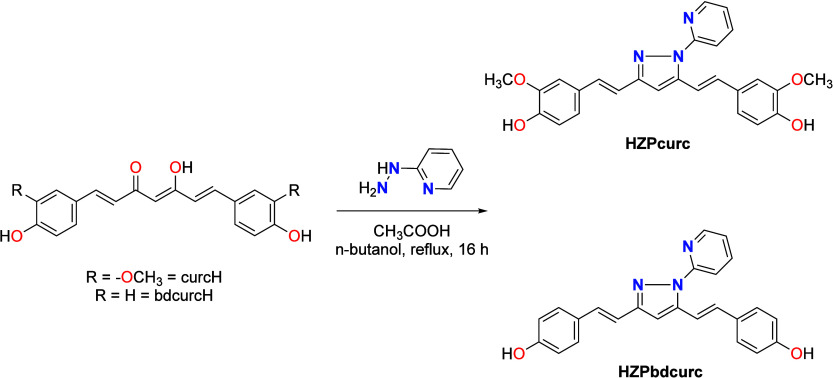
Synthesis of HZPcurc and HZPbdcurc

The ^1^H and ^13^C NMR spectra of HZPbdcurc, as well as complexes **[1]­Cl**-**[4]­Cl** were assigned based on the ^1^H–^1^H, one-bond and long-range ^1^H–^13^C couplings derived from {^1^H–^1^H}-COSY, {^1^H–^13^C}/{^1^H–^15^N}-HSQC, and {^1^H–^13^C}/{^1^H–^15^N}-HMBC experiments (Figures S1–S20, Supporting Information for further details). Complexes **[1]­Cl**-**[4]­Cl** were obtained by reacting HZPcurc or HZPbdcurc with the organometallic dimer [M­(*p*-cym)­Cl_2_]_2_ (M = Ru or Os) in CH_3_CN at room temperature for 4 h (Scheme 2), and were isolated by evaporation and recrystallization in dichloromethane/hexane solution. HZPcurc and HZPbdcurc act as neutral bidentate *N*,*N*-donor ligands to the metal center, affording five-membered metallocycles. They are air-stable and soluble in polar solvents and are highly soluble in water. Complexes **[1]­Cl**-**[4]­Cl** exist as 1:1 electrolytes in water, consistent with their ionic formulation in the solid state, with chloride acting as a counterion in the outer coordination sphere. See below the structural characterization of **[1]­Cl** and **[3]­Cl**.^23^ It is interesting to note that the far-IR spectra of complexes **[1]­Cl** and **[2]­Cl** show new absorption at 440 cm^–1^, while **[3]­Cl** and **[4]­Cl** show absorption at 461 cm^–1^, which may be assigned to ν­(M–N) stretching modes. Furthermore, a strong absorption in the range of 281–292 cm^–1^ may be assigned to ν­(M–Cl).^24^ Electrospray ionization (ESI-mass spectrometry) mass spectra of **[1]­Cl**-**[4]­Cl** obtained in the positive ion mode, recorded in CH_3_CN, shows the expected isotopic patterns and *m*/*z* values correspond to [M­(*p-*cym)­(HZPcurc/HZPbdcurc)­Cl]^+^ **[1]** ^**+**^-**[4]** ^**+**^ (Figures S21–S24).

**Scheme 2:**
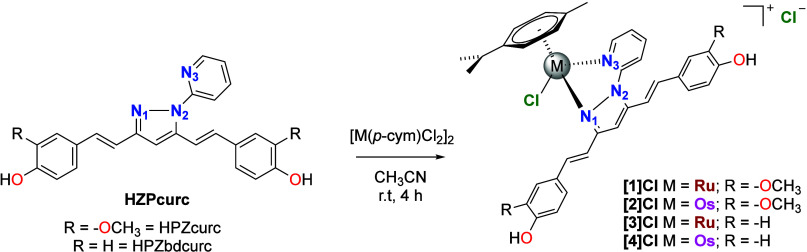
–Synthesis of Compounds **[1]­Cl**-**[4]­Cl**

The NMR spectra of HZPcurc and HZPbdcurc exhibit two distinct sets of signals resulting from their asymmetry induced by the presence of pyridine in the N2 position (Scheme 2). Upon coordination, a shift of the ligand signals toward lower fields was observed, for example, the H12 resonance of the pyridine ring moves from 8.58 ppm to values in the range 9.29–9.38 ppm in complexes **[1]­Cl**-**[4]­Cl** (Figures S8, S11, S14, S18, S20, and S23). ^15^N NMR chemical shifts were assigned based on the {^1^H–^15^N}-HMBC of the free ligands with respect to those of complexes **[1]­Cl**-**[4]­Cl** (Table 1).

**Table 1:** ^15^N Chemical Shifts (ppm) in **HZPcurc**, **HZPbdcurc**, and **[1]­Cl-[4]­Cl** Obtained from {^1^H- ^15^N} HMBC NMR Experiments as Numbered in Scheme 2

compound	N_1_–H pyrazole	N_2_–H pyrazole	N_3_–H pyridine
**HZPcurc**	299.0	213.5	288.9
HZPbdcurc	299.6	214.8	n.o.
**[1]Cl**	218.2	211.0	213.8
**[2]Cl**	n.o.	n.o.	n.o.
**[3]Cl**	217.5	210.7	214.2
**[4]Cl**	212.2	201.5	n.o.

n.o.: not observed.

### X-ray Structural Characterization

Complexes **[1]­Cl** and **[3]­Cl** were structurally characterized by single-crystal X-ray crystallography. Their cations, **[1]** ^+^ and **[3]** ^+^, exhibit the expected three-legged piano-stool geometry around the ruthenium­(II) center (Figure 2). The Ru–Cl bond length is 2.397 and 2.396 Å in **[1]** ^+^ and **[3]** ^+^, respectively, and the Ru–N_py_ distance is slightly longer than the Ru–N_pyrazolyl_ distance (e.g., Ru1–N1 2.070(8) versus Ru1–N3 2.104(7) Å for complex **[3]** ^+^). The average Ru–C_arene_ bond length is ∼2.20 Å (Ru–C_centroid_ ∼ 1.69 Å), which is typical for these types of complexes. Additional structural parameters are summarized in Table S1. In both complexes, the HPZcurc and HPZbdcurc ligands are essentially planar, with minor deviations from planarity observed in one of the phenyl rings (angles between planes of 16.3° and 24.9°, respectively).

**Figure 2:**
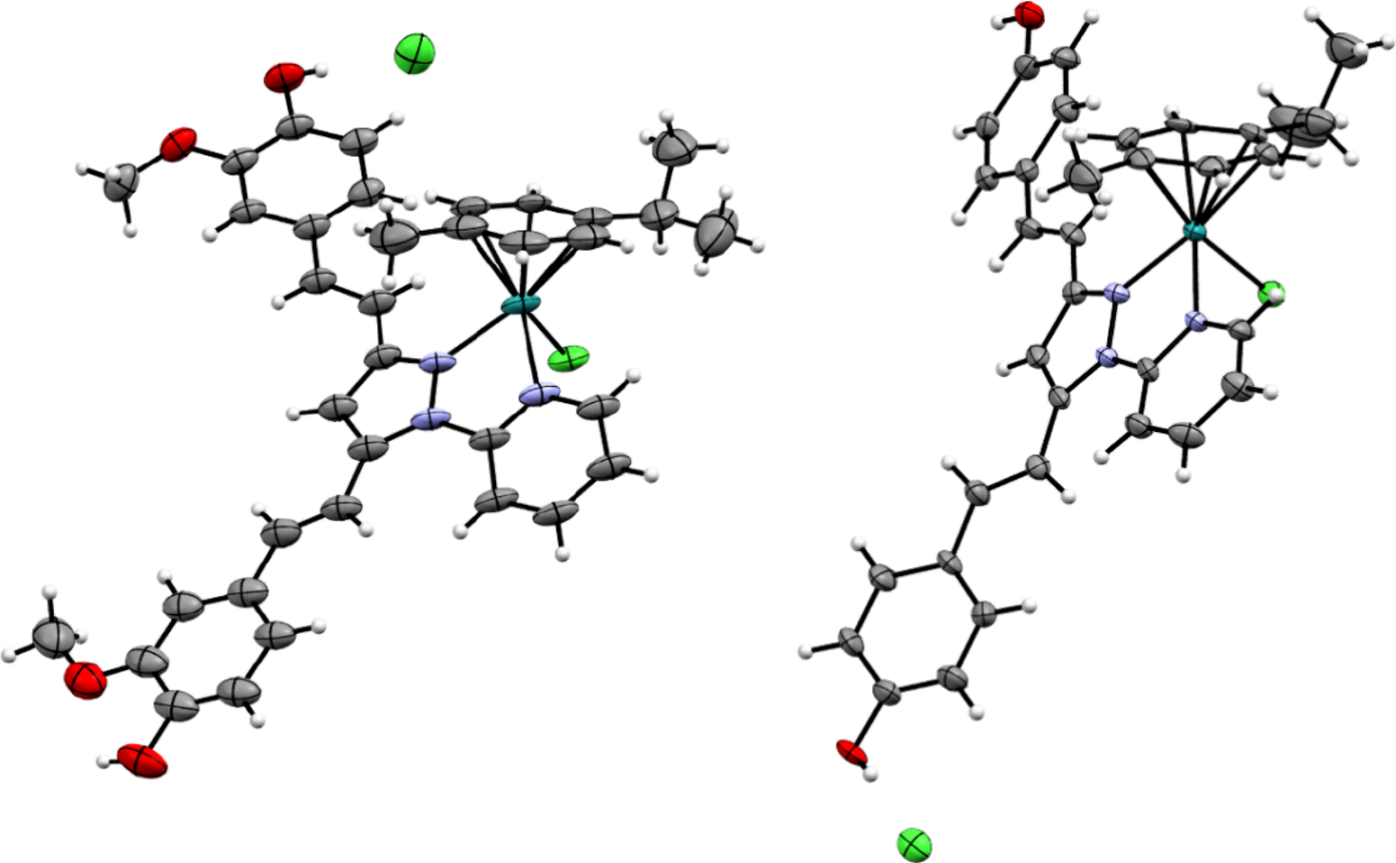
Structures of complexes **[1]­Cl** (left) and **[3]­Cl** (right). Color codes: C, gray; H, white; O, red; N, light violet; Cl, green; Ru, turquoise.

These ligands adopt a nearly planar five-membered metallacycle upon coordination to the ruthenium center. In complex **[1]­Cl**, the chloride anion forms a nonclassical O–H^···^Cl hydrogen bond with the O–H group of the HPZcurc ligand, characterized by an O^···^Cl distance of 3.061(7) Å and an O–H^···^Cl angle of 177.9°. The three-dimensional packing of complex **[1]­Cl** is governed by parallel intercalation of HPZcurc ligands through π-stacking interactions (Figure S25). For **[3]­Cl**, the asymmetric unit contains two independent complexes with different orientations of the HPZbdcurc ligand relative to the Ru­(*p-*cym)Cl moiety (Figure S26). However, no significant differences are observed in their structural parameters as they are enantiomerically related. In **[3]­Cl**, a bifurcated O–H^···^Cl^···^H–O hydrogen bond exists, where the chloride anion interacts with one O–H group of the HPZbdcurc ligand and another O–H group of a symmetry related ligand (O^···^Cl distances of 3.027 and 3.038 Å and an O^···^Cl^···^O angle of 107.9°, Figure S27). The crystal packing of **[3]­Cl** is defined by these bifurcated hydrogen bonds and the intercalation of HPZbdcurc ligands, which display interplane distances of 3.42 Å (see Figures S27 and S28).

### Theoretical Studies

Initially, the ligands HZPcurc and HZPbdcurc were analyzed using density functional theory (DFT) to assess their coordinating capabilities by examining their frontier molecular orbitals (MOs). Subsequently, the cations of **[1]­Cl**-**[4]­Cl** were also investigated to gain insight into their electronic structures. Geometry optimizations were performed without symmetry restrictions, starting from the experimental X-ray coordinates of complexes **[1]­Cl** and **[3]­Cl**. The optimized structures of the HZPcurc and HZPbdcurc ligands are shown in Figure S29, with selected structural parameters summarized in Table S3. Both ligands are essentially planar, although slight deviations from planarity are observed. Specifically, for HZPbdcurc, the pyridine ring and one phenyl ring deviate by 30.8° and 31.2°, respectively. These deviations arise due to an intramolecular hydrogen bond formed between the pyridine nitrogen atom and a C–H bond of the olefinic moiety (C–H^···^N distance of 2.161 Å, data for HZPcurc). Within the pyrazole rings of both ligands, bond distances indicate a degree of delocalization consistent with aromaticity (Table S3). Experimental C=N stretching vibrations at 1511 (for HZPcurc) and 1548 cm^–1^ (for HZPbdcurc), agree well with the calculated IR spectra, where absorptions at 1507 and 1508 cm^–1^, respectively, are assigned to the ν­(C=N) stretching with contributions from ν­(C=C) of the pyrazolyl ring. Analysis of the MOs reveals that the nitrogen σ lone pairs are primarily located in HOMO-7 and HOMO-8 for HZPcurc and in HOMO-6 and HOMO-8 for HZPbdcurc (Figure S30). These orbitals are key contributors to the σ-coordination of the ligands to the ruthenium or osmium centers once the *cisoid* conformation is achieved. The optimized structures of complexes **[1]­Cl**-**[4]­Cl** are depicted in Figure S31, with selected structural parameters provided in Table S4. As observed in previous DFT studies, the selected combination of method and basis sets yields a reliable structural description of ruthenium complexes **[1]­Cl** and **[3]­Cl**.^25,26^ A good correlation between experimental and calculated structural parameters is evident (Table S4), except for the Ru–C_arene_ bonds, which are slightly overestimated.^27,28^ The optimized structures of osmium complexes **[2]­Cl** and **[4]­Cl** closely resemble those of their ruthenium analogues (Table S4). In all complexes (**[1]­Cl**-**[4]­Cl**), the five-membered metallacycles, as well as the pyridine and pyrazolyl rings, remain essentially planar. However, in contrast to experimental observations for **[1]­Cl** and **[3]­Cl**, the phenyl rings in the optimized structures deviate from planarity. This discrepancy can be attributed to the crystal packing effect previously discussed in which planarity facilitates effective π-stacking interactions with neighboring molecules. The electronic structure of complex **[1]­Cl**, taken as a representative example, was further analyzed in terms of its MOs (Figure S32). HOMO and HOMO–1 are primarily associated with the olefinic C=C bond and its adjacent phenyl ring. HOMO–2 through HOMO–6 consist of contributions from the ruthenium *d* orbitals with some antibonding interactions with the chloro ligand. Finally, HOMO–7 reflects the π character of the pyrazolyl and pyridine rings within the metallacycle.

### Cytotoxicity Studies

The stability of **[1]­Cl**-**[4]­Cl** was evaluated in aqueous phosphate-buffered solution (PBS, pH 7.4) using UV–visible spectroscopy. All complexes are stable over 72 h (a time selected to mimic the viability assay conditions) and exhibit a main transition in the 350–400 nm range, attributable to MLCT (metal–ligand charge transfer) from the filled d orbitals to the unoccupied π* ligand orbitals (nd^6^ → π*) (Figure S33).^29^ The cytotoxicity of the compounds was measured according to a standard MTT assay against human breast cancer (MCF7 and MCF7CR) and ovarian cancer (A2780 and A2780cis) cell lines, and also nontumorigenic MCF10A and human embryonic kidney HEK293T cells (Table 2).

**Table 2:** Cytotoxicity Calculated From the MTT Assay Data of HZPcurc and HZPbdcurc and **[1]­Cl**-**[4]­Cl** toward MCF7 Human Breast Adenocarcinoma, A2780 Human Ovarian Carcinoma, MCF7-CR and A2780cis Cisplatin-Resistant Cancerous Counterparts, and MCF10A and HEK293T Nontumorigenic Cells^a^

compound	MCF7^a^	MCF7CR	MCF10A	SI	A2780	A2780cis	HEK293T	SI
				MCF7				A2780
				MCF10A				HEK293T
**HZPcurc**	23.0 ± 6	17.7 ± 3.6	7.0 ± 4.5	0.30	5.3 ± 0.4	6.7 ± 0.6	16.4 ± 0.6	3.10
**HZPbdcurc**	35.1 ± 3	25.9 ± 11	9.7 ± 6.5	0.28	8.0 ± 1.2	11.1 ± 2.9	17.0 ± 0.9	2.13
**[1]Cl**	49.2 ± 5	41.0 ± 10	31.7 ± 10	0.64	4.6 ± 0.3	6.6 ± 0.9	25.0 ± 1	5.43
**[2]Cl**	>100	>100	25.6 ± 15	0.26	6.3 ± 0.5	12.1 ± 0.6	25.0 ± 8	3.97
**[3]Cl**	23.0 ± 6	31.9 ± 6	15.6 ± 8.9	0.68	2.0 ± 0.1	2.7 ± 0.4	19.0 ± 2.0	9.50
**[4]Cl**	>100	>100	25.8 ± 10	0.26	2.4 ± 0.4	5.4 ± 0.3	32.0 ± 2	13.33
**Cisplatin**	4.2 ± 2.3	49.1 ± 7	11.5 ± 4.0	2.74	0.25 ± 0.02	7.0 ± 1.0	2.8 ± 0.5	11.20

a IC_50_ values (in μM) represent the mean from three independent experiments ± standard deviation following exposure to the compounds for 72 h.

HZPcurc and HZPbdcurc exhibit comparable cytotoxicity in the micromolar range against MCF7 cells and the cisplatin-resistant counterpart MCF7CR, but are not selective as the ligands are more active against noncancerous MCF10A cells. In these cell lines, complexes **[1]­Cl**-**[4]­Cl** either showed equivalent cytotoxicity to the ligands or were less active. The osmium derivatives, **[2]­Cl** and **[4]­Cl**, are not cytotoxic below concentrations of 100 μM in MCF7 and MCF7CR cells, and were moderately cytotoxic toward nontumorigenic MCF10A cells, i.e., with IC_50_ values of 25.6 ± 14.9 and 25.8 ± 10.4 μM, respectively. All the compounds were considerably more cytotoxic toward the ovarian cancer cell lines, with the complexes exhibiting cancer cell selectivity, demonstrating the benefit of **[1]­Cl**-**[4]­Cl** over the ligands. Complex **[3]­Cl** has the most promising cytotoxicity profile, overcoming cisplatin resistance in A2780cis cells with an IC_50_ value of 2.7 ± 0.4 μM compared to 7.0 ± 1.0 μM for cisplatin, coupled with much lower activity toward both nontumorigenic cell lines, with values of 15.6 ± 8.9 and 19.0 ± 2.0 μM for MCF10A and HEK293T cells, respectively.

### DNA Binding Studies

The DNA binding ability of curcuminoid ligands and their complexes was evaluated by using a Surface plasmon resonance (SPR) biosensor. The monoexponential nature of binding kinetics for all the compounds is consistent with the presence of a single high-affinity site on DNA (Figure 3).

**Figure 3:**
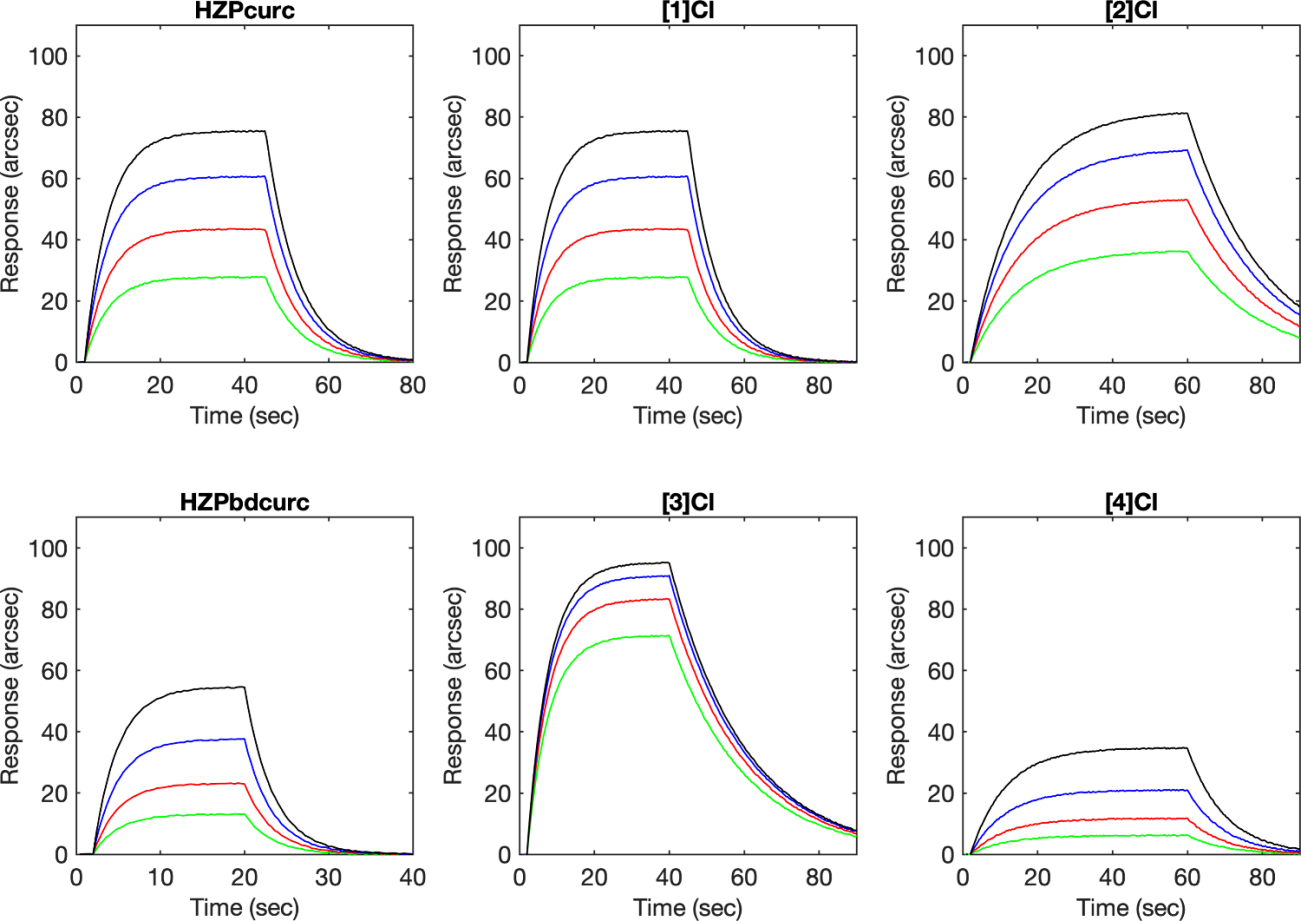
Representative superimposition of sensorgrams obtained upon the binding of soluble curcuminoid ligands and metal complexes to a surface-anchored dsDNA oligomer.

The minor groove of DNA was experimentally and computationally identified as the preferential target site for all compounds (see Table S5, and Figures S34 and S35). Reversible interactions were always observed for both ligands and complexes, with equilibrium dissociation constants in the range of 0.30–0.40 μM. The HZPcurc series of complexes generally form more stable interactions with DNA compared to their HZPbdcurc counterparts due to favorable contributions of association and dissociation phases (Table 3). Interestingly, the effects on kinetic and thermodynamic parameters were strongly dependent on the nature of the metal ion, with only the ruthenium complexes promoting kinetically and thermodynamically the interaction with DNA, whereas osmium had no effect or even decreased binding relative to the free ligand.

**Table 3:** Kinetic and Equilibrium Parameters for the Interaction between HZPcurc, HZPbdcurc and **[1]­Cl**-**[4]­Cl,** and Surface-blocked DNA Oligomer

compound	*k*_ass_ (M^–1^s^–1^)	*k*_diss_ (s^–1^)	*K*_D_ (μM)
**HZPcurc**	2994 ± 1142	0.013 ± 0.009	4.5 ± 2.7
HZPbdcurc	1808 ± 740	0.031 ± 0.004	17.0 ± 7.3
**[1]Cl**	32082 ± 773	0.009 ± 0.004	0.29 ± 0.14
**[2]Cl**	1156 ± 179	0.005 ± 0.002	4.2 ± 1.8
**[3]Cl**	10281 ± 5155	0.02 ± 0.01	2.2 ± 1.5
**[4]Cl**	266 ± 102	0.010 ± 0.002	39.2 ± 17.0

### Effect on Proteasome Activity

Proteasome inhibition by the compounds was evaluated with respect to three major activities of the 20S proteasome, namely, chymotrypsin-like, trypsin-like, and caspase-like activities. The ligands did not inhibit proteasome hydrolytic activities in the range of concentrations tested, and the complexes were largely ineffective. Only Ru and Os coordination of HZPcurc (**[1]­Cl** and **[2]­Cl)**, and Os coordination of HZPbdcurc (**[4]­Cl**) resulted in inhibition of the trypsin-like activity (Table 4), an established mechanism for certain anticancer drugs.^30^

**Table 4:** IC_50_ Values for HZPcurc, HZPbdcurc, and **[1]­Cl-[4]­Cl** against Proteasomal Activity

compound	ChT-L (μM)	T-L (μM)	PGPH (μM)
**HZPcurc**	>100	>100	>100
HZPbdcurc	>100	>100	>100
**[1]Cl**	>100	63 ± 7	>100
**[2]Cl**	84 ± 60	51 ± 13	>100
**[3]Cl**	>100	>100	>100
**[4]Cl**	98 ± 50	61 ± 44	>100

### p62 Expression

Since no significant correlation emerged among cytotoxicity, DNA binding affinity, and proteasome inhibition, the effects of the treatment on the expression levels of p62 were evaluated. p62 is a multifunctional binding protein that is involved in signaling pathways of many cellular activities, including autophagy, whose abnormal expression is associated with malignancies, breast cancer in particular.^31^ Upon treatment of MCF7 cells with HZPbdcurc and **[3]­Cl**, an increase in the levels of p62 was observed, suggesting inactivation of the autophagic flux (p62 levels correlate inversely with the autophagic activity).^32^ This effect was more evident for the free ligand, in agreement with its higher cytotoxicity to breast cancer cells compared with that of the corresponding ruthenium complex (Figure 4).

**Figure 4:**
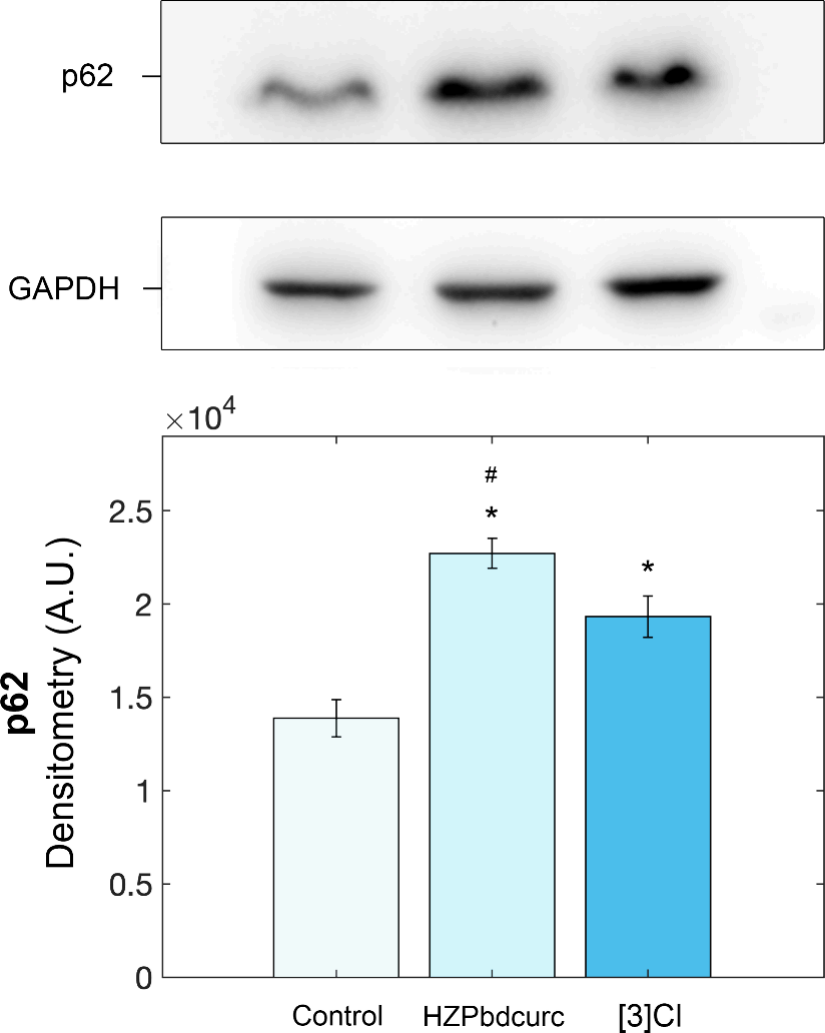
Representative autoradiographs (upper panel) and densitometric analyses (lower panel) of p62 levels in MCF7 cells treated with 10 μM of HZPbdcurc and **[3]­Cl** for 24 h (*p < 0.01 compared with the control; #*p* < 0.01 compared with **[3]­Cl**).

## Conclusions

A series of half-sandwich ruthenium­(II) and osmium­(II) complexes containing hydrazinocurcuminoid-like ligands were prepared. The innovative nature of these complexes is highlighted by the asymmetrical nature of their ligands, which differ from conventional curcuminoid O,O-donors. HZPcurc and HZPbdcurc bind ruthenium and osmium as *N*,*N*-donors in a neutral form, affording 5-membered chelating rings and ionic complexes confirmed by X-ray studies in the solid state. Notably, these complexes demonstrate good solubility and stability as 1:1 electrolytes in polar solvents such as water. Additionally, the ruthenium complex **[3]­Cl** exhibits favorable cytotoxicity toward A2780 and A2780cis cells, overcoming cisplatin resistance, while displaying considerably less activity toward the two nontumorigenic cell lines evaluated. All of the complexes interact with DNA and the 20S proteasome to varying degrees. However, the absence of a clear correlation with cellular toxicity suggests that alternative targets may be involved. The observed increase in p62 hub protein levels implies a mechanism of action that includes inactivation of autophagy, which, in turn, could lower the apoptotic threshold and increase the susceptibility of cells to cytotoxic agents.

## Experimental Section

### Materials and Methods

The dimer [(*p-*cymene)­RuCl_2_]_2_ was purchased from Aldrich, and [(*p-*cymene)­OsCl_2_]_2_ was synthesized using a literature method.^33^ Curcumin and bisdemethoxycurcumin were purchased from TCI Europe and were used as received. All reactions were carried out in the air. Melting points were recorded on an STMP3 Stuart scientific instrument and on a capillary apparatus. Samples for microanalysis were dried in vacuo to a constant weight (35 °C, ca. 0.1 Torr). Elemental analyses (C, H, N) were performed in-house with a Fisons Instruments 1108 CHNS-O Elemental Analyzer. Infrared spectra (IR) were recorded on a PerkinElmer Frontier FT-IR instrument. ^1^H, ^13^C, {^1^H–^1^H}-COSY, {^1^H–^13^C}-HSQC, {^1^H–^13^C}-HMBC and {^1^H–^15^N}-HMBC nuclear magnetic resonance (NMR) spectra were recorded on a 500 Bruker Ascend (500 MHz for ^1^H, 125 MHz for ^13^C) instrument operating at room temperature relative to TMS. Positive ion electrospray mass spectra were obtained on a Series 1100 MSI detector HP spectrometer, using acetonitrile as solvent. Solutions (3 mg/mL) for electrospray ionization mass spectrometry (ESI–MS) were prepared by using reagent-grade methanol. Masses and intensities were compared to those calculated using the IsoPro Isotopic Abundance Simulator, version 2.1.28. UV-stability studies were conducted on a Varian Cary spectrometer. Electrical conductivity measurements (ΛM, reported as Ω^–1^ cm^2^ mol^–1^) of aqueous solutions of the complexes were recorded using a Crison CDTM 522 conductimeter at room temperature.

### Synthesis of the Compounds

#### HZPcurc

HZPcurc was synthesized according to literature procedures.^20,34^ mp 91–93 °C. Anal. Calcd. for C_26_H_23_N_3_O_4_: C, 70.74; H, 5.25; N 9.52. Found: C, 70.64; H, 5.08; N 9.37. IR (cm^–1^): 3386 mbr ν­(O–H); 3059 w, 3009 w, 2960 w, 2935 w, 2837 w ν­(C–H aromatic); 1588 s, 1537 m, 1510 vs ν­(C=C, C=N), 1469 s, 1444 s, 1428 s, 1366 s. ^1^H NMR (DMSO-*d*_6_, 293 K): δ 3.83 (s, 3H, -OC*H*_*3*_), 3.86 (s, 3H, −OC*H*_*3*_), 6.80 (t, 2H, C(9–9)*H*, J^3^ = 8 Hz), 7.00 (d, 2H, C­(10-10′)*H,* J^3^ = 8 Hz), 7.06 (d, 1H, C(3)*H,* J^3^ = 16 Hz), 7.11 (s, 1H, C(6′)*H*), 7.17 (s, 1H, C(1)*H*), 7.19 (d, 1H, C(4′)*H,* J^3^ = 11 Hz), 7.23 (s, 1H, C(4)*H,* J^3^ = 11 Hz), 7.25 (s, 1H, C(6)*H*), 7.41 (t, 1H, C(13)*H,* J^3^ = 6 Hz), 7.79 (d, 1H, C(3′)*H,* J^3^ = 16 Hz), 7.87 (d, 1H, C(15)*H,* J^3^ = 8 Hz), 8.03 (t, 1H, C(14)*H,* J^3^ = 8 Hz), 8.58 (d, 1H, C(12)*H,* J^3^ = 6 Hz), 9.22 (sbr, 1H, −O*H*), 9.29 (sbr, 1H, −O*H*). ^13^C­{^1^H}-NMR (DMSO-*d*_6_, 293 K): δ 56.1 (−OC*H*_3_), 102.47 (C1), 110.2 (C6), 110.9 (C6′), 115.13 (C3′), 116.1, 116.3 (C­(9-9′)), 116.8 (C15), 117.5 (C3), 120.6, 120.9 (C­(10-10′)), 122.3 (C13), 128.6, 128.7 (C­(5-5′)), 132.3 (C4), 132.7 (C4′), 139.7 (C14), 144.1 (C2′), 147.5, 147.8 (C­(8-8′)], 148.3, 148.4 (C­(7-7′) and C12), 152.1 (C2) and 153.3 (C11). {^1^H, ^15^N} HMBC NMR (DMSO-*d*_6_, 293 K): δ_N_ 299.0 (N_1_-H), 213.5 (N_2_-H), 288.9 (N_3_-H).

#### HZPbdcurc

Bisdemethoxycurcumin (369 mg, 1.2 mmol) and 2-hydrazinopyridine (218 mg, 2 mmol) were dissolved in *n*-butanol (10 mL) together with a solution of glacial acetic acid (9 mL). The reaction was stirred under reflux for 16 h, and then the solvent was removed and the product isolated through a chromatographic separation in a solution of hexane: ethyl acetate (60:40) obtaining HZPcurc as orange powder (67% of yield) and is soluble in CH_3_OH, CH_3_CN, DMSO, CHCl_3_, CH_2_Cl_2_ and insoluble in H_2_O. mp 137–138 °C. Anal. Calcd. for C_24_H_19_N_3_O_2_: C, 75.57; H, 5.02; N 11.02. Found: C, 75.28; H, 4.96; N 11.14. IR (cm^–1^): 3200 sbr ν­(O–H), 3111 w, 3087 w, 3051 w ν­(C–H aromatic), 1640 s, 1604 w, 1590 w, 1548 m ν­(C=C, C=N), 1524 m, 1505 s, 1472 m, 1441 s, 1388 s, 1379 s. ^1^H NMR (DMSO-*d*_6_, 293 K): δ 6.80 (dd, 4H, C­(6-6′)*H* and C(10–10′)*H*), 6.98 (d, 1H, C­(3/3′)*H,* J^3^ = 16 Hz), 7.18 (s, 1H, C(1)*H*), 7.19 (d, 1H, C­(4/4′)*H,* J^3^ = 12 Hz), 7.23 (d, 1H, C­(4/4′)*H,* J^3^ = 12 Hz), 7.40 (m, 1H, C(13)*H*), 7.39 (d, 2H, C­(7-7′)*H,* J^3^ = 9 Hz*)*, 7.46 (d, 2H, C­(9-9′)*H,* J^3^ = 9 Hz), 7.73 (d, 1H, C­(3/3′)*H,* J^3^ = 16 Hz), 7.86 (d, 1H, C(15)*H,* J^3^ = 8 Hz), 8.02 (t, 1H, C(14)*H,* J^3^ = 8 Hz), 8.57 (d, 1H, C(12)*H,* J^3^ = 6 Hz), 9.67 (sbr, 2H, −O*H*). ^13^C­{^1^H}-NMR (DMSO-*d*_6_, 293 K): δ 102.4 (C1), 114.8 (C3/3′), 116.1, 116.2 (C­(6-6′) and C­(10-10′)), 117.0, 117.2 (C3/3′ and C(15)), 122.3 (C(13)), 128.1 (C4-4′), 128.5, 128.6 (C­(7-7′) and C­(9-9′)), 132.0, 132.5 (C­(5-5′)), 139.7 (C14), 144.1 (C(2′)), 148.3 (C12), 152.1 (C2), 153.4 (C11), 158.1, 158.3 (C­(8-8′)). {^1^H, ^15^N} HMBC NMR (DMSO-*d*_6_, 293 K): δ_N_ 299.6 (N_1_–H), 214.8 (N_2_–H), not observed (N_3_–H).

##### [Ru­(*p-*cym)­(HZPcurc)­Cl]Cl ([1]­Cl)

HZPcurc (196 mg, 0.4 mmol) and the dimer [Ru­(*p-*cymene)­Cl_2_]_2_ (122 mg, 0.2 mmol) were dissolved in CH_3_CN (5 mL). After stirring at room temperature for 24 h, the solvent was partly removed under reduced pressure, and **[1]­Cl** was precipitated from the solution using Et_2_O. The orange precipitate (172 mg, yield 58%) is soluble in polar solvents, including H_2_O. Anal. Calcd. for C_36_H_37_Cl_2_N_3_O_4_Ru: C, 57.83; H, 4.99; N 5.62. Found: C, 57.75; H, 4.90; N, 5.37. mp 157–159 °C. IR (cm^–1^): 3064 sbr ν­(O–H), 3004 mbr, 2960 mbr, 2928 mbr ν­(C–H aromatic), 1590 s, 1535 m, 1515 s ν­(C=C, C=N), 1470 vs, 1384 s, 1277 vs ν­(N–N), 440 m ν­(Ru–N), 283 s ν­(Ru–Cl). ^1^H NMR (DMSO-*d*_6_, 293 K): δ 0.97 (dd, 6H, CH­(C*H*_3_)_2_ of *p-*cym), 2.30 (s, 3H, CH_3_ of *p-*cym), 2.52 (m, 1H, C*H*(CH_3_)_2_ of *p-*cym), 3.87 (s, 3H, -OC*H*_3_), 3.93 (s, 3H, −OC*H*_3_), 6.08 d, 6.10 d, 6.23 d, 6.31 d (4H, AA′BB′ system, CH_3_–C_6_ *H*_4_–CH­(CH_3_)_2_ of *p-*cym,^3^J = 6 Hz), 6.89 (d, 1H, C­(9/9′)*H*, J^3^ = 8 Hz), 6.97 (d, 1H, C­(9/9′)*H*, J^3^ = 8 Hz), 7.04 (d, 1H, C(3′)*H*, J^3^ = 16 Hz), 7.17 (d, C(10)*H*, J^3^ = 8 Hz), 7.26 (d, 1H, C(10′)*H*, J^3^ = 8 Hz), 7.30 (s, 1H, C(6′)*H*), 7.37 (d, 1H, C(4)*H*, J^3^ = 16 Hz), 7.40 (s, 1H, C(6)*H*), 7.47 (d, 1H, C(3)*H*, J^3^ = 16 Hz), 7.61 (s, 1H, C(1)*H*), 7.63 (t, 1H, C(13)*H*, J^3^ = 6 Hz), 7.69 (d, 1H, C(4′)*H*, J^3^ = 16 Hz), 8.00 (d, 1H, C(15)*H*, J^3^ = 8 Hz), 8.28 (t, 1H, C(14)*H*, J^3^ = 8 Hz), 9.38 (d, 1H, C(12)*H*, J^3^ = 6 Hz), 9.64 (s, 1H, -O*H*), 9.70 (s, 1H, −O*H*). ^13^C­{^1^H}-NMR (DMSO-*d*_6_, 293 K): δ 19.2 (−*C*H_3_ of *p-*cym), 22.0, 22.4 (−CH­(*C*H_3_)_2_ of *p-*cym), 30.9 (−*C*H­(CH_3_)_2_ of *p-*cym), 56.2, 56.4 (-O*C*H_3_ of the ligand), 81.6, 83.3 (C­(a-a′)), 85.9, 87.5 (C­(b-b′)), 103.7 (Ci′), 106.4 (Ci), 107.6 (C1), 110.8 (C3), 111.7, 111.8 (C­(6-6′)), 114.0 (C3′), 114.1 (C15), 116.2 (C9), 116.5 (C9′), 122.1 (C10′), 122.8 (C10), 123.8 (C13), 127.2, 127.3 (C­(5-5′)), 139.4 (C4), 139.7 (C4′), 142.8 (C14), 147.2 (C2′), 148.5, 149.3 (C­(7-7′)), 148.6 (C11), 155.7 (C12), 157.4 (C2). {^1^H, ^15^N} HMBC NMR (DMSO-*d*_6_, 293 K): δ_N_ 218.2 (N_1_-H), 211.0 (N_2_-H), 213.8 (N_3_-H). ESI-MS (+) CH_3_CN (*m*/*z* [relative intensity, %]): 712 [100] [**1**]^+^. Λ_m_ (*T* = 298 K, H_2_O, 10^–3^ mol L^–1^) 121 S cm^2^ mol^–1^.

##### [Os­(*p-*cym)­(HZPcurc)­Cl]Cl ([2]­Cl)

HZPcurc (176 mg, 0.4 mmol) and the dimer [Os­(*p-*cymene)­Cl_2_]_2_ (120 mg, 0.2 mmol) were dissolved in CH_3_CN (5 mL). After 4 h of stirring at room temperature, the solvent was partly removed at reduced pressure, and compound **[2]­Cl** was precipitated from the solution using Et_2_O and subsequently washed with CH_3_Cl. The dark yellow precipitate (152 mg, yield 45%) was characterized. It is completely soluble in all the polar solvents like alcohols, CH_3_CN, DMSO, and H_2_O, and insoluble in CH_3_Cl, hexane, and acetone. Anal. Calcd. for C_36_H_37_Cl_2_N_3_O_4_Os: C, 51.67; H, 4.46; N, 5.02. Found: C, 51.36; H, 4.45; N, 4.92. mp 126–127 °C. IR (cm^–1^): 3064 wbr ν­(O–H), 3030 w, 3010 w, 2964 w, 2934 w ν­(C–H aromatic), 1589 s, 1540 m, 1513 vs ν­(C=C, C=N), 1471 vs, 1455 m, 1392 m, 1370 m, 1277 vs ν­(N–N), 440 m ν­(Os–N), 281 s ν­(Os–Cl). ^1^H NMR (DMSO-*d*_6_, 293 K): δ 0.91 (dd, 6H, CH­(C*H*_3_)_2_ of *p-*cym), 2.36 (s, 3H, CH_3_ of *p-*cym), 2.42 (m, 1H, C*H*(CH_3_)_2_ of *p-*cym), 3.87 (s, 3H, -OC*H*_3_), 3.93 (s, 3H, -OC*H*_3_), 6.27 d, 6.31 d, 6.48 d, 6.50 d (4H, AA′BB′ system, CH_3_–C_6_ *H*_4_–CH­(CH_3_)_2_ of *p-*cym,^3^J = 6 Hz), 6.88–6.96 (m, 3H, C(9′)*H*, C(10′)*H*, C(3′)*H*), 7.18–7.27 (m, 4H, C(9)*H*, C(10)*H*, C(3)*H*, C(6)*H*), 7.38–7.41 (m, 2H, C(4)*H*, C(6′)*H*), 7.54 (s, 1H, C(1)*H*), 7.57 (t, 1H, C(13)*H*, J^3^ = 6 Hz), 7.66 (d, 1H, C(4′)*H*, J^3^ = 16 Hz), 8.14 (d, 1H, C(15)*H*, J^3^ = 8 Hz), 8.29 (t, 1H, C(14)*H*, J^3^ = 8 Hz), 9.31 (t, 1H, C(12)*H*, J^3^ = 6 Hz), 9.63 (s, 1H, -O*H*), 9.68 (s, 1H, -O*H*). ^13^C­{^1^H}-NMR (DMSO-*d*_6_, 293 K): δ 18.9 (−*C*H_3_ of *p-*cym), 22.2, 22.8 (−CH­(*C*H_3_)_2_ of *p-*cym), 31.1 (−*C*H­(CH_3_)_2_ of *p-*cym), 56.1, 56.3 (-O*C*H_3_ of the ligand), 70.9, 73.5 (C­(a-a′)), 77.8, 79.2 (C­(b-b′)), 94.6 (Ci), 100.3 (Ci′), 107.2 (C3), 110.3 (C1), 111.7, 111.8 (C­(6-6′)), 113.5 (C3′), 114.1, 116.1, 122.2, 22.9 (C­(9-9′) and C­(10-10′)), 116.4 (C11), 124.3 (C13), 127.1 (C3′), 139.9 (C­(4-4′)), 143.0 (C14), 146.9 (C2), 148.9 (C­(8-8′)), 149.3 (C­(7-7′)), 155.8 (C12), 156.2 (C2′). {^1^H, ^15^N} HMBC NMR (DMSO-*d*_6_, 293 K): δ_N_ not observed (N_1_-H), (N_2_-H), and (N_3_-H). ESI-MS (+) CH_3_CN (*m*/*z* [relative intensity, %]): 802 [100] [**2**]^+^. Λ_m_ (*T* = 298 K, H_2_O, 10^–3^ mol L^–1^) 125 S cm^2^ mol^–1^.

##### [Ru­(*p-*cym)­(HZPbdcurc)­Cl]Cl ([3]­Cl)

HZPbdcurc (152 mg, 0.4 mmol) and the dimer [Ru­(*p-*cymene)­Cl_2_]_2_ (122 mg, 0.2 mmol) were dissolved in CH_3_CN (5 mL). After 4 h of stirring at room temperature, the compound **[3]­Cl** precipitated from the solution was filtered. The orange precipitate (236 mg, yield 86%) was characterized. It is completely soluble in all the polar solvents like alcohols, DMSO, and partly soluble in H_2_O, and insoluble in CH_3_Cl, CH_3_CN, hexane, and acetone. Anal. Calcd. for C_34_H_33_Cl_2_N_3_O_2_Ru: C, 59.39; H, 4.84; N, 6.11. Found: C, 59.24; H, 4.75; N, 6.06. mp 236–237 °C. IR (cm^–1^): 3064 wbr ν­(O–H), 3018 w, 2985 w, 2962 w, 2924 w, 2877 w ν­(C–H aromatic), 1604 s, 1585 m, 1574 m, 1551 m, 1512 s ν­(C=C, C=N), 1479 vs, 1447 m, 1397 m, 1355 m, 1272 vs ν­(N–N), 461 m ν­(Ru–N), 292 s ν­(Ru–Cl). ^1^H NMR (DMSO-*d*_6_, 293 K): δ 0.96 (d, 6H, CH­(C*H*_3_)_2_ of *p-*cym, J^3^ = 7 Hz), 2.29 (s, 3H, CH_3_ of *p-*cym), 6.06 d, 6.10 d, 6.20 d, 6.29 d (4H, AA′BB′ system, CH_3_–C_6_ *H*_4_–CH­(CH_3_)_2_ of *p-*cym,^3^J = 6 Hz), 6,88 (d, 2H, C­(6-6′)*H*, J^3^ = 9 Hz), 6.94 (d, 2H, C­(10-10′)*H*, J^3^ = 9 Hz), 7.02 (d, 1H, C(3)*H*, J^3^ = 16 Hz), 7.62–7.64 (m, 6H, C­(7-7′)*H*, C­(9-9′)*H*, C(3)*H*, C(4)*H* and C(13)*H*), 7.68 (d, 1H, C(4)*H*, J^3^ = 16 Hz), 7.99 (d, 1H, C(15)*H*, J^3^ = 9 Hz), 8.27 (t, 1H, C(14)*H*, J^3^ = 8 Hz), 9.35 (d, 1H, C(12)*H*, J^3^ = 6 Hz), 10.05 (sbr, 2H, −O*H*). ^13^C­{^1^H}-NMR (DMSO-*d*_6_, 293 K): δ 19.1 (−*C*H_3_ of *p-*cym), 21.9, 22.3 (−CH­(*C*H_3_)_2_ of *p-*cym), 30.9 (−*C*H­(CH_3_)_2_ of *p-*cym), 81.5, 83.2 (C­(b-b′)), 86.0, 87.4 (C­(a-a′)), 103.7 (Ci), 106.5 (Ci′), 107.5, 123.8, 130.1 (C13, C4, C3, C­(7-7′) and C­(9-9′)), 110.5 (C5′), 113.7 (C3), 114.0 (C15), 116.3, 116.5 (C­(6-6′) and C­(10-10′)), 139.3 (C4′), 142.8 (C14), 147.1 (C2′), 148.6 (C11), 155.7 (C12), 157.4 (C2), 159.7 (C­(8-8′)). {^1^H, ^15^N} HMBC NMR (DMSO-*d*_6_, 293 K): δ_N_ 217.5 (N_1_-H), 210.7 (N_2_-H), 214.2 (N_3_-H). ESI-MS (+) CH_3_CN (*m*/*z* [relative intensity, %]): 652 [100] [**3**]^+^. Λ_m_ (*T* = 298 K, H_2_O, 10^–3^ mol L^–1^) 120 S cm^2^ mol^–1^.

##### [Os­(*p-*cym)­(HZPbdcurc)­Cl]Cl ([4]­Cl)

HZPbdcurc (152 mg, 0.4 mmol) and dimer [Os­(*p-*cymene)­Cl_2_]_2_ (120 mg, 0.2 mmol) were dissolved in CH_3_CN (5 mL). After 4 h of stirring at room temperature, the compound **[4]­Cl** precipitated from the solution was filtered. The dark yellow precipitate (190 mg, yield 61%) was characterized. It is completely soluble in all the polar solvents like alcohols, DMSO, and partly soluble in H_2_O and insoluble in CH_3_Cl, CH_3_CN, hexane, and acetone. Anal. Calcd. for C_34_H_33_Cl_2_N_3_O_2_Os: C, 52.57; H, 4.28; N, 5.41. Found: C, 52.46; H, 4.21; N, 5.32. mp 199–200 °C. IR (cm^–1^): 3036 mbr ν­(O–H), 3009 mbr, 2965 w, 2926 w, 2868 w, 2798 w ν­(C–H aromatic), 1603 vs, 1583 m, 1572 w, 1542 m, 1512 s ν­(C=C, C=N), 1476 vs, 1445 s, 1397 m, 1274 vs ν­(N–N), 461 m ν­(Os–N), 282 s ν­(Os–Cl). ^1^H NMR (DMSO-*d*_6_, 293 K): δ 0.91 (dd, 6H, CH­(C*H*_3_)_2_ of *p-*cym), 2.36 (s, 3H, CH_3_ of *p-*cym), 2.41 (m, 1H, C*H*(CH_3_)_2_ of *p-*cym), 6.27 d, 6.32 d, 6.45 d, 6.49 d (4H, AA′BB′ system, CH_3_–C_6_ *H*_4_–CH­(CH_3_)_2_ of *p-*cym,^3^J = 6 Hz), 6.88 (d, 2H, C­(6-6′)*H*, J^3^ = 9 Hz), 6,93 (d, 2H, C­(10-10′)*H*, J^3^ = 9 Hz), 7.41 (d, 1H, C(3)*H*, J^3^ = 16 Hz), 7,49 (d, 1H, C(4)*H*, J^3^ = 16 Hz), 7.56 (t, 1H, C(13)*H*, J^3^ = 7 Hz), 7.62–7.67 (m, 6H, C­(7-7′)*H*, C­(9-9′)*H*, C(4′)*H* and C(3′)*H*), 8.13 (d, 1H, C(15)*H*, J^3^ = 9 Hz), 8.27 (t, 1H, C(14)*H*, J^3^ = 8 Hz), 9.30 (d, 1H, C(12)*H*, J^3^ = 6 Hz), 10.09 (sbr, 2H, −O*H*). ^13^C­{^1^H}-NMR (DMSO-*d*_6_, 293 K): δ 18.95 (−*C*H_3_ of *p-*cym), 22.27, 22.77 (−CH­(*C*H_3_)_2_ of *p-*cym), 31.1 (−*C*H­(CH_3_)_2_ of *p-*cym), 70.8, 73.4 (C­(a-a′)), 77.9, 79.2 (C­(b-b′)), 94.6 (Ci), 100.4 (Ci′), 107.1, 130.2, 139.7 (C4′, C3′, C­(7-7′) and C­(9-9′)), 110.0, 128.6 (C3 and C4), 113.8 (C15), 116.3, 116.5 (C­(6-6′) and C­(10-10′)), 124.3 (C13), 130.0, 130.2 (C5 and C5′), 143.0 (C14), 146.8 (C2), 148.6 (C11), 155.7 (C12), 157.4 (C2), 1148.9 (C11), 155.8, 156.2 (C12 and C­(8-8′)), 159.8 (C2′). {^1^H, ^15^N} HMBC NMR (DMSO-*d*_6_, 293 K): δ_N_ 212.2 (N_1_-H), 201.5 (N_2_-H), not observed (N_3_-H). ESI–MS (+) CH_3_CN (*m*/*z* [relative intensity, %]): 742 [100] [**4**]^+^. Λ_m_ (*T* = 298 K, H_2_O, 10^–3^ mol L^–1^) 129 S cm^2^ mol^–1^.

### X-ray Crystallography

Crystallographic data and structure refinement details for **[1]­Cl** and **[3]­Cl** are given in Table S2. They crystallize in the triclinic *P* 1̅ and orthorhombic *Pca*2_1_ space groups, respectively. Suitable orange crystals were selected and mounted on a SuperNova, Dual, AtlasS2 diffractometer (**[1]­Cl**) or on an XtaLAB Synergy R, DW system, HyPix-Arc 150 diffractometer (**[3]­Cl**), where intensities were collected at 140 K using Cu Kα radiation. The data sets were reduced and corrected for absorption using CrysAlis^Pro^.^35^ The structures were solved with the ShelXT solution program^36^ using dual methods and by using Olex2 1.5 as the graphical interface.^37^ All non-hydrogen atoms were refined anisotropically using full-matrix least-squares based on |*F*|.^2^ Hydrogen atoms were placed at calculated positions using the ‘riding’ model. The CCDC numbers 2378944 and 2378945 contain the crystallographic data for compounds **[1]­Cl** and **[3]­Cl**, respectively. These data can be obtained free of charge via http://www.ccdc.cam.ac.uk/data_request/cif.

### Computational Details

The electronic structure and geometries of the HZPcurc and HZPbdcurc ligands were calculated using DFT at the B3LYP level^38,39^ with the 6-311G* basis set. Similarly, ruthenium and osmium complexes **[1]­Cl**-**[4]­Cl** were also calculated by using DFT at the B3LYP level. The Ru and Os ions were described with the LANL2DZ basis set,^40^ while the 6-31G* basis set was used for the other atoms. Molecular geometries of **[1]­Cl** and **[3]­Cl** were optimized by starting from the crystallographic coordinates. Frequency calculations were carried out at the same level of theory to identify all of the stationary points as minima (zero imaginary frequencies). DFT calculations were performed with the Gaussian 09 suite of programs.^41^ The theoretical IR spectra were scaled by a factor of 0.96.^42,43^ The coordinates of all optimized compounds are collected in a separate associated XYZ file attached to the Supporting Information.

#### Cell Culture and Cytotoxicity Tests

All cell lines were grown in a humidified 5% CO_2_ environment at 37 °C in dedicated media. Growth media comprised minimum essential medium (MEM) supplemented with 10% FBS, 1% sodium pyruvate, antibiotics, and antimycotic agents for MCF7. MCF10A cells were cultured in a DMEM/F12 Ham’s mixture supplemented with 5% equine serum, 20 ng/mL EGF, 10 μg/mL insulin, 0.5 mg/mL hydrocortisone, antibiotics, and antimycotics. MCF7-CR cells were grown in minimum essential medium (MEM), 10% FBS supplemented with 0.1 mg/mL cisplatin, sodium pyruvate, antibiotics, and antimycotics. A2780 and A2780cis cells were cultured in RPMI 1640 GlutaMAX, and HEK293T cells were cultured in DMEM GlutaMAX medium containing 10% heat-inactivated FBS and antibiotics. The effect exerted on cell viability by the compounds was determined using the 3-(4,5-dimethylthiazol-2-yl)-2,5-diphenyltetrazolium bromide (MTT) assay.^44^ After individual treatments with 0–100 μM of the compound for 72 h, MTT was added to the culture media at a final concentration of 0.5 mg/mL and incubated for 2 h at 37 °C. Cisplatin was used as a positive control. The medium was replaced with 100 μL of DMSO, and after 10 min, the optical density was recorded at 550 nm on a microplate reader. At least six cultures were used for each data point. Chemicals (Merck-Sigma, Milan, Italy) and plasticware (Corning, Milan, Italy) were cell culture grade.

#### Binding to DNA

The binding kinetics/affinities of the molecules of interest for DNA were studied according to a previously described biosensor-based method. Briefly, streptavidin was anchored to a carboxylate surface via EDC/NHS chemistry,^45^ and 5′-biotinylated dsDNA oligomer (sequence: 3′-CCACCCACTACCCTGGTTGGATGCTAATGT-5′) was coupled to surface-blocked streptavidin. Compounds were independently added to the DNA-coated surface at different concentrations, each time following binding kinetics up to equilibrium. PBS buffer was used for both the dissociation and the regeneration steps. Experimental data were fitted to both mono- and biexponential models, and the validity of each model was assessed by a standard F-test procedure. The binding sites on DNA were mapped according to independent competitive binding assays individually using a minor groove binder (GelRed) and a major groove binder (methyl green), respectively.^46^ Briefly, GelRed displacement was monitored by comparing the emission spectra of solutions containing different concentrations of curcuminoid-like ligands/complexes (0–100 μM), 20 μM DNA, and GelRed (10 μM) in 10 mM phosphate buffer, pH 7.4. Similarly, methyl green displacement assay was performed by monitoring the absorbance at 630 nm upon addition of candidate competitors (0–100 μM), 100 μM DNA, and methyl green (10 μM) in 50 mM Tris–HCl buffer and 7.5 mM MgSO_4_, pH 7.5.

#### Effect on Proteasome Activity

The effect of the compounds on the activity of the isolated 20S proteasome was tested using specific synthetic fluorogenic peptide substrates (Suc-Leu-Leu-Val-Tyr-AMC, Z-Leu-Ser-Thr-Arg-AMC, and Z-Leu-Leu-Glu-AMC for chymotrypsin-like, trypsin-like, and peptidyl glutamyl peptide hydrolase activity, respectively) as reported elsewhere.^45^ Briefly, individual mixtures consisting of the compounds (0–100 μM), 1 μg of isolated 20S proteasome, the specific substrate, and 50 mM Tris–HCl pH 8.0 up to a final volume of 100 μL were incubated at 37 °C, and after 1 h, the fluorescence measurements of the hydrolyzed AMC were recorded on a SpectraMax Gemini XPS microplate reader (λ_exc_ = 365 nm, λ_em_ = 449 nm).

#### Immunometric Quantification of p62

Semiquantitative analysis of p62 was performed by Western blotting upon individual 24 h treatments of MCF-7 cells with 10 μM of HZPbdcurc or [Ru­(*p-*cym)­(HZPbdcurc)­Cl]­Cl. Treated cells were lysed, and 15 μg of total protein was electrophoresed on 12% SDS-PAGE, then protein bands were electroblotted onto PVDF membranes (Millipore—Milan, Italy). After incubation with anti-p62 monoclonal antibody (Merck Sigma—Milan, Italy), the bands were immunodetected on a ChemiDoc MP imaging system (Bio-Rad - Milan, Italy). Molecular weight protein markers (range: 6.5–205 kDa) were included in each gel; glyceraldehyde-3-phosphate dehydrogenase (GAPDH) was used as a control for equal protein loading. Western blot results were analyzed using Fiji software.^47^

## Supplementary Material





## Notes

All the crystallographic details; DFT calculations; monodimensional ^1^H- and ^13^C NMR spectra and bidimensional {^1^H–^1^H}-COSY, {^1^H–^13^C}-HSQC, {^1^H–^13^C}-HMBC, {^1^H–^15^N}-HSQC, {^1^H–^15^N}-HMBC NMR spectra (PDF)
Coordinates of all optimized compounds (XYZ)
