# Correction to “Half-Sandwich Ruthenium and Osmium Complexes with Hydrazinocurcuminoid-like Ligands”

**DOI:** 10.1021/acs.organomet.5c00319

**Published:** 2025-09-05

**Authors:** Noemi Pagliaricci, Riccardo Pettinari, Fabio Marchetti, Sara Pagliaricci, Massimiliano Cuccioloni, Anna Maria Eleuteri, Agustín Galindo, Farzaneh Fadaei-Tirani, Kseniya Glinkina, Paul J. Dyson

The image of [1]Cl presented in the originally published version of Figure 3 was uncorrected due to an oversight in the editing of the MATLAB script used to generate the figure. The corrected version of Figure 3 is provided here.

**Figure 3:**
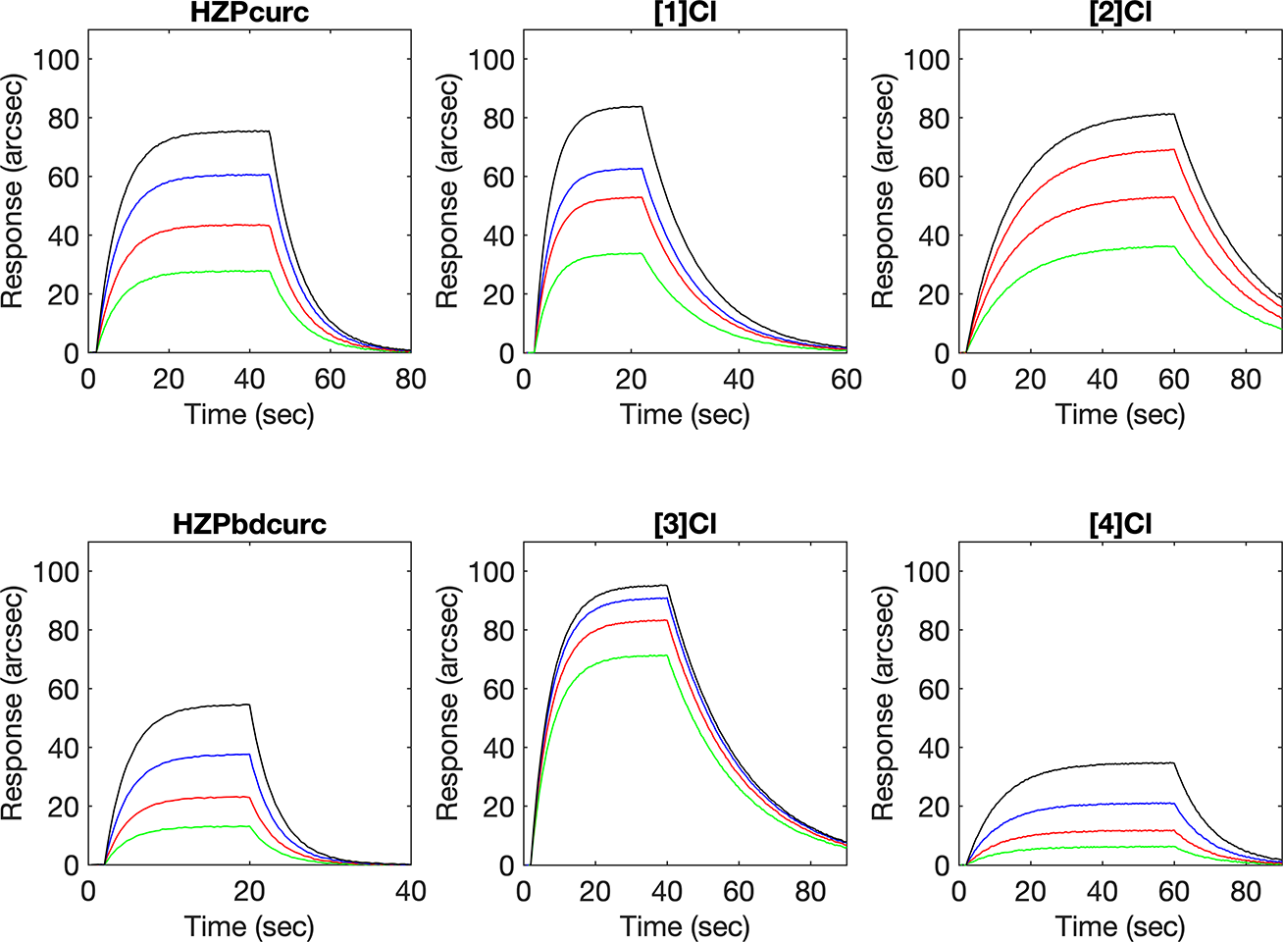
Representative superimposition of sensorgrams obtained upon the binding of soluble curcuminoid ligands and metal complexes to a surface-anchored dsDNA oligomer.

