# Supplementary material for: Half-Sandwich Ruthenium and Osmium Complexes with Hydrazinocurcuminoid-like Ligands
Source: Organometallics. 2025 May 16;44(11):1155–64. doi: 10.1021/acs.organomet.5c00082 (PMC12153055; doi:10.1021/acs.organomet.5c00082)
Supplement: Supplementary file 1 [file om5c00082_si_001.pdf]

Supporting Information

Half-Sandwich Ruthenium and Osmium Complexes with Hydrazinocurcuminoid-Like Ligands

Noemi Pagliaricci,[a] Riccardo Pettinari,[a] Fabio Marchetti,[b] Sara Pagliaricci,[a] Massimiliano Cuccioloni,[c] Anna Maria Eleuteri, [c] Agustín Galindo,[d] Farzaneh Fadaei-Tirani,[e] Kseniya Glinkina,[e] Paul J. Dyson.[e]*

[a] School of Pharmacy, [b]School of Science and Technology, and [c]School of Biosciences and Veterinary Medicine, University of Camerino, via Madonna delle Carceri, 62032 Camerino, MC, Italy; [d]Departamento de Química Inorgánica, Facultad de Química, Universidad de Sevilla, Aptdo 1203, 41071 Sevilla, Spain; [e] Institut des Sciences et Ingénierie Chimiques, École Polytechnique Fédérale de Lausanne (EPFL), 1015 Lausanne, Switzerland. riccardo.pettinari@unicam.it

Index

SPECTROSCOPIC CHARACTERISATION **S4**

Figure S1. ^{1}H-NMR of **HZPcurc** in DMSO-d_6 ..... S4
Figure S2. ^{13}C-NMR of **HZPcurc** in DMSO-d_6 ..... S5
Figure S3. {^{1}H-^{15}N }-HMBC NMR of **HZPcurc** in DMSO-d_6 ..... S6
Figure S4. ^{1}H-NMR of **HZPbdcurc** in DMSO-d_6 ..... S7
Figure S5. ^{13}C-NMR of **HZPbdcurc** in DMSO-d_6 ..... S8
Figure S6. {^{1}H-^{15}N }-HMBC NMR of **HZPbdcurc** in DMSO-d_6 ..... S9
Figure S7. ^{1}H-NMR of **[1]Cl** in DMSO-d_6 ..... S10
Figure S8. Magnification of ^{1}H-NMR S11
Figure S9. ^{13}C-NMR of **[1]Cl** in DMSO-d_6 ..... S12
Figure S10. {^{1}H-^{15}N}-HMBC NMR of **[1]Cl** in DMSO-d_6 ..... S13
Figure S11. ^{1}H-NMR of **[2]Cl** in DMSO-d_6 ..... S14
Figure S12. ^{13}C-NMR of **[2]Cl** in DMSO-d_6 ..... S15
Figure S13. ^{1}H-NMR of **[3]Cl** in DMSO-d_6 ..... S16
Figure S14. ^{13}C-NMR of **[3]Cl** in DMSO-d_6 ..... S17
Figure S15. {^{1}H-^{15}N}-HMBC NMR of **[3]Cl** in DMSO-d_6 ..... S18
Figure S16. ^{1}H- NMR of **[4]Cl** in DMSO-d_6 ..... S19
Figure S17. ^{13}C- NMR of **[4]Cl** in DMSO-d_6 ..... S20
Figure S18. {^{1}H-^{15}N}-HMBC NMR of **[4]Cl** in DMSO-d_6 ..... S21
Figure S19. Comparison of ^{1}H-NMR spectra of ligand **HZPcurc** and complexes **[1]Cl**, **[3]Cl** in DMSO-d_6 ..... S22
Figure S20. Comparison of ^{1}H-NMR spectra of ligand **HZPbdcurc** and complexes **[2]Cl**, **[4]Cl** in DMSO-d_6 .. S23
Figure S21. ESI-MS of **[1]**$^{+}$ in CH_3CN ([M-35] at 712 m/z) S24
Figure S22. ESI-MS of **[2]**$^{+}$ in CH_3CN ([M-35] at 802 m/z) S25
Figure S23. ESI-MS of **[3]**$^{+}$ in CH_3OH ([M-35] at 652 m/z) S26
Figure S24. ESI-MS of **[4]**$^{+}$ in CH_3OH ([M-35] at 742 m/z) S27

CRYSTALLOGRAPHY **S28**

Table S1. Selected structural parameters of **[1]Cl** and **[3]Cl**. S28
Table S2. Crystal data and structure refinement for complexes **[1]Cl** and **[3]Cl**. S30
Figure S25. Top: crystal packing of **[1]Cl**. Bottom: π-stacking interaction in **[1]Cl**. S31
Figure S26. Asymmetric unit of **[3]Cl**. S32
Figure S27. Top: bifurcated hydrogen bonds in **[3]Cl**. Bottom: packing in **[3]Cl** involving intercalation of HZPbdcurc ligands. S33
Figure S28. Crystal packing of **[3]Cl**. S34

THEORETICAL STUDIES **S35**

Figure S29. Optimized structures of **HZPcurc** (top) and **HZPbdcurc** (bottom). S35
Table S3. Selected calculated structural parameters of **HZPcurc** and **HZPbdcurc**. S36
Figure S30. Selected molecular orbitals of HZPcurc and HZPbdcurc. S37
Table S4. Selected structural parameters (bond distances, Å and angles, °) of complexes **[1]Cl-[4]Cl** and comparison with experimental data for **[1]Cl** and **[3]Cl** (red numbers). S38
Figure S31. Optimized structures of complexes **[1]**$^{+}$-**[4]**$^{+}$. S39
Figure S32. Selected molecular orbitals of **[1]Cl**. S40

STABILITY STUDIES **S41**

Figure S33. UV-Visible spectra for the stability studies of **[1]Cl-[4]Cl** under physiological conditions. S41

MOLECULAR DOCKING **S42**

Figure S34. Visualization of the best scoring complexes formed upon docking the HZPcurc and HZPbdcurc ligands and the corresponding Ru and Os complexes on a dsDNA (solid grey surface). The nucleotides of the minor groove involved in the formation of H-bonds with the molecules of interest are coloured in light blue. Images were rendered with PyMOL 3.0.3. ..... S43

TABLE S5: COMPUTED ΔG VALUES, NUMBER AND TYPES OF INTERACTION FOR THE COMPLEXES WITH DSDNA OBTAINED BY MOLECULAR DOCKING. S44
FIGURE S35. DECREASE IN THE FLUORESCENCE EMISSION AT 500 NM OF THE COMPLEX BETWEEN DNA-AND GELRED (A MINOR GROOVE BINDER) IN THE PRESENCE OF INCREASING CONCENTRATIONS OF THE HZPCURC AND HZPBDCURC LIGANDS AND CORRESPONDING OS AND RU COMPLEXES........ S45

Spectroscopic characterisation

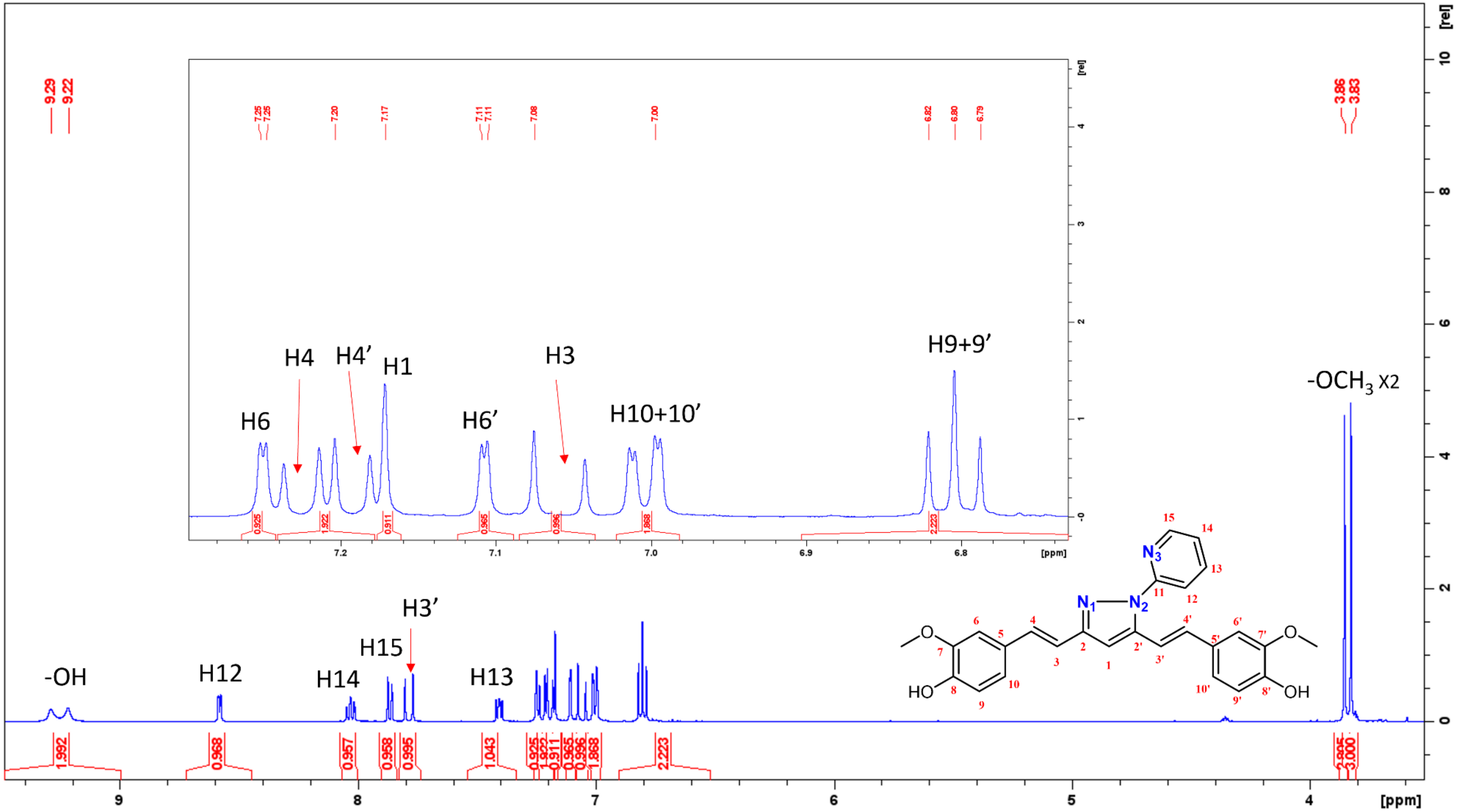

Figure S1. ^{1}H-NMR of **HZPcurc** in DMSO-d_6

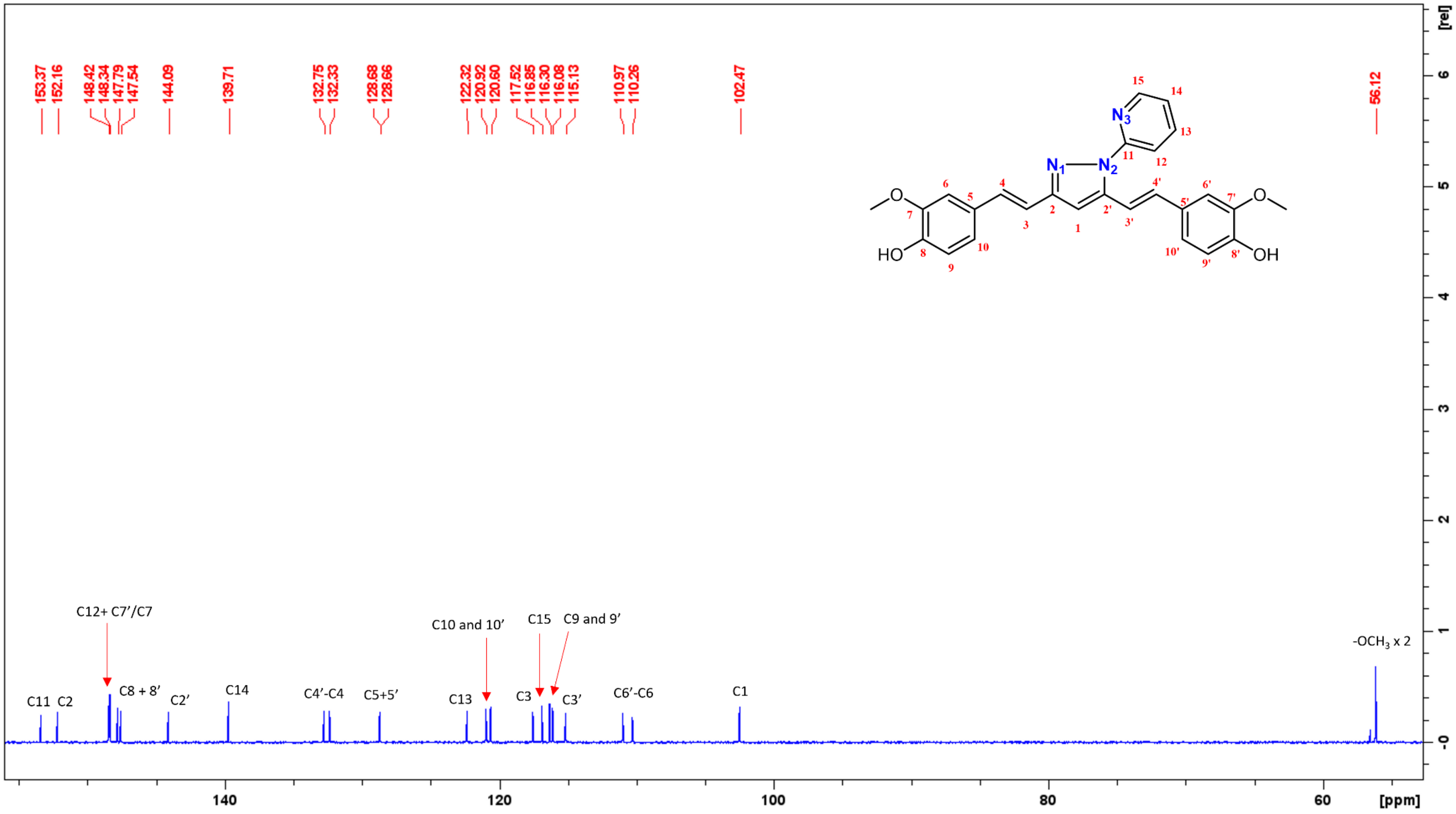

Figure S2. ^{13}C-NMR of **HZPcurc** in DMSO-d_6

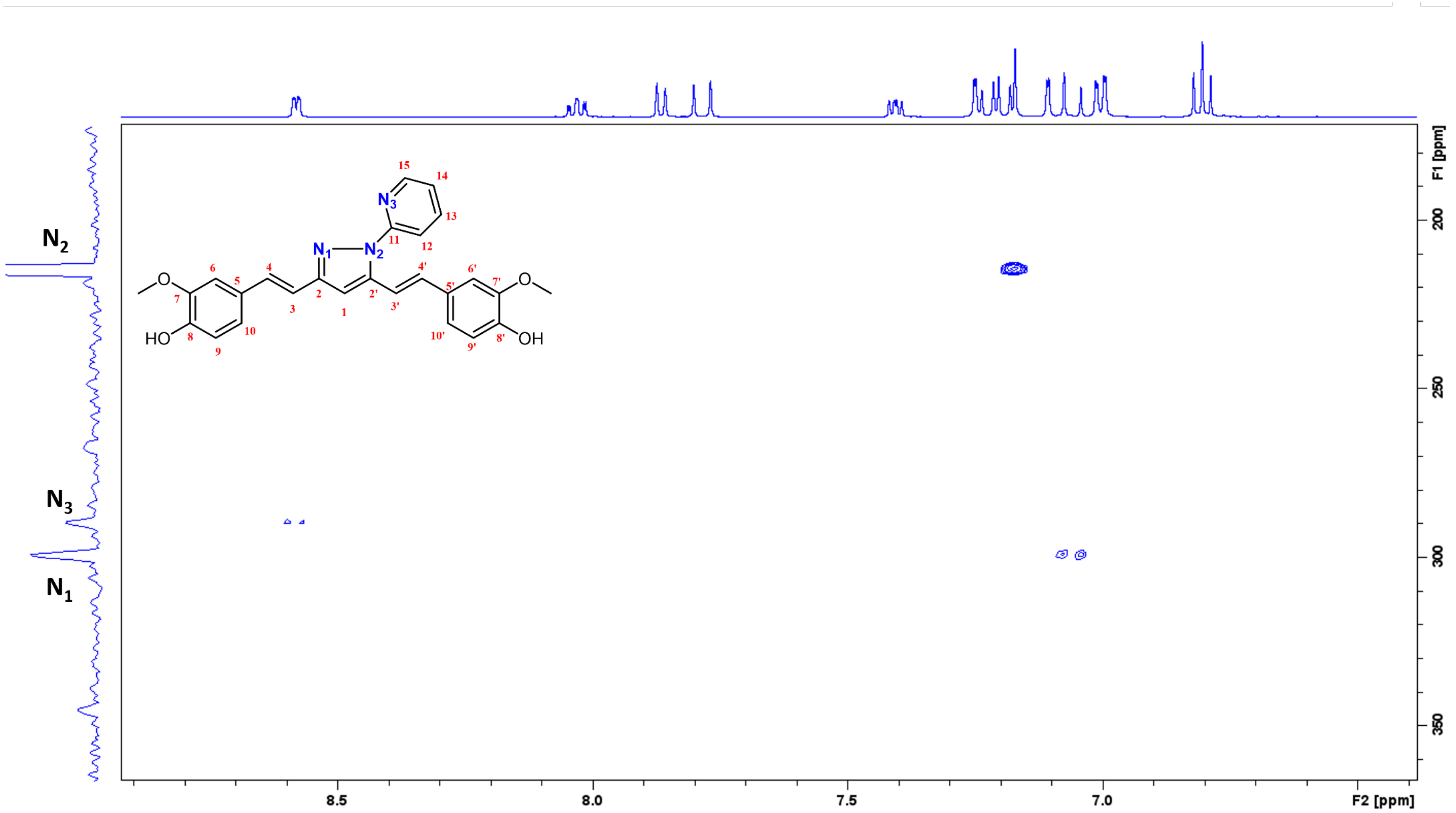

Figure S3. {^{1}H-^{15}N }-HMBC NMR of **HZPcurc** in DMSO-d_6

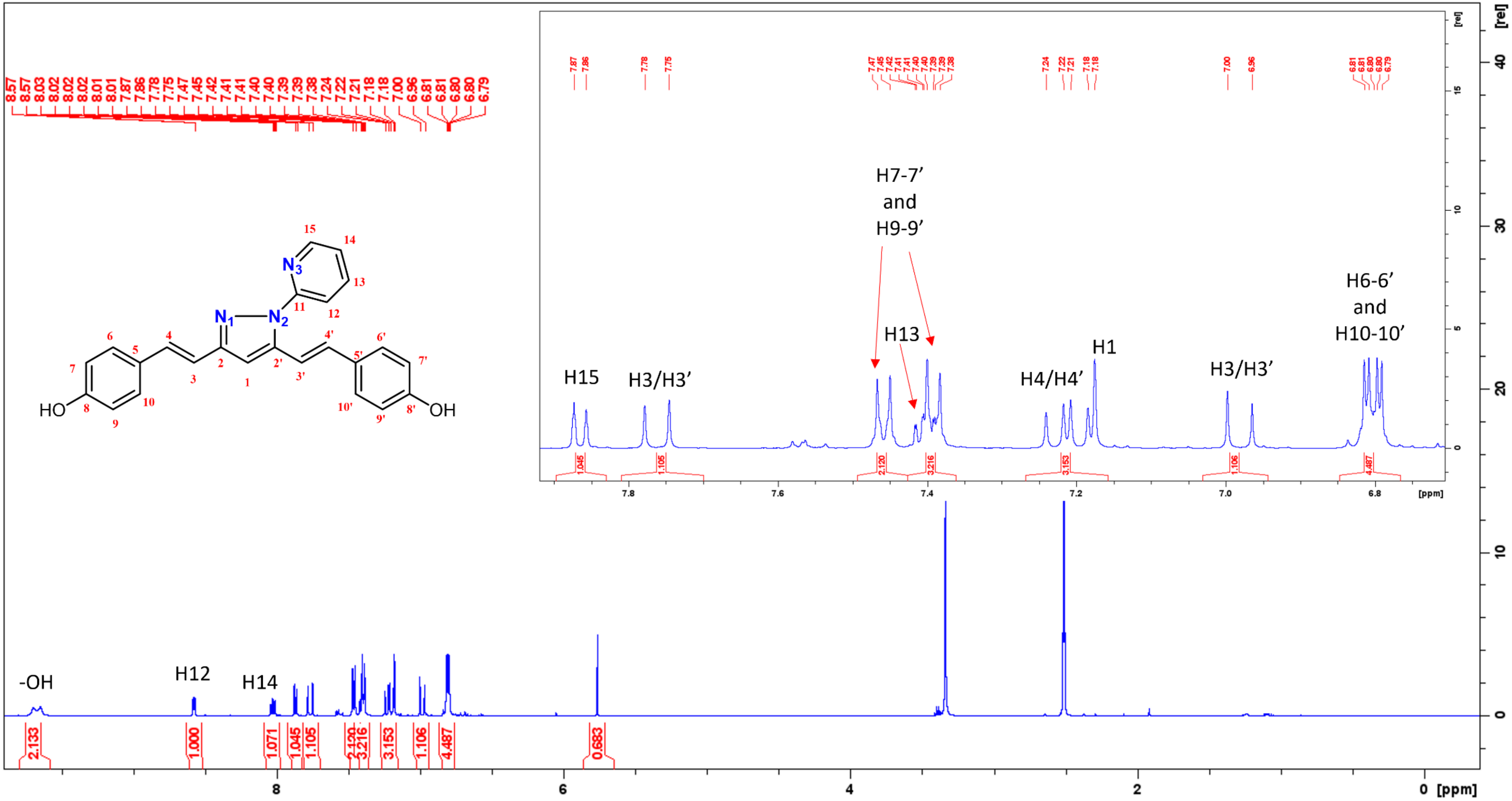

Figure S4. ^{1}H-NMR of **HZPbdcurc** in DMSO-d_6

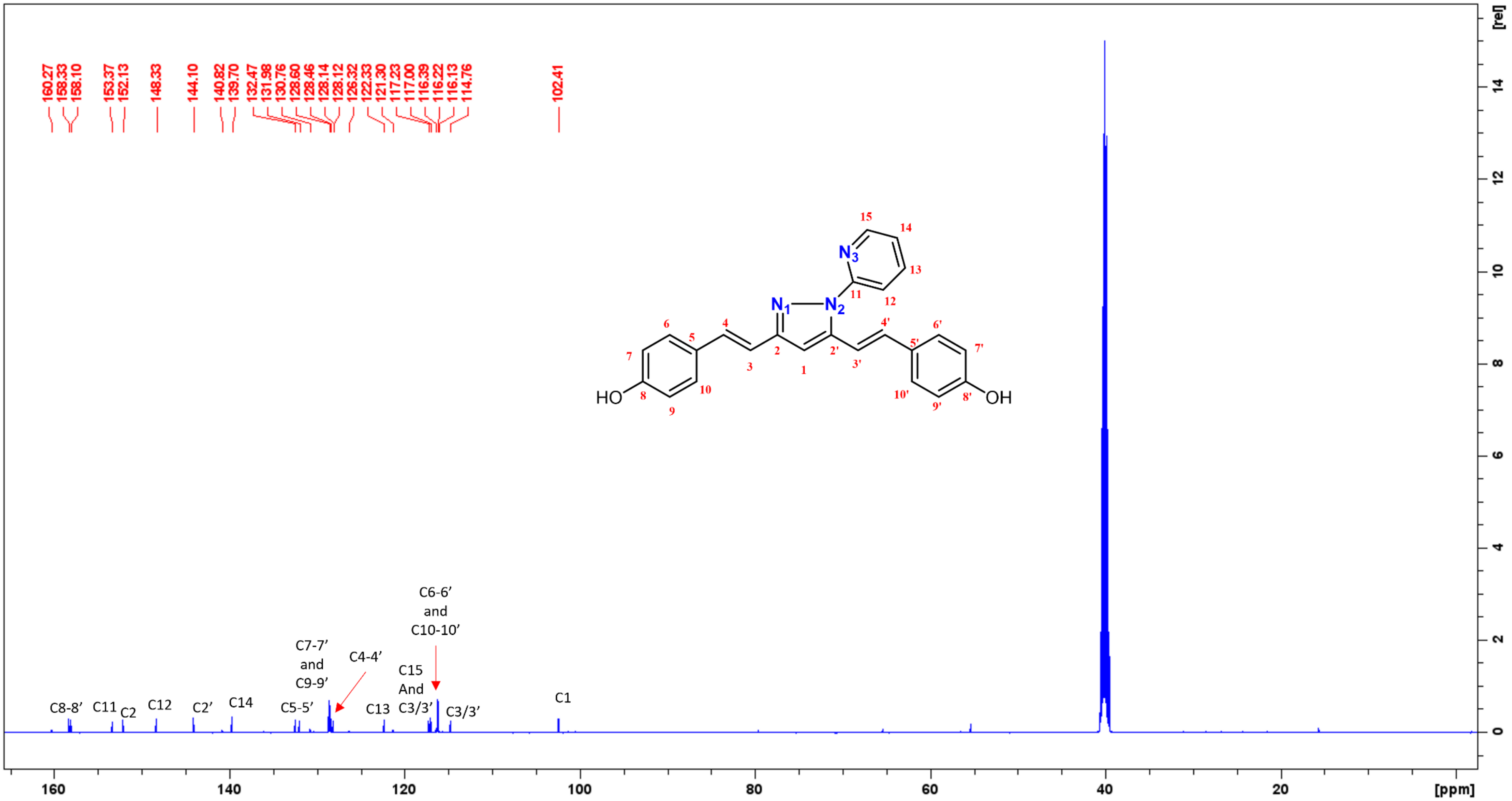

Figure S5. ^{13}C-NMR of **HZPbdcurc** in DMSO-d_6

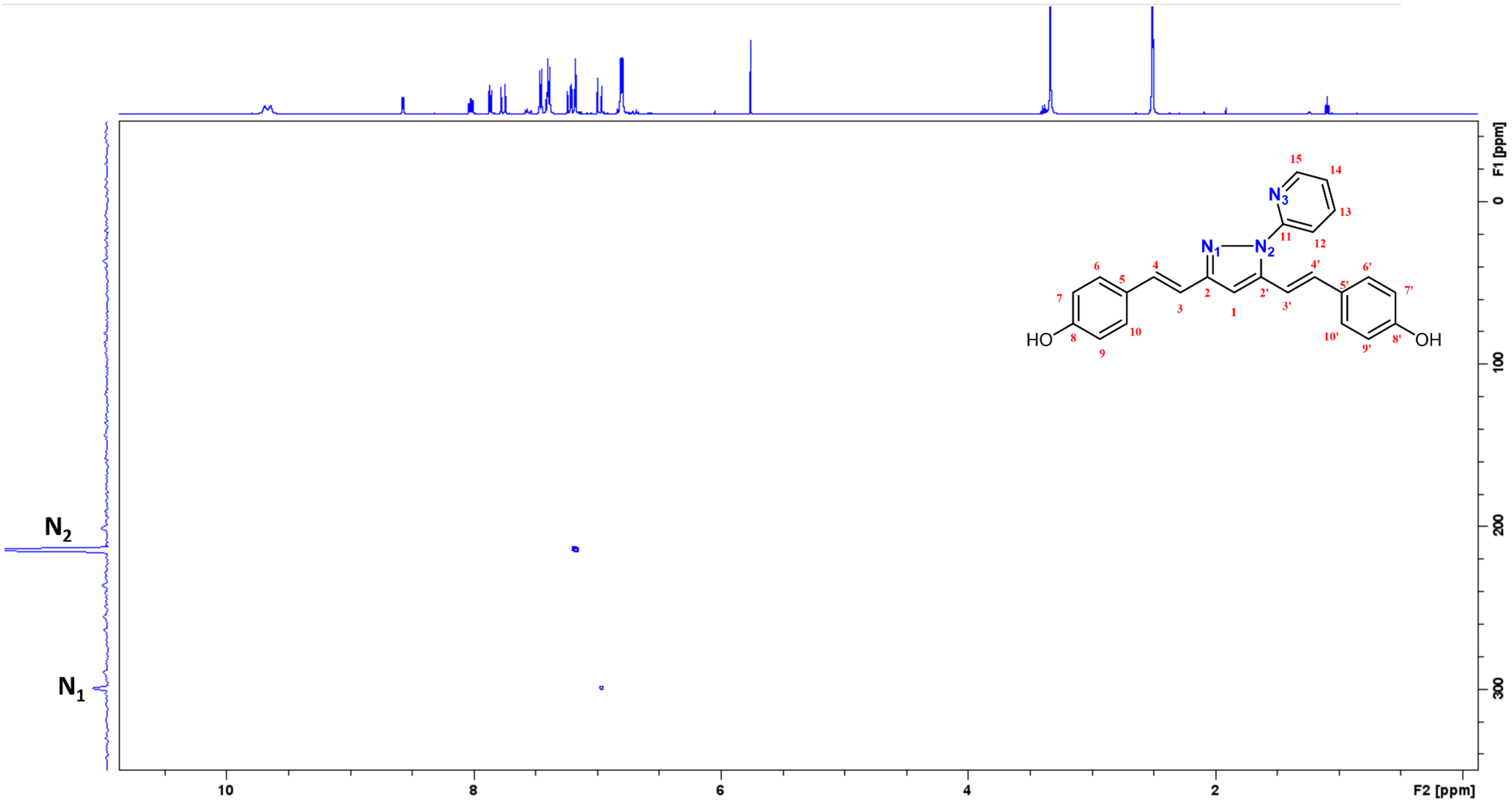

Figure S6. {^{1}H-^{15}N }-HMBC NMR of **HZPbdcurc** in DMSO-d_6

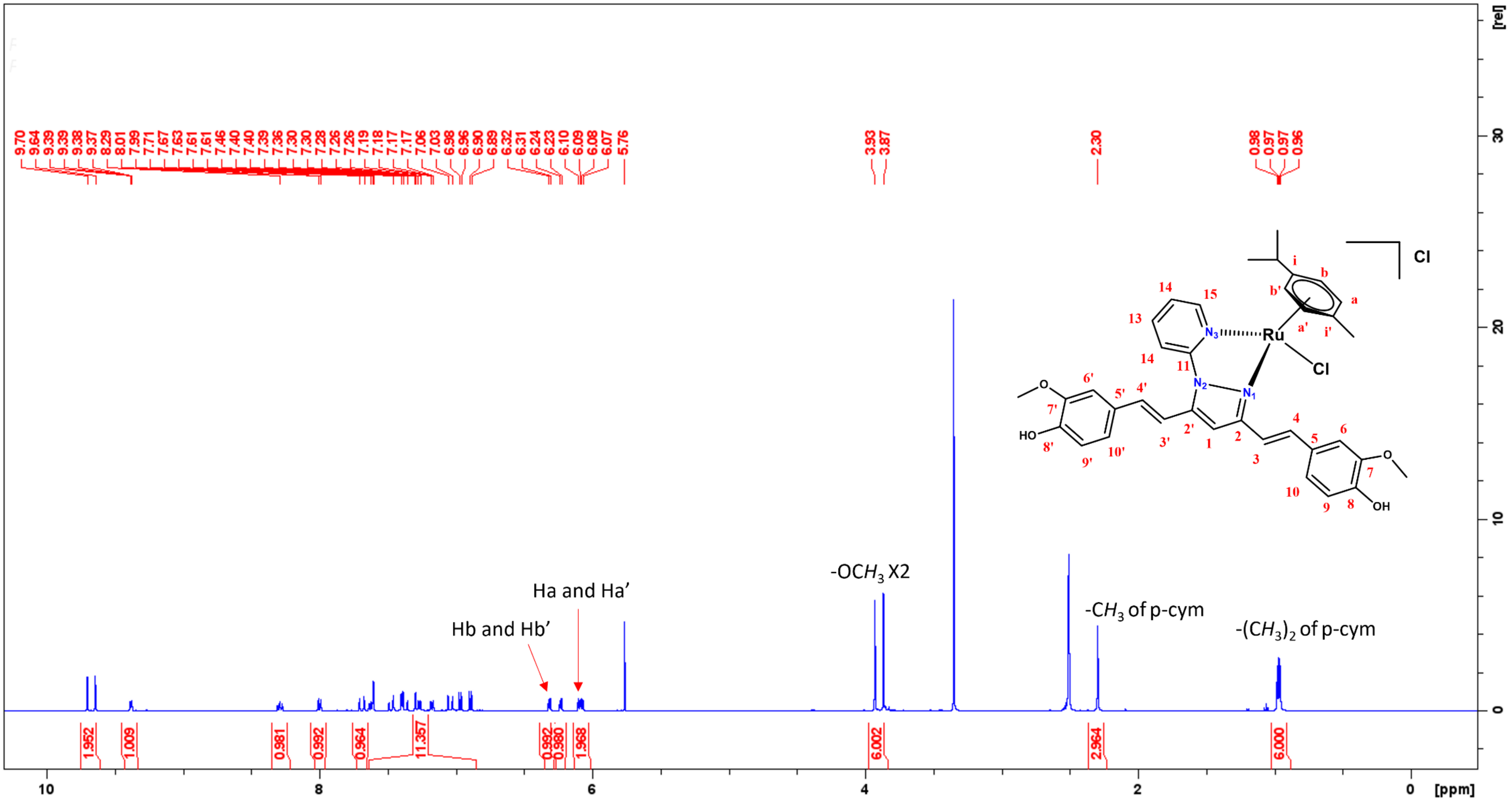

Figure S7. ^{1}H-NMR of **[1]Cl** in DMSO-d_6

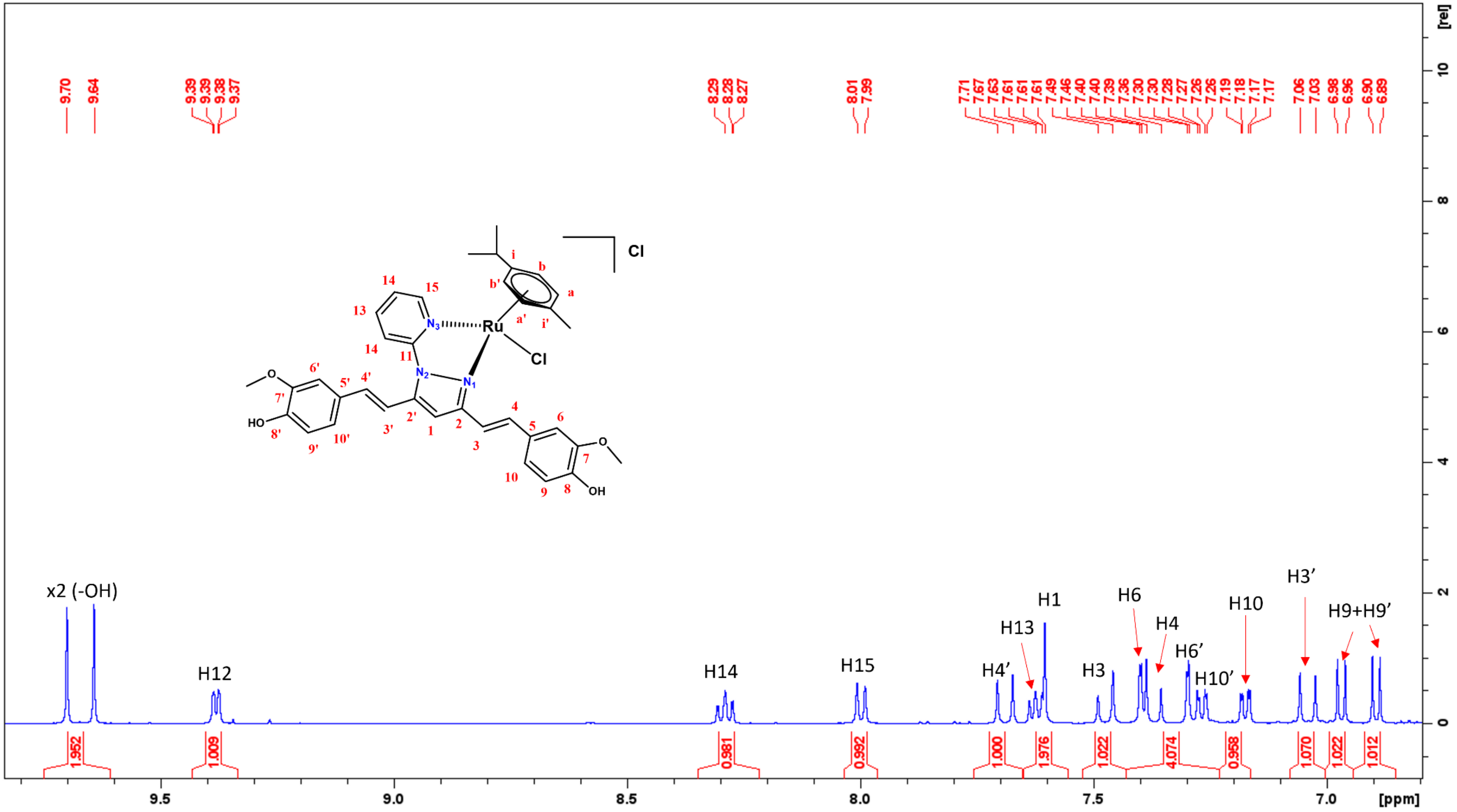

Figure S8. Magnification of ^{1}H-NMR

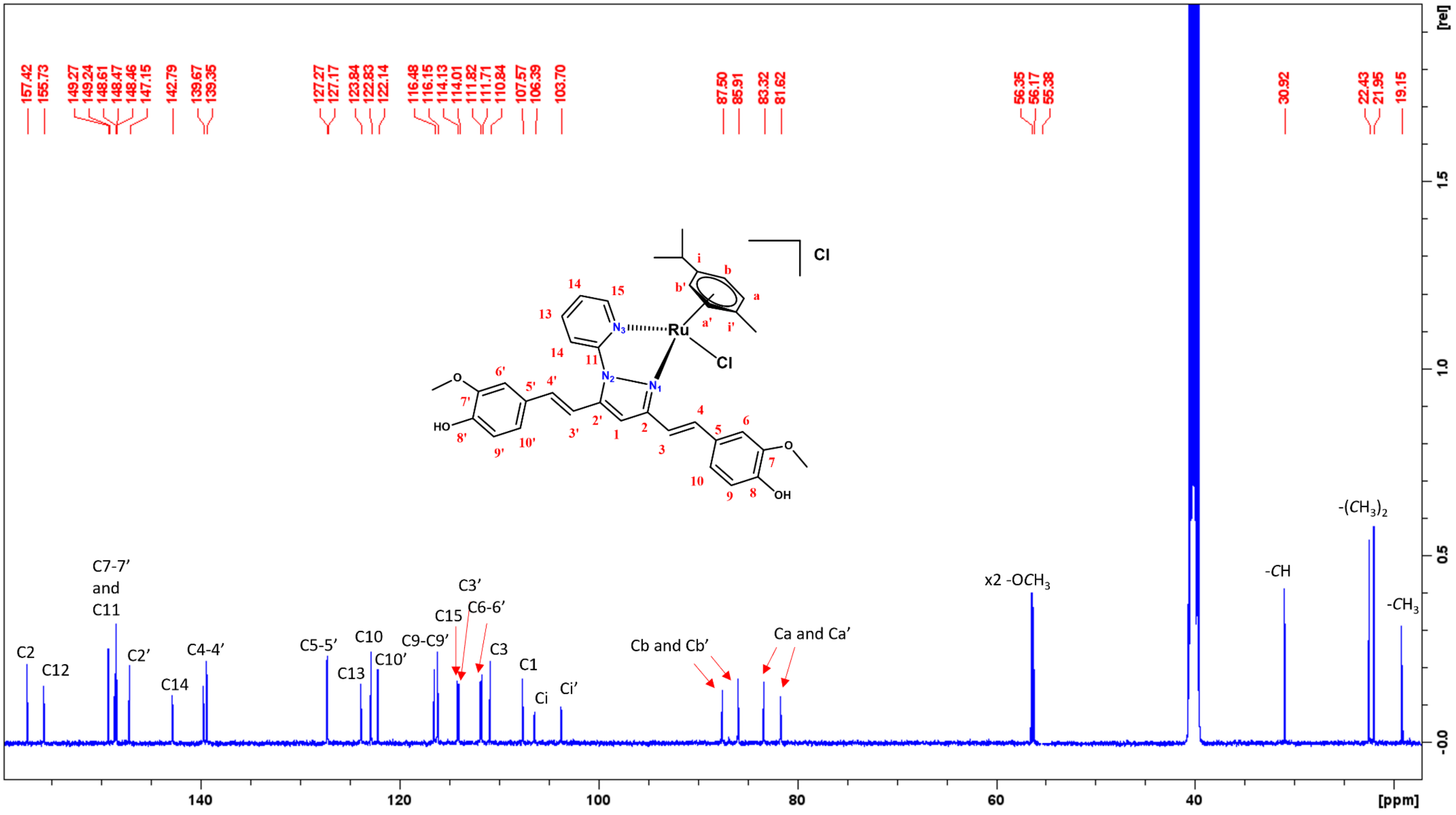

Figure S9. ^{13}C-NMR of **[1]Cl** in DMSO-d_6

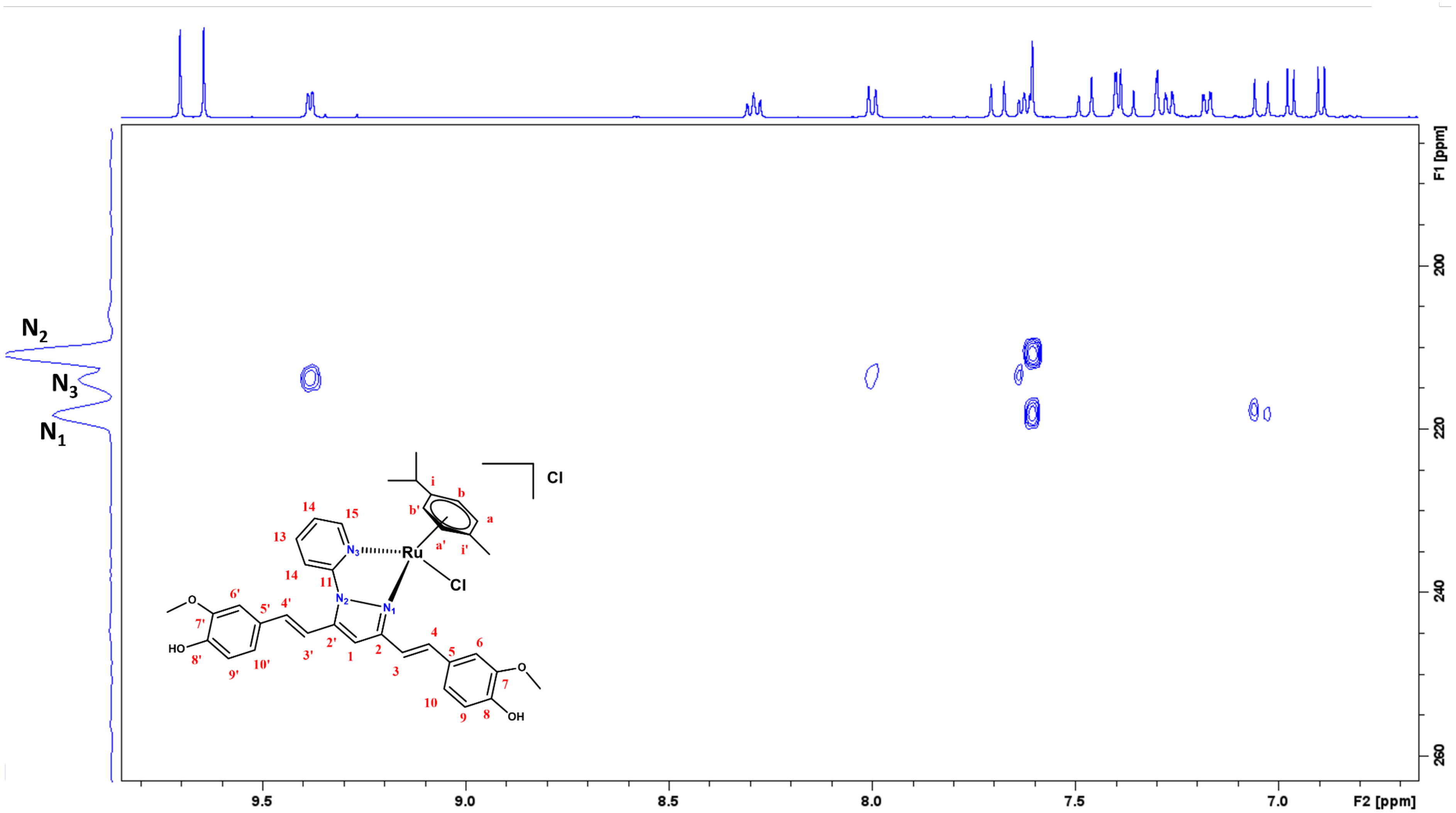

Figure S10. {^{1}H-^{15}N}-HMBC NMR of **[1]Cl** in DMSO-d_6

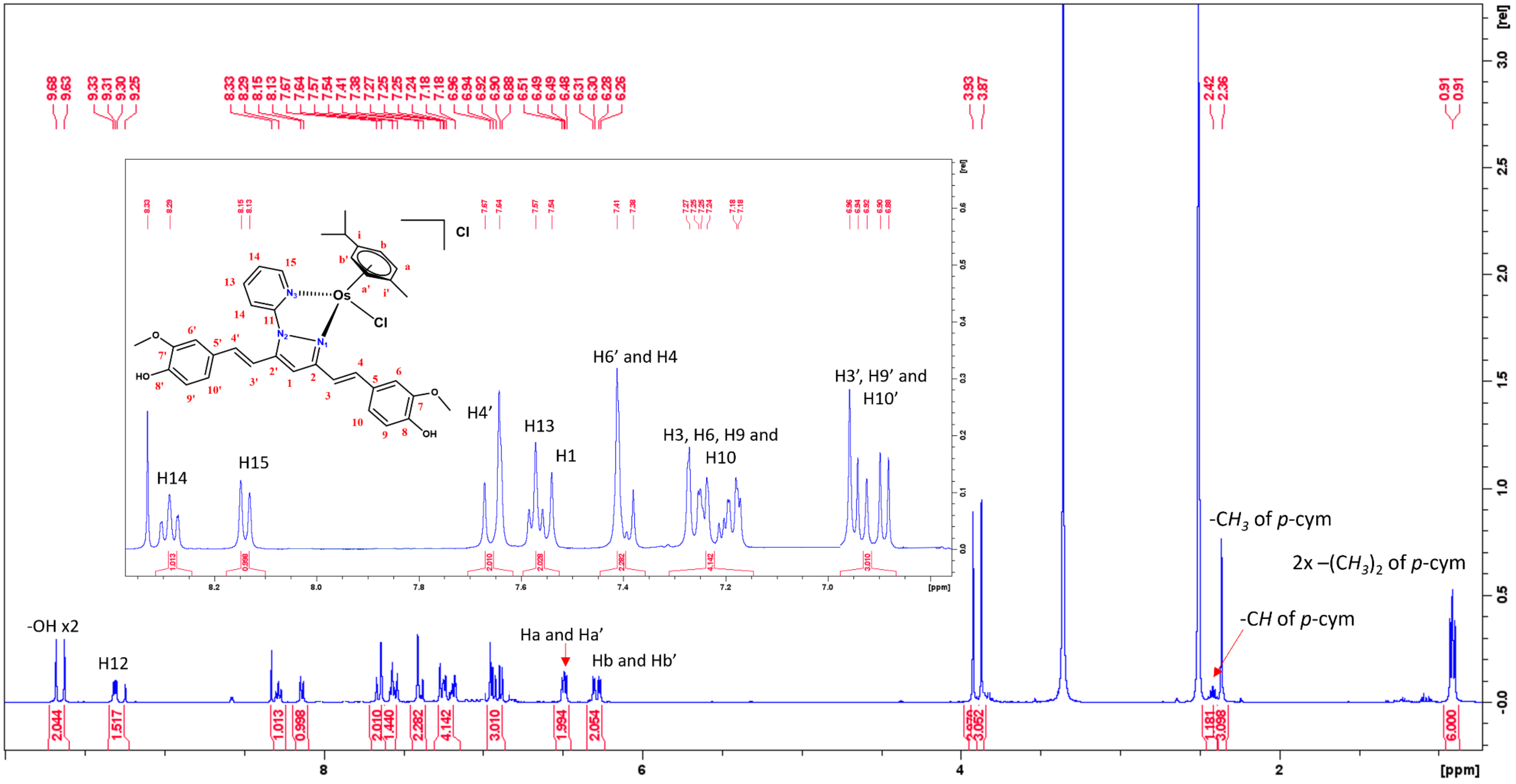

Figure S11. ^{1}H-NMR of **[2]Cl** in DMSO-d_6

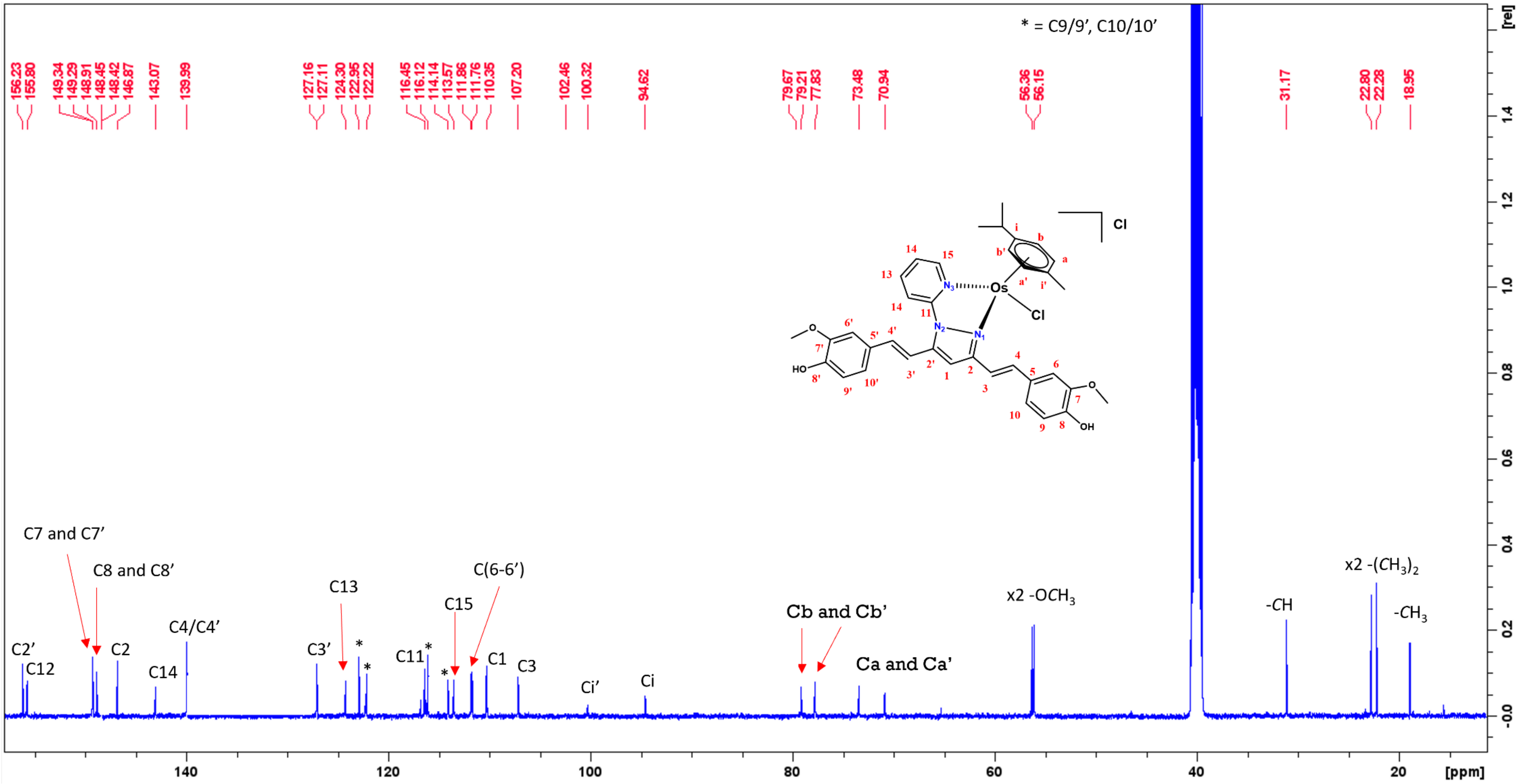

Figure S12. ^{13}C-NMR of **[2]Cl** in DMSO-d_6

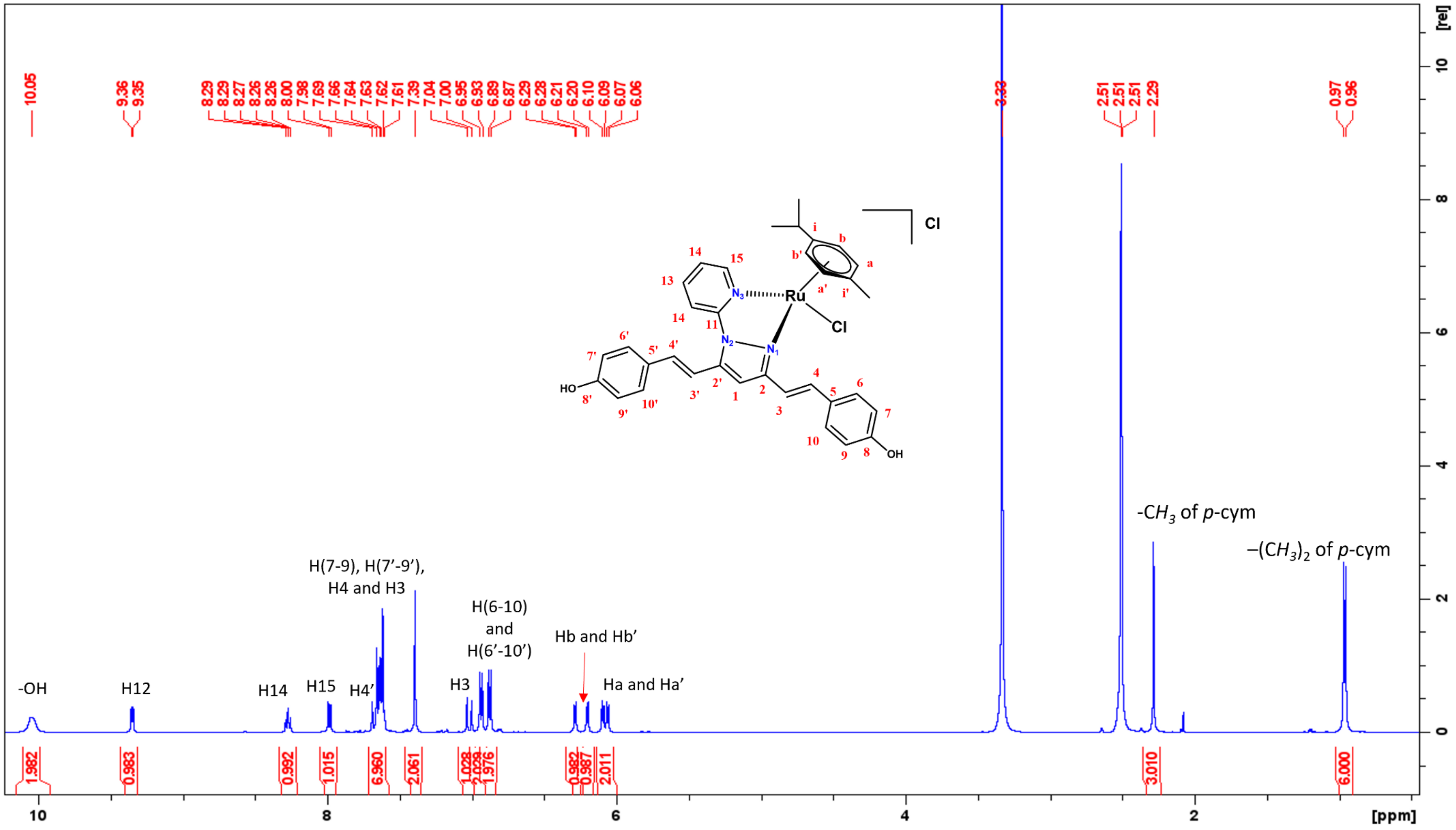

Figure S13. ^{1}H-NMR of **[3]Cl** in DMSO-d_6

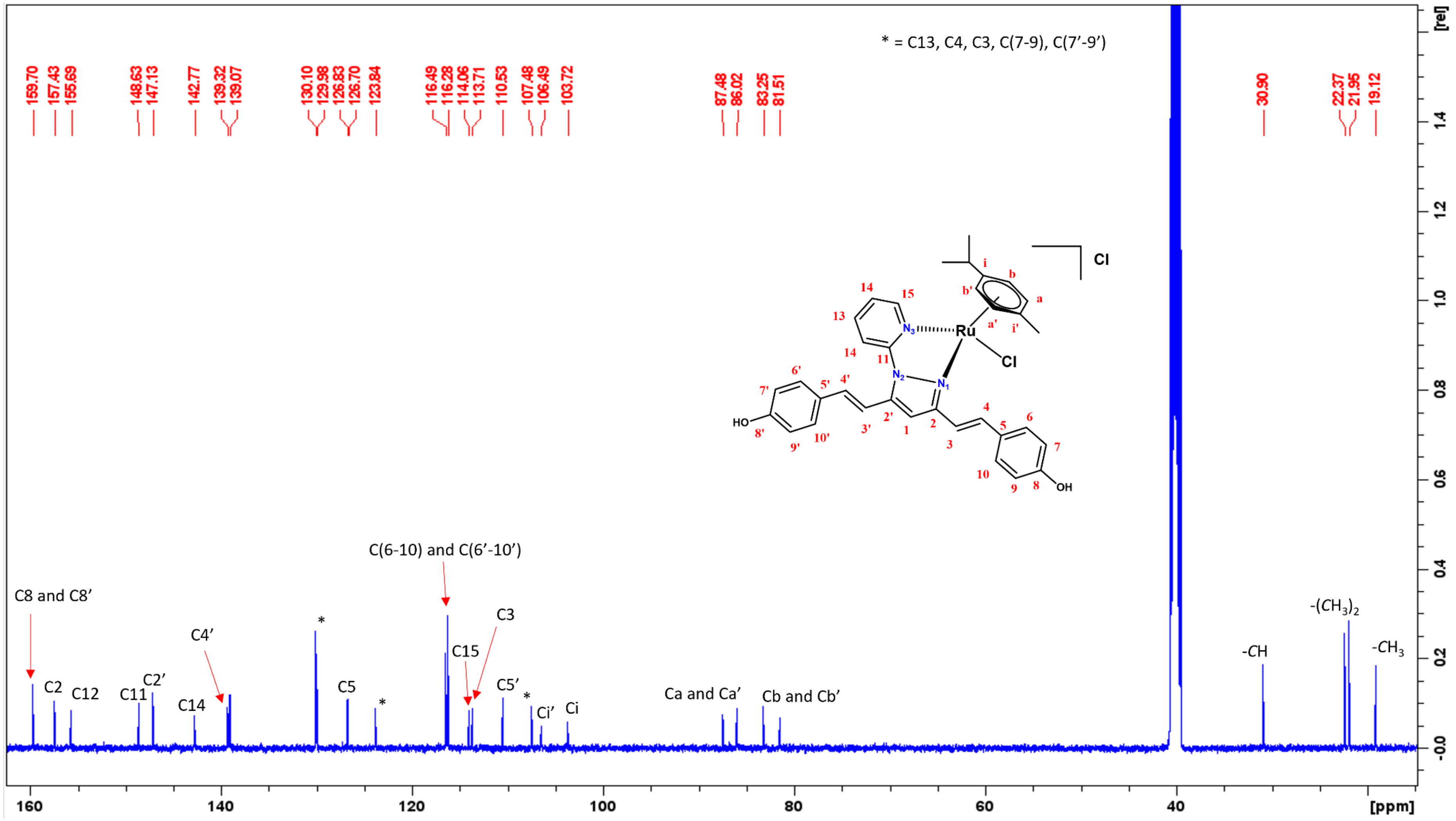

Figure S14. ^{13}C-NMR of **[3]Cl** in DMSO-d_6

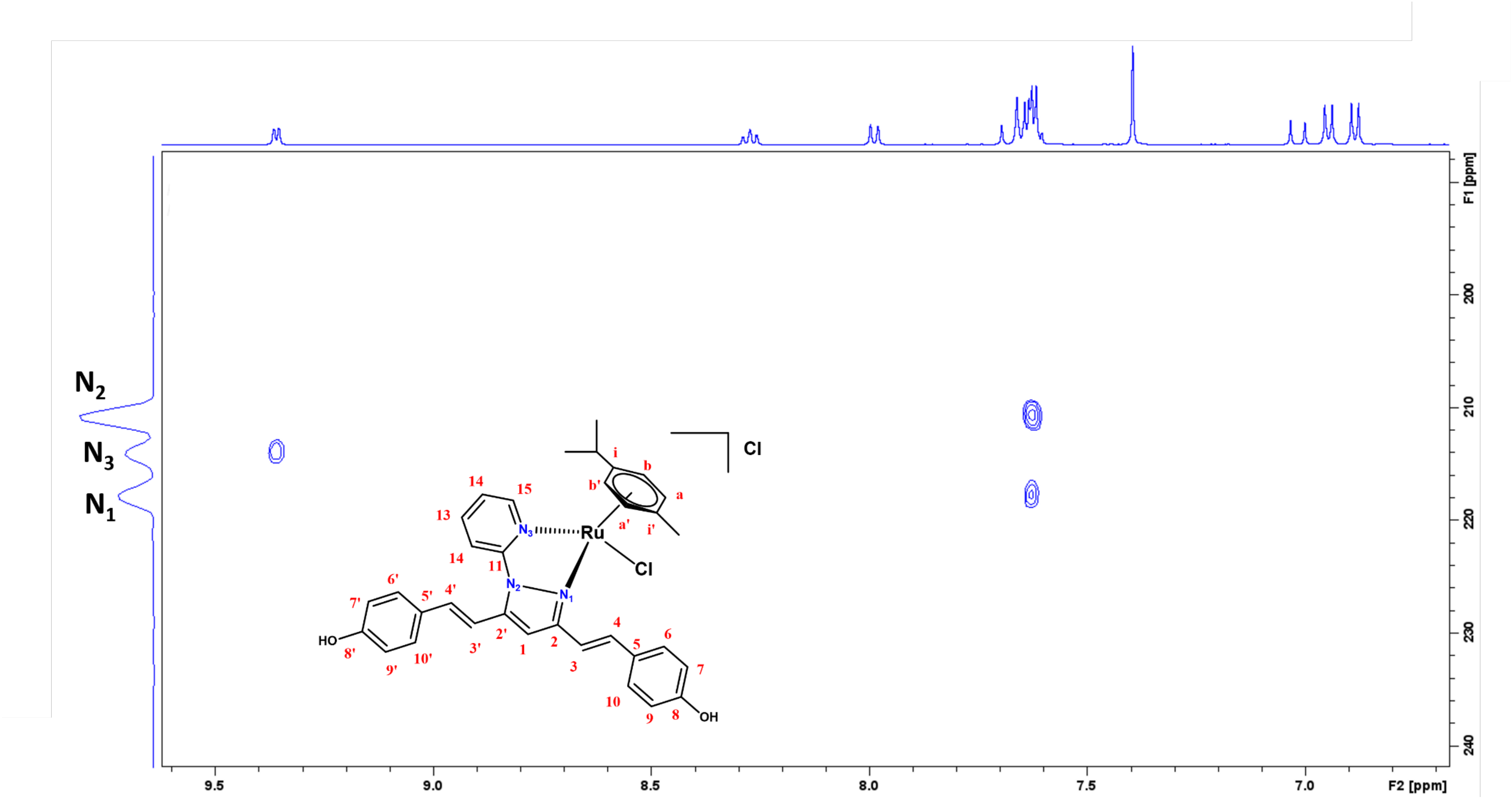

Figure S15. {^{1}H-^{15}N}-HMBC NMR of **[3]Cl** in DMSO-d_6

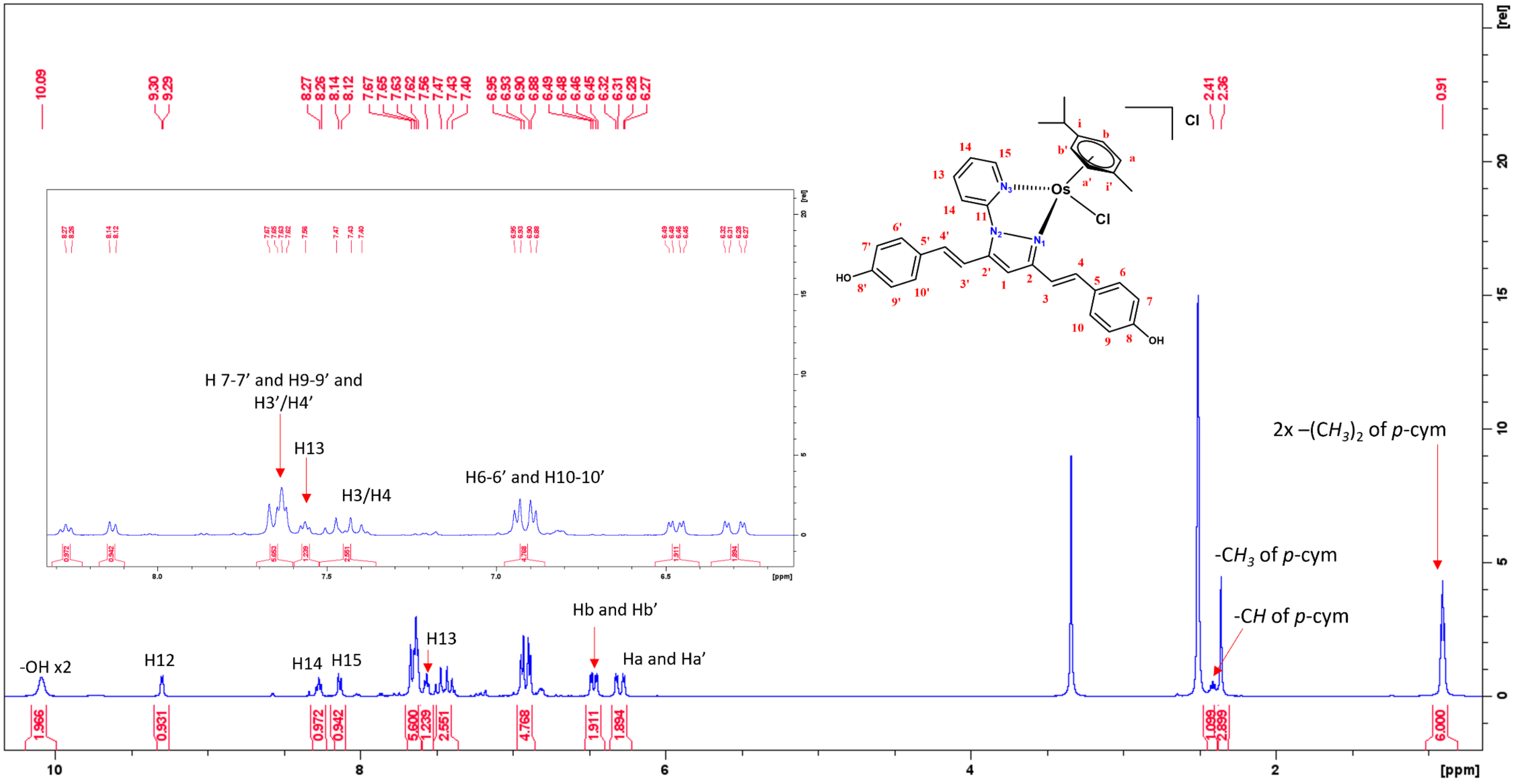

Figure S16. ^{1}H- NMR of **[4]Cl** in DMSO-d_6

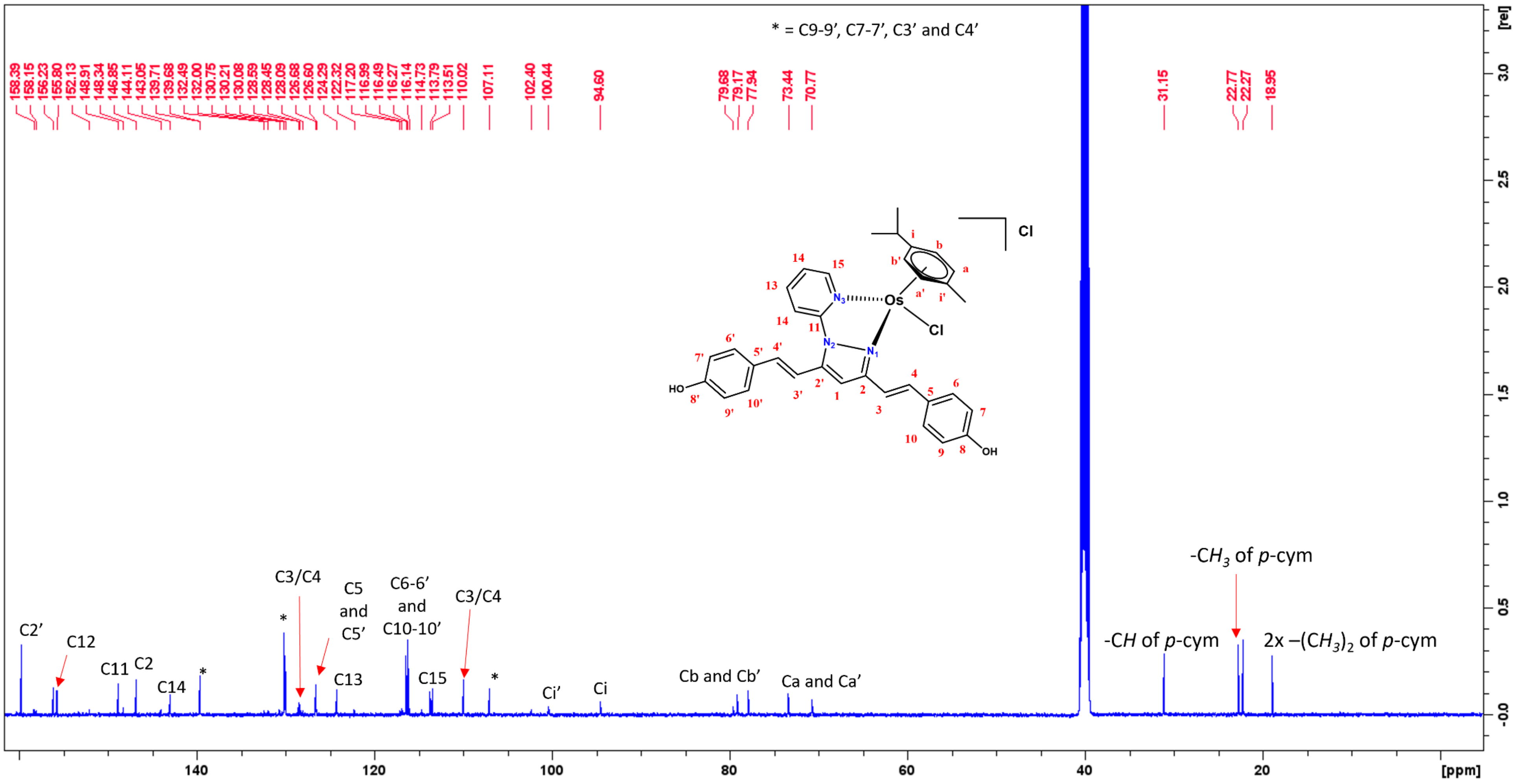

Figure S17. ^{13}C- NMR of **[4]Cl** in DMSO-d_6

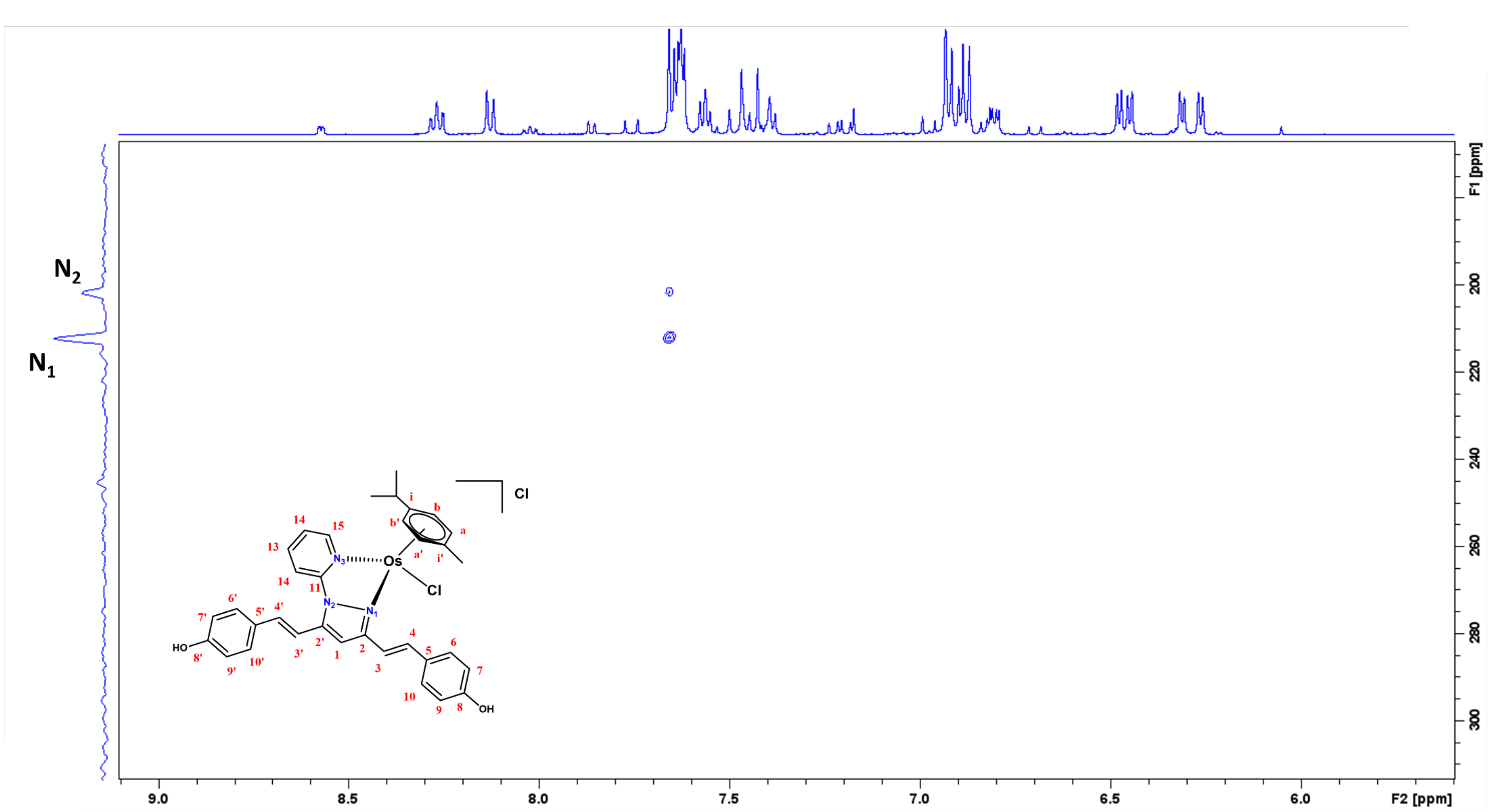

Figure S18. {^{1}H-^{15}N}-HMBC NMR of **[4]Cl** in DMSO-d_6

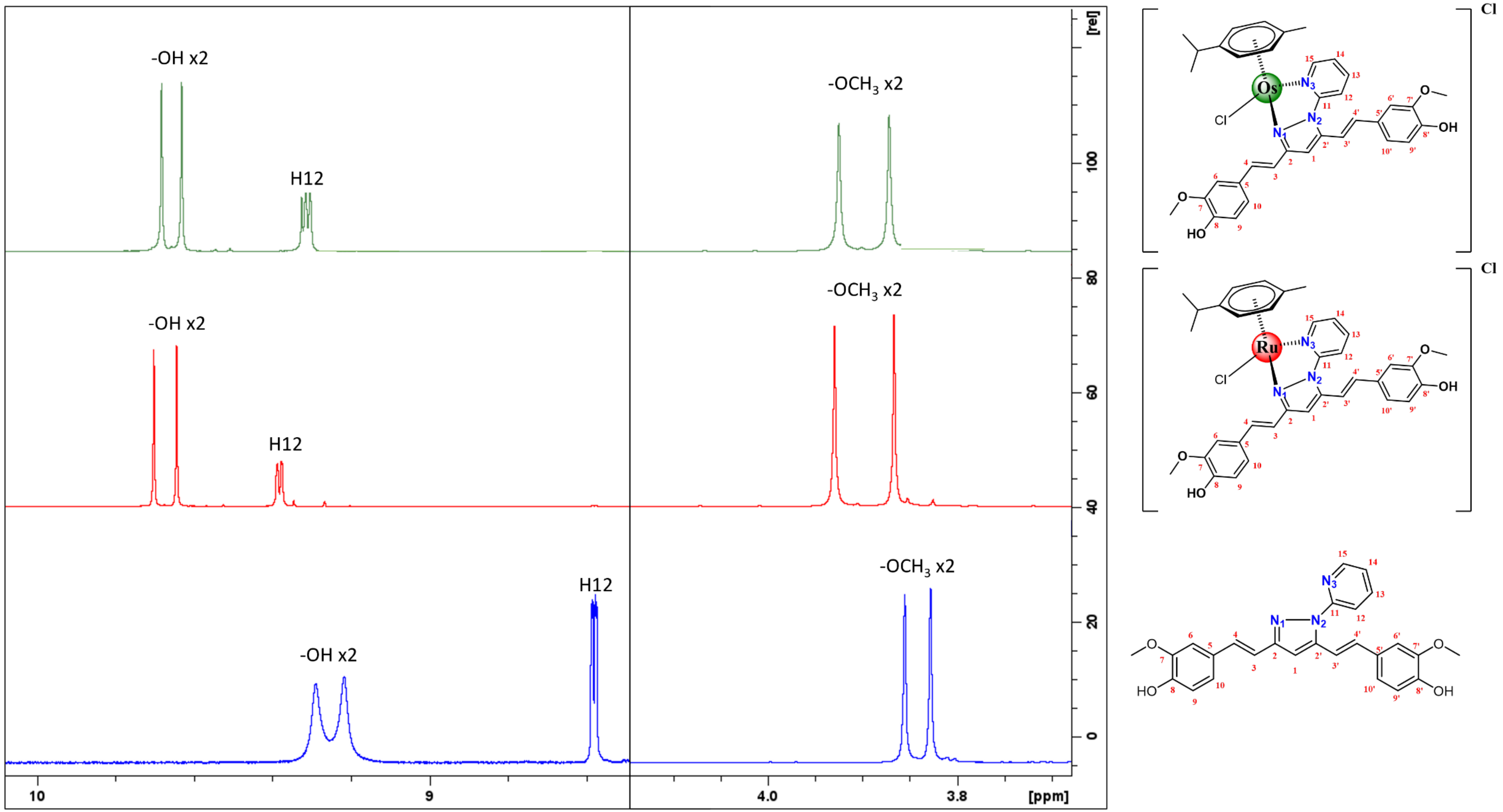

Figure S19. Comparison of ^{1}H-NMR spectra of ligand **HZPcurc** and complexes **[1]Cl**, **[3]Cl** in DMSO-d_6

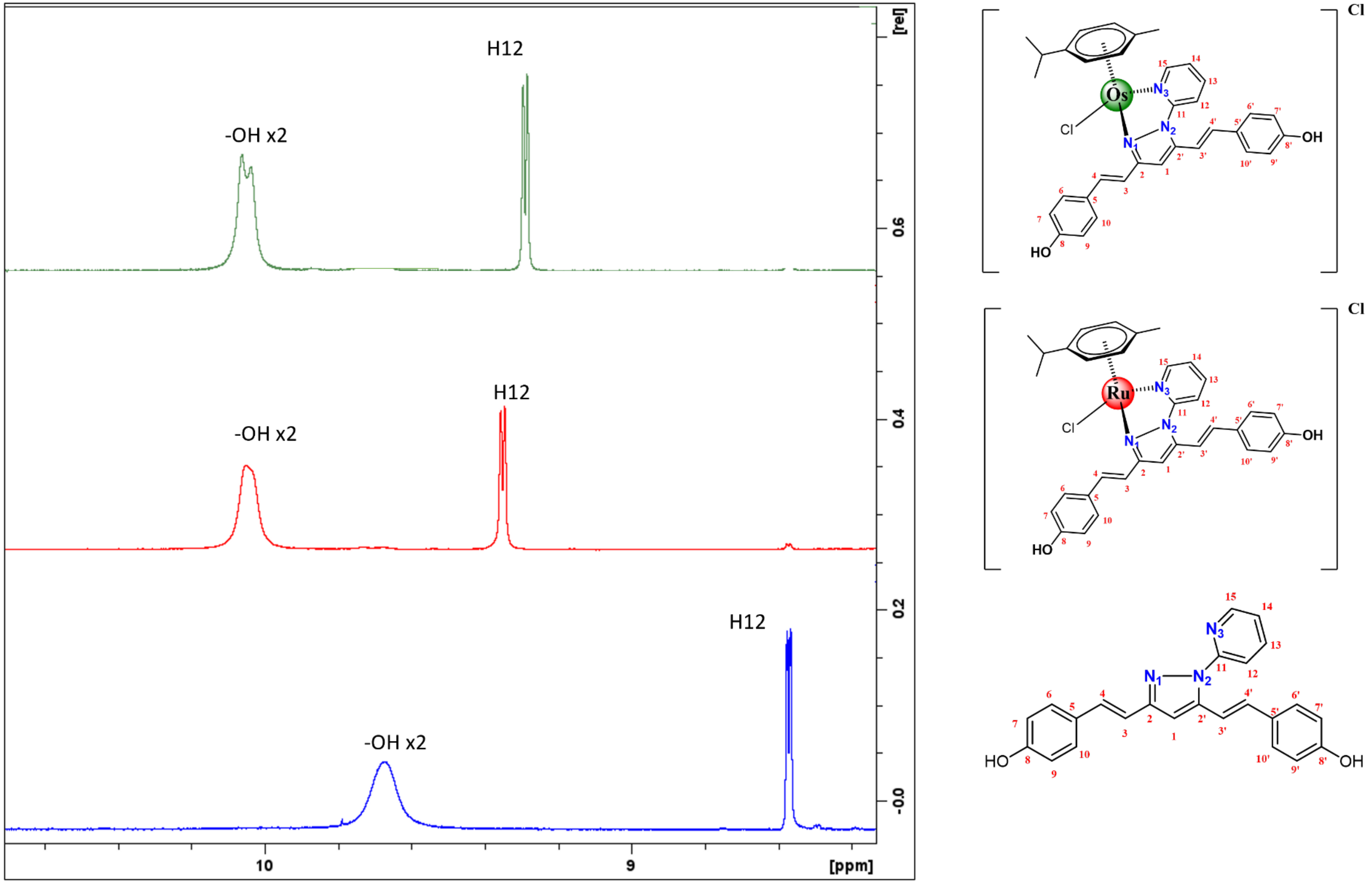

Figure S20. Comparison of ^{1}H-NMR spectra of ligand **HZPbdcurc** and complexes **[2]Cl**, **[4]Cl** in DMSO-d_6

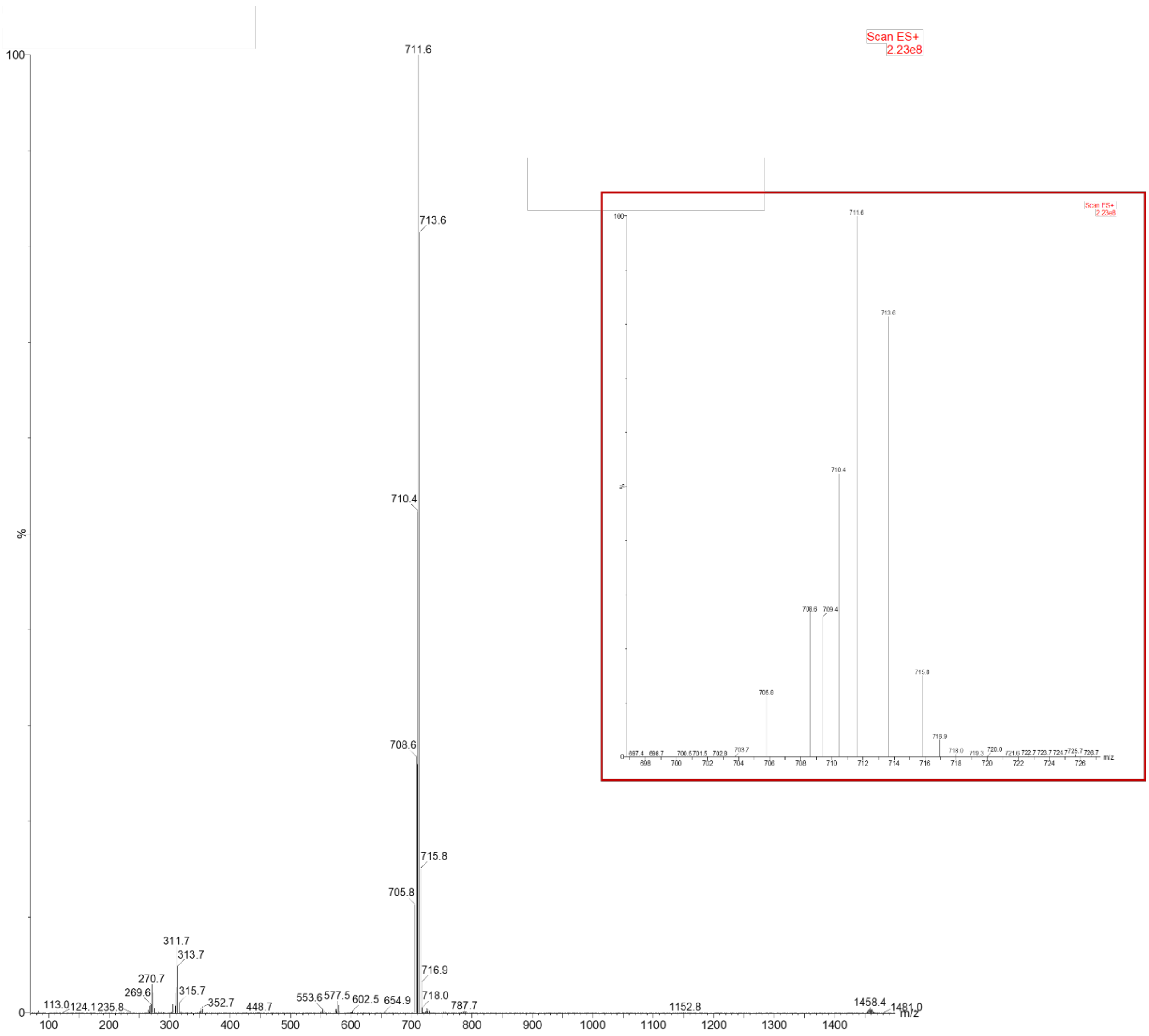

Figure S21. ESI-MS of **[1]⁺** in CH_3CN ([M-35] at 712 m/z)

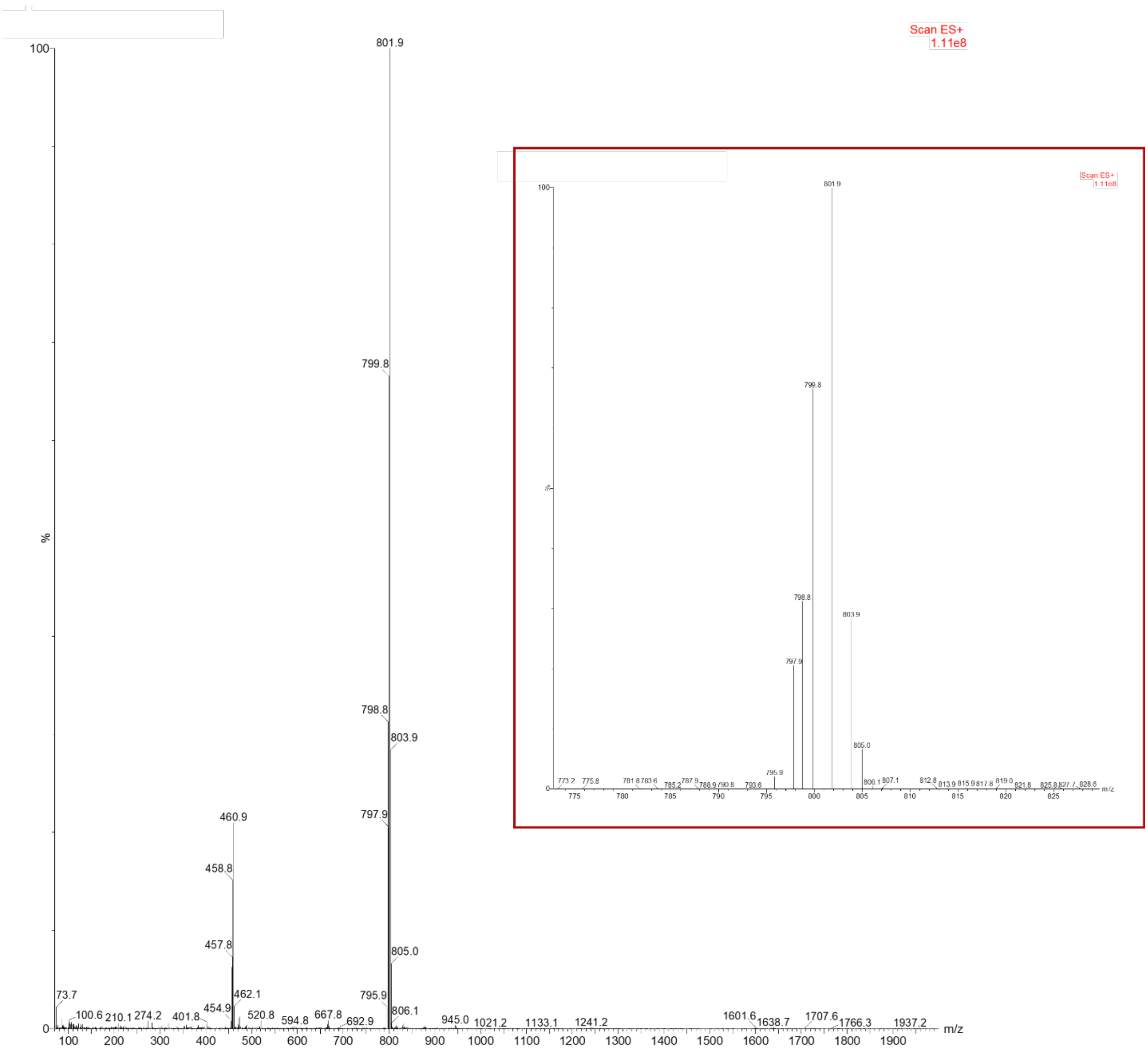

Figure S22. ESI-MS of **[2]**$^+$ in CH_3CN ([M-35] at 802 m/z)

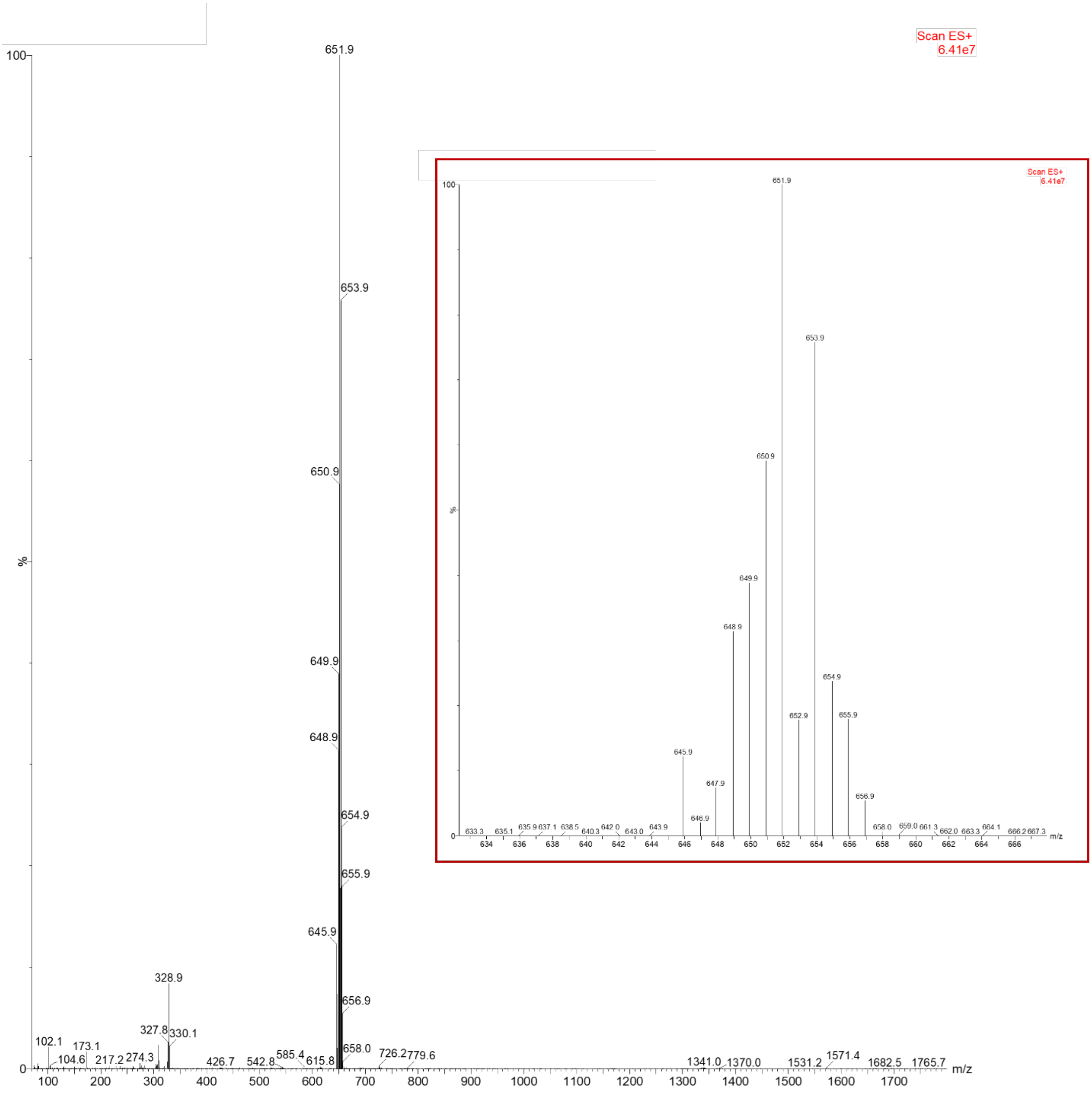

Figure S23. ESI-MS of **[3]⁺** in CH_3OH ([M-35] at 652 m/z)

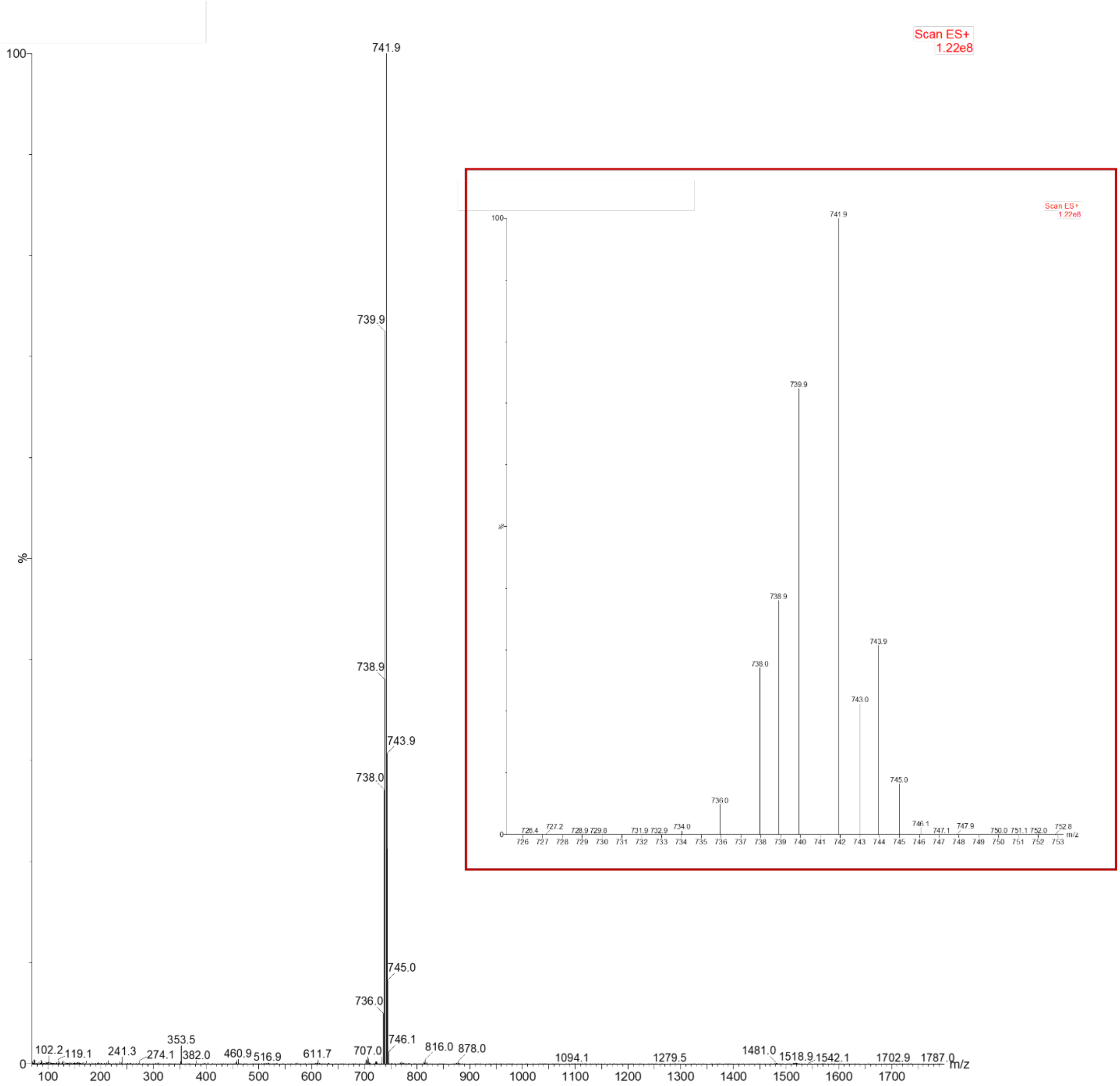

Figure S24. ESI-MS of **[4]⁺** in CH_3OH ([M-35] at 742 m/z)

Crystallography

Table S1. Selected structural parameters of **[1]Cl** and **[3]Cl**.

Bond Lengths in Å for **[1]Cl**.

Atom	Atom	Length/Å
Ru1	Cl1	2.3973(18)
Ru1	N1	2.068(7)
Ru1	N3	2.099(5)
Ru1	C27	2.233(8)
Ru1	C28	2.194(6)
Ru1	C29	2.177(7)
Ru1	C30	2.189(8)
Ru1	C31	2.162(8)
Ru1	C32	2.206(10)
O1	C13	1.378(9)
O1	C17	1.415(11)
O2	C14	1.377(8)
O3	C22	1.345(13)
O3	C26	1.423(13)
O4	C23	1.362(10)
N1	N2	1.364(7)

Atom	Atom	Length/Å
N1	C1	1.321(10)
N2	C3	1.388(11)
N2	C4	1.418(10)
N3	C4	1.338(11)
N3	C8	1.332(10)
C1	C2	1.393(11)
C1	C9	1.461(9)
C2	C3	1.381(10)
C3	C18	1.452(10)
C4	C5	1.387(9)
C5	C6	1.370(12)
C6	C7	1.403(14)
C7	C8	1.392(9)
C9	C10	1.346(11)
C18	C19	1.345(13)

Bond Angles in ° for **[1]Cl**.

Atom	Atom	Atom	Angle/°
N1	Ru1	Cl1	85.46(17)
N1	Ru1	N3	75.0(2)
N3	Ru1	Cl1	86.37(17)
N3	Ru1	C27	125.7(3)
N3	Ru1	C28	162.9(3)
N3	Ru1	C29	152.1(3)
N3	Ru1	C30	115.6(3)
N3	Ru1	C31	94.0(3)
N3	Ru1	C32	98.9(3)
C13	O1	C17	117.8(6)
C22	O3	C26	116.9(8)
N2	N1	Ru1	116.1(5)
C1	N1	Ru1	135.3(5)
C1	N1	N2	108.2(6)
N1	N2	C3	109.3(6)
N1	N2	C4	115.7(6)
C3	N2	C4	134.5(6)
C4	N3	Ru1	118.2(5)
C8	N3	Ru1	122.0(6)
C8	N3	C4	119.8(6)
N1	C1	C2	109.2(6)
N1	C1	C9	120.3(7)
C2	C1	C9	130.5(8)
C3	C2	C1	107.5(8)
N2	C3	C18	127.1(7)
C2	C3	N2	105.7(7)
C2	C3	C18	127.2(8)
N3	C4	N2	113.0(5)
N3	C4	C5	122.7(7)
C5	C4	N2	124.3(8)
C6	C5	C4	117.6(8)
C5	C6	C7	120.6(7)

Atom	Atom	Atom	Angle/°
C8	C7	C6	117.8(8)
N3	C8	C7	121.5(8)
C10	C9	C1	123.3(8)
C9	C10	C11	123.7(7)
C12	C11	C10	118.1(7)
C16	C11	C10	122.7(7)
C16	C11	C12	119.2(6)
C13	C12	C11	120.2(7)
O1	C13	C12	123.4(7)
C14	C13	O1	116.9(6)
C14	C13	C12	119.7(7)
O2	C14	C15	120.8(7)
C13	C14	O2	118.1(7)
C13	C14	C15	121.0(6)
C14	C15	C16	118.7(7)
C11	C16	C15	121.1(7)
C19	C18	C3	120.9(7)
C18	C19	C20	127.5(8)
C21	C20	C19	117.0(8)
C21	C20	C25	119.1(8)
C25	C20	C19	123.8(9)
C22	C21	C20	122.5(9)
O3	C22	C21	127.4(9)
O3	C22	C23	114.6(8)
C21	C22	C23	117.9(10)
O4	C23	C22	120.0(10)
O4	C23	C24	120.4(9)
C24	C23	C22	119.6(8)
C23	C24	C25	122.0(8)
C24	C25	C20	118.9(9)

Bond Lengths in Å for **[3]Cl**.

Atom	Atom	Length/Å
Ru1	Cl1	2.396(2)
Ru1	N1	2.070(8)
Ru1	N3	2.104(7)
Ru1	C25	2.236(11)
Ru1	C26	2.212(9)
Ru1	C27	2.176(8)
Ru1	C28	2.228(9)
Ru1	C29	2.172(10)
Ru1	C30	2.195(10)
O1	C14	1.370(13)
O2	C22	1.353(13)
N1	N2	1.387(11)
N1	C1	1.352(11)
N2	C3	1.392(12)
N2	C4	1.380(12)
N3	C4	1.338(11)
N3	C8	1.349(12)
C10	C11	1.458(13)
C17	C18	1.321(13)

Atom	Atom	Length/Å
Ru2	Cl2	2.394(2)
Ru2	N4	2.081(7)
Ru2	N6	2.078(8)
Ru2	C59	2.223(11)
Ru2	C60	2.211(10)
Ru2	C61	2.166(9)
Ru2	C62	2.226(10)
Ru2	C63	2.171(10)
Ru2	C64	2.218(10)
O3	C48	1.380(15)
O4	C56	1.341(13)
N4	N5	1.363(11)
N4	C35	1.317(11)
N5	C37	1.385(12)
N5	C38	1.415(12)
N6	C38	1.323(12)
N6	C42	1.385(12)
C43	C44	1.327(13)
C51	C52	1.344(14)

Bond Angles in ° for **[3]Cl**.

Atom	Atom	Atom	Angle/°
N1	Ru1	Cl1	85.5(2)
N1	Ru1	N3	75.5(3)
N3	Ru1	Cl1	85.4(2)
N2	N1	Ru1	115.6(6)
C1	N1	Ru1	137.1(6)
C1	N1	N2	106.9(7)
N1	N2	C3	110.3(8)
C4	N2	N1	116.6(7)
C4	N2	C3	133.0(8)
C4	N3	Ru1	117.5(6)
C4	N3	C8	119.5(8)
C8	N3	Ru1	122.9(6)
N1	C1	C2	108.8(8)
N1	C1	C9	121.6(8)
C2	C1	C9	129.6(8)
C3	C2	C1	108.3(8)
N2	C3	C17	126.9(9)
C2	C3	N2	105.6(8)
C2	C3	C17	127.5(9)
N3	C4	N2	114.3(8)
N3	C4	C5	121.3(8)
C5	C4	N2	124.3(8)
N3	C8	C7	122.1(9)
C10	C9	C1	124.7(9)
C9	C10	C11	126.2(9)
C18	C17	C3	122.4(9)
C17	C18	C19	127.6(9)
O2	C22	C21	115.6(10)
O2	C22	C23	124.7(10)
N4	Ru2	Cl2	84.8(2)
N6	Ru2	Cl2	85.9(2)
N6	Ru2	N4	75.0(3)

Atom	Atom	Atom	Angle/°
N5	N4	Ru2	115.9(6)
C35	N4	Ru2	136.9(7)
C35	N4	N5	106.9(8)
N4	N5	C37	110.9(8)
N4	N5	C38	116.2(8)
C37	N5	C38	132.8(8)
C38	N6	Ru2	119.7(6)
C38	N6	C42	116.4(8)
C42	N6	Ru2	123.6(6)
N4	C35	C36	110.1(8)
N4	C35	C43	121.8(8)
C36	C35	C43	128.0(8)
C37	C36	C35	106.6(8)
N5	C37	C51	124.4(9)
C36	C37	N5	105.5(8)
C36	C37	C51	130.0(9)
N6	C38	N5	112.5(8)
N6	C38	C39	124.1(9)
C39	C38	N5	123.3(8)
C40	C39	C38	117.9(9)
C39	C40	C41	119.8(11)
C42	C41	C40	118.1(10)
C41	C42	N6	123.7(9)
C44	C43	C35	124.2(9)
C43	C44	C45	125.3(9)
O3	C48	C49	121.0(11)
C47	C48	O3	117.3(9)
C52	C51	C37	120.4(9)
C51	C52	C53	127.6(9)
O4	C56	C55	118.6(9)
O4	C56	C57	122.4(9)

Table S2. Crystal data and structure refinement for complexes **[1]Cl** and **[3]Cl**.

**Compound**	**[1]Cl**	**[3]Cl**
Formula	$C_{39}H_{43}Cl_2N_3O_{5.5}Ru$	$C_{34}H_{33}Cl_2N_3O_2Ru$
$D_{calc.}$/ g cm^{-3}	1.421	1.452
μ/mm^{-1}	5.016	5.874
Formula Weight	813.73	687.60
Colour	clear intense orange	clear intense orange
Shape	prism-shaped	needle-shaped
Size/mm^3	0.09×0.06×0.04	0.18×0.02×0.01
T/K	140.00(10)	140.00(10)
Crystal System	triclinic	orthorhombic
Space Group	$P\bar{1}$	$Pca2_1$
a/Å	10.3696(7)	29.8824(5)
b/Å	12.8494(9)	7.51550(10)
c/Å	15.6940(11)	28.0072(5)
α/°	70.462(6)	90
β/°	76.251(6)	90
γ/°	79.517(6)	90
V/Å^3	1902.2(2)	6289.89(18)
Z	2	8
Z'	1	2
Wavelength/Å	1.54184	1.54184
Radiation type	Cu$K\alpha$	Cu$K\alpha$
Θ_{min}/°	3.673	2.958
Θ_{max}/°	72.805	74.693
Measured Refl's.	13573	38623
Indep't Refl's	7305	11119
Refl's I≥2σ(I)	5930	9369
R_{int}	0.0427	0.0409
Parameters	432	768
Restraints	694	1
Largest Peak/e Å^{-3}	1.887	5.952
Deepest Hole/e Å^{-3}	-1.358	-1.047
GooF	1.052	1.020
wR_2 (all data)	0.2239	0.1572
wR_2	0.2135	0.1490
R_1 (all data)	0.1015	0.0723
R_1	0.0858	0.0603
CCDC number	2378944	2378945

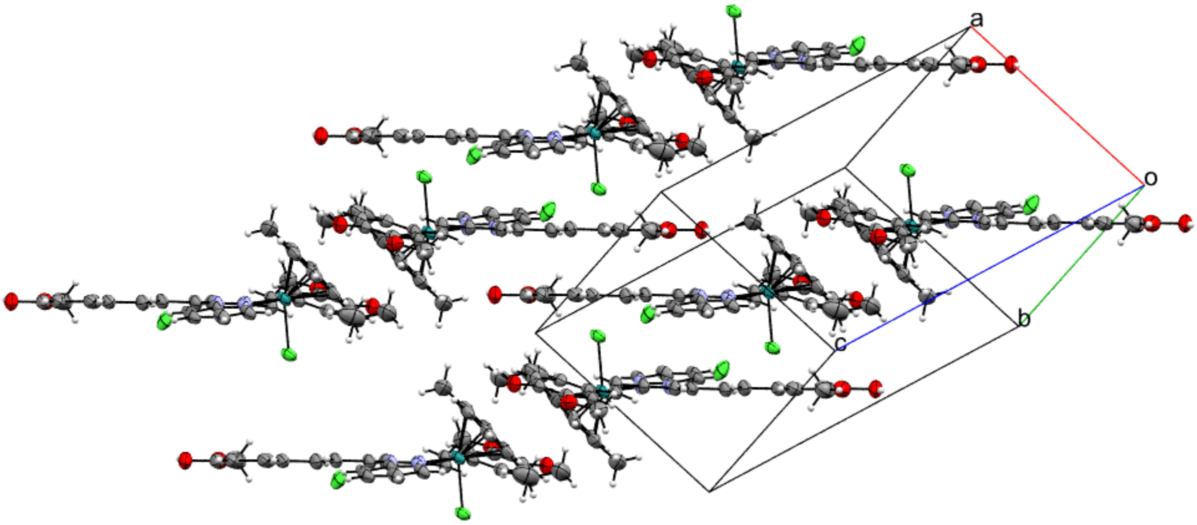

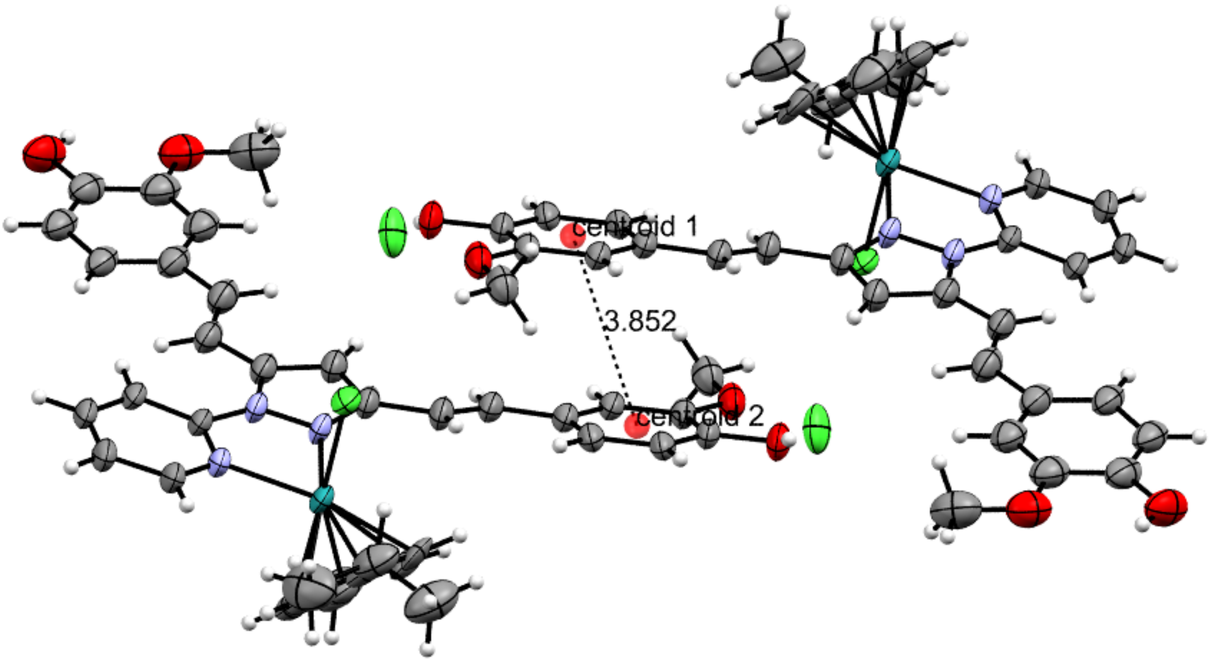

Figure S25. Top: crystal packing of **[1]Cl**. Bottom: π-stacking interaction in **[1]Cl**.

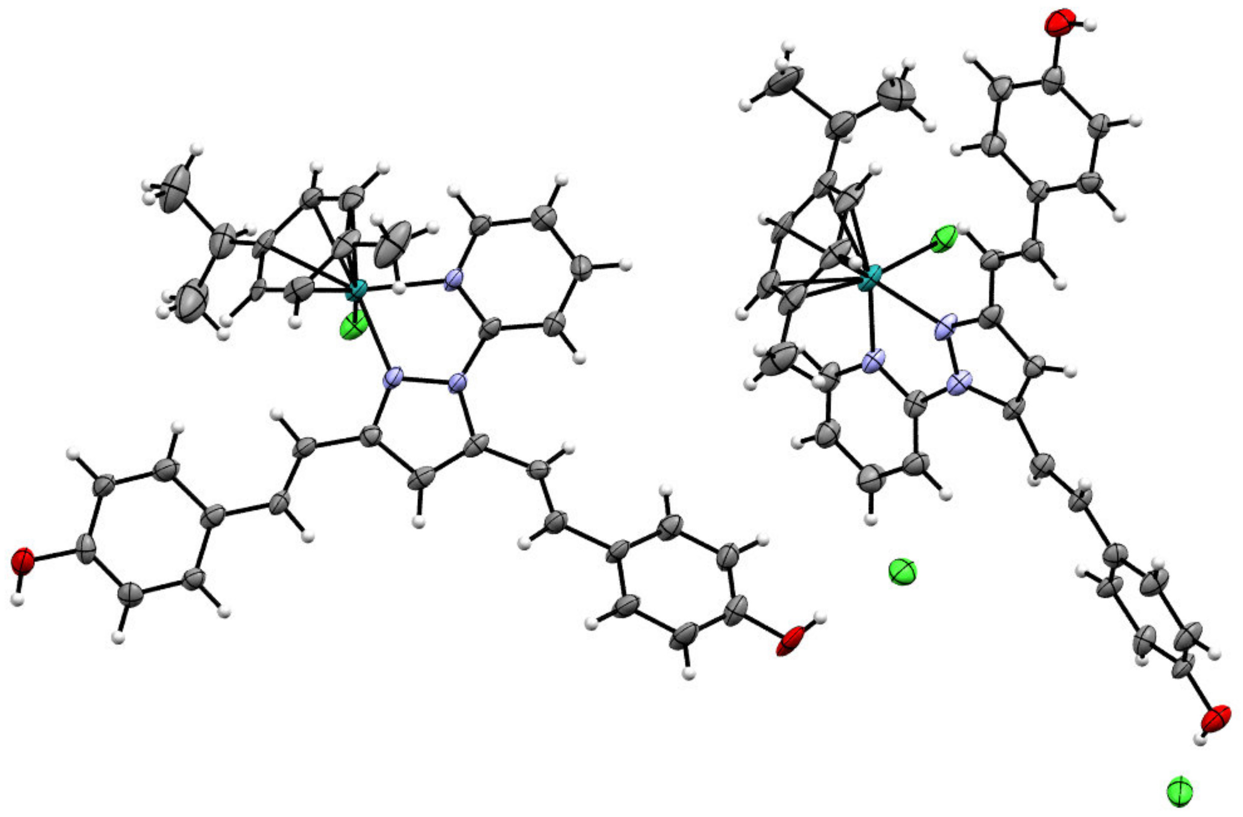

Figure S26. Asymmetric unit of **[3]Cl**.

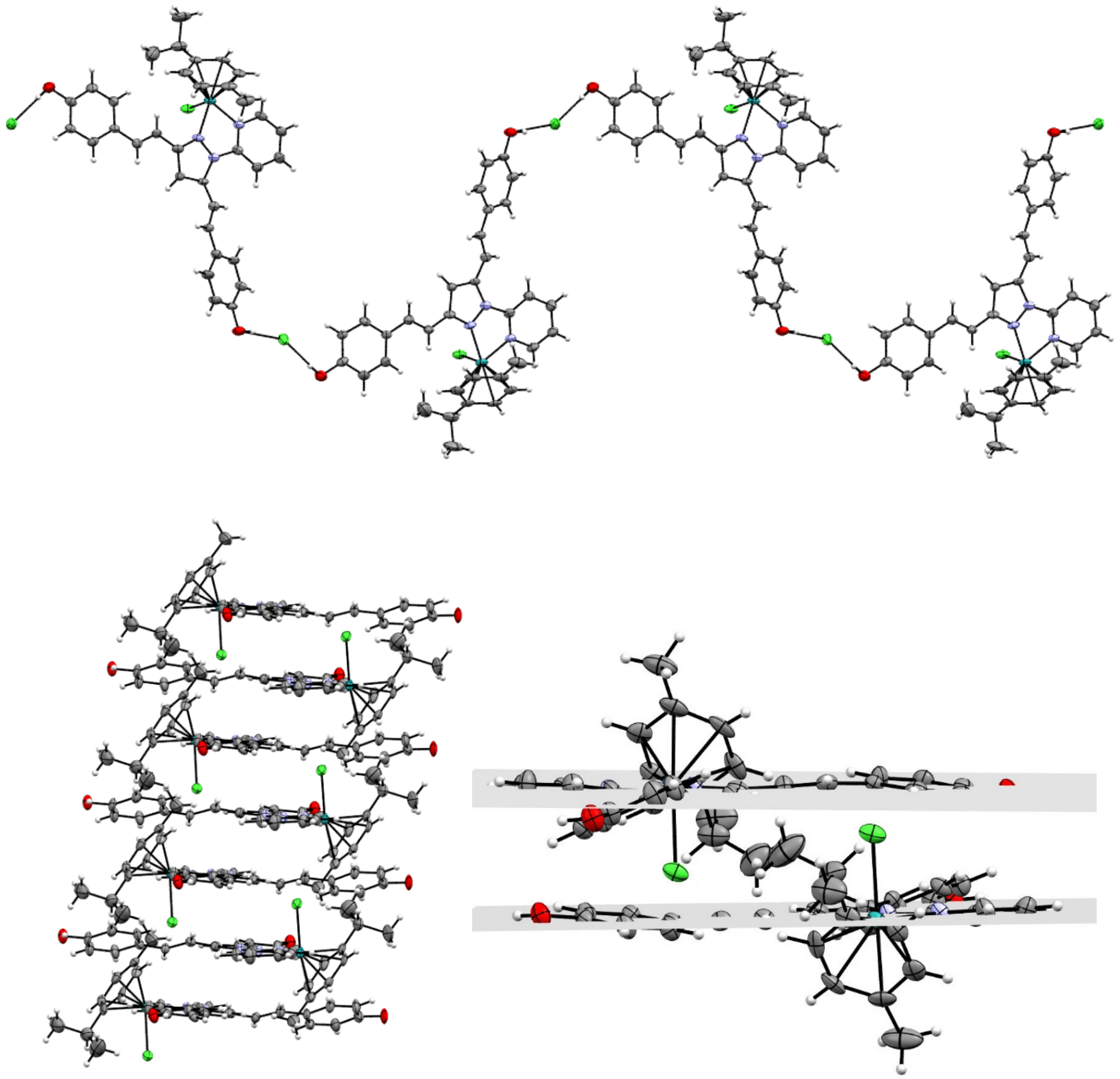

Figure S27. Top: bifurcated hydrogen bonds in **[3]Cl**. Bottom: packing in **[3]Cl** involving intercalation of HZPbdcurc ligands.

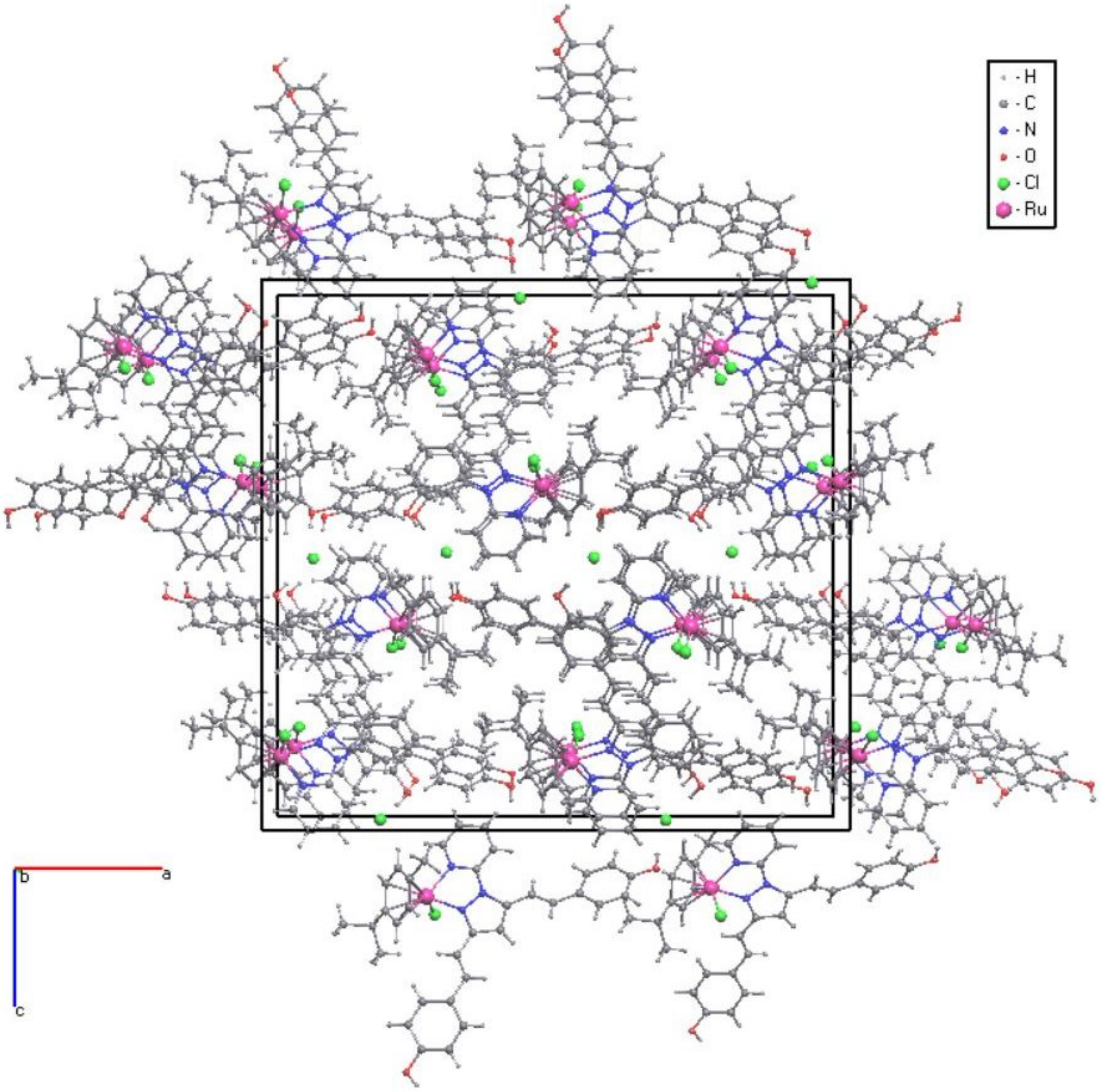

Figure S28. Crystal packing of **[3]Cl**.

Theoretical studies

Coordinates of compounds optimized as XYZ files are available as supplementary material.

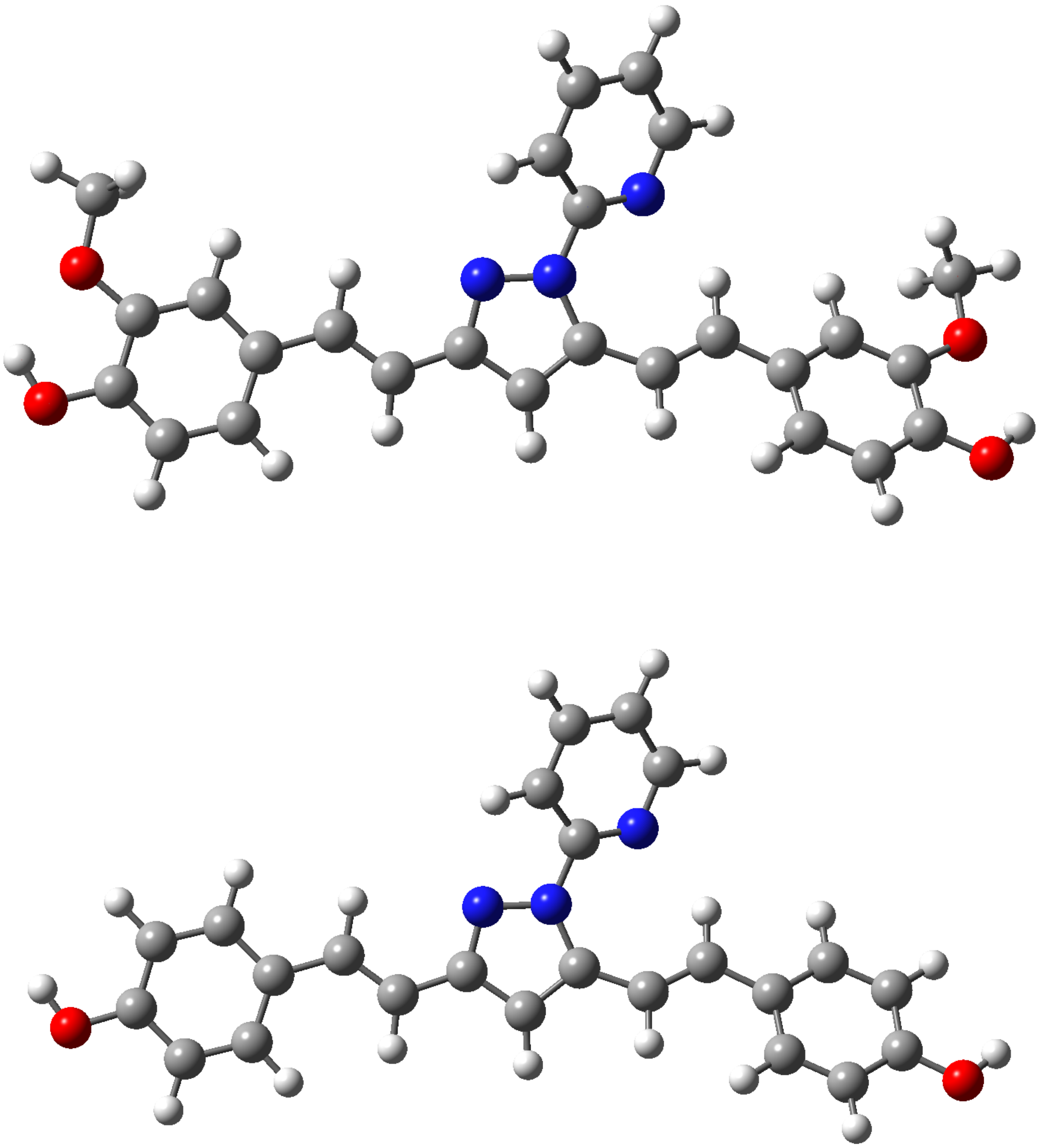

Figure S29. Optimized structures of **HZPcurc** (top) and **HZPbdcurc** (bottom).

Table S3. Selected calculated structural parameters of **HZPcurc** and **HZPbdcurc**.

**Bond distances (Å) and angles (º)**	**HZPcurc**	**HZPbdcurc**
N-N	1.363	1.363
C-N (pyrazolyl)	1.330, 1.395	1.330, 1.395
C-C (pyrazolyl)	1.386, 1.412	1.386, 1.412
C=C	1.345, 1.346	1.345, 1.346
N-C_{ortho}(py)	1.420	1.420
$C_{pyrazolyl}$-$C_{C=C}$	1.454, 1.456	1.454, 1.456
H-C=C	116.8, 120.5<br>117.6, 118.2	117.0, 120.5<br>117.6, 118.2
**Deviations from planarity (º)**		
Pyridine ring	31.8	30.8
Phenyl ring	35.3	31.2

HZPcurc

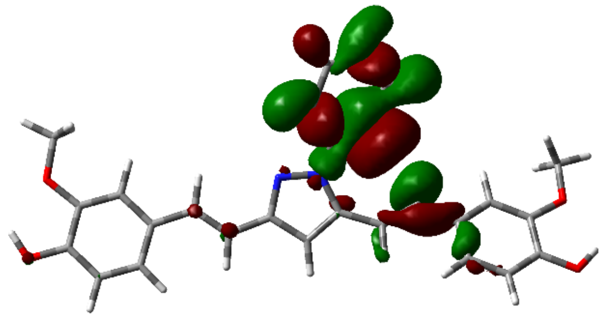

HOMO-7

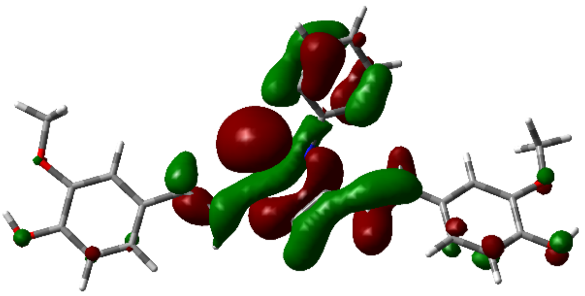

HOMO-8

HZPbdcurc

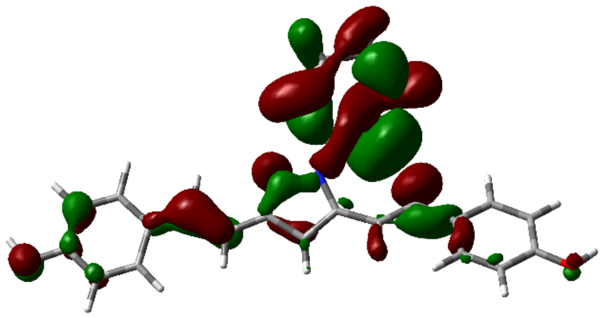

HOMO-6

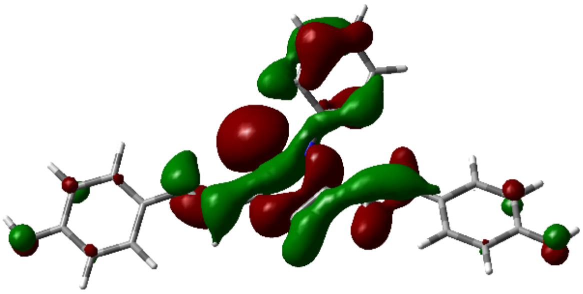

HOMO-8

Figure S30. Selected molecular orbitals of HZPcurc and HZPbdcurc.

Table S4. Selected structural parameters (bond distances, Å and angles, °) of complexes **[1]Cl-[4]Cl** and comparison with experimental data for **[1]Cl** and **[3]Cl** (red numbers).

Complex	[1]Cl	[2]Cl	[3]Cl	[4]Cl
M-Cl	2.422<br>2.397(2)	2.438	2.418<br>2.396(2)	2.433
M-N_{py}	2.110<br>2.099(5)	2.112	2.109<br>2.104(7)	2.109
M-N	2.080<br>2.068(7)	2.073	2.092<br>2.070(8)	2.085
M-C_{arene} (av.)	2.295<br>2.194	2.260	2.296<br>2.203	2.261
Cl-M-N_{py}	84.5<br>86.4(2)	83.1	84.0<br>85.4(2)	82.9
Cl-M-N	85.7<br>85.5(2)	84.3	85.3<br>85.5(2)	84.5
N-M-N_{py}	75.8<br>75.0(2)	75.4	76.0<br>75.5(3)	75.4

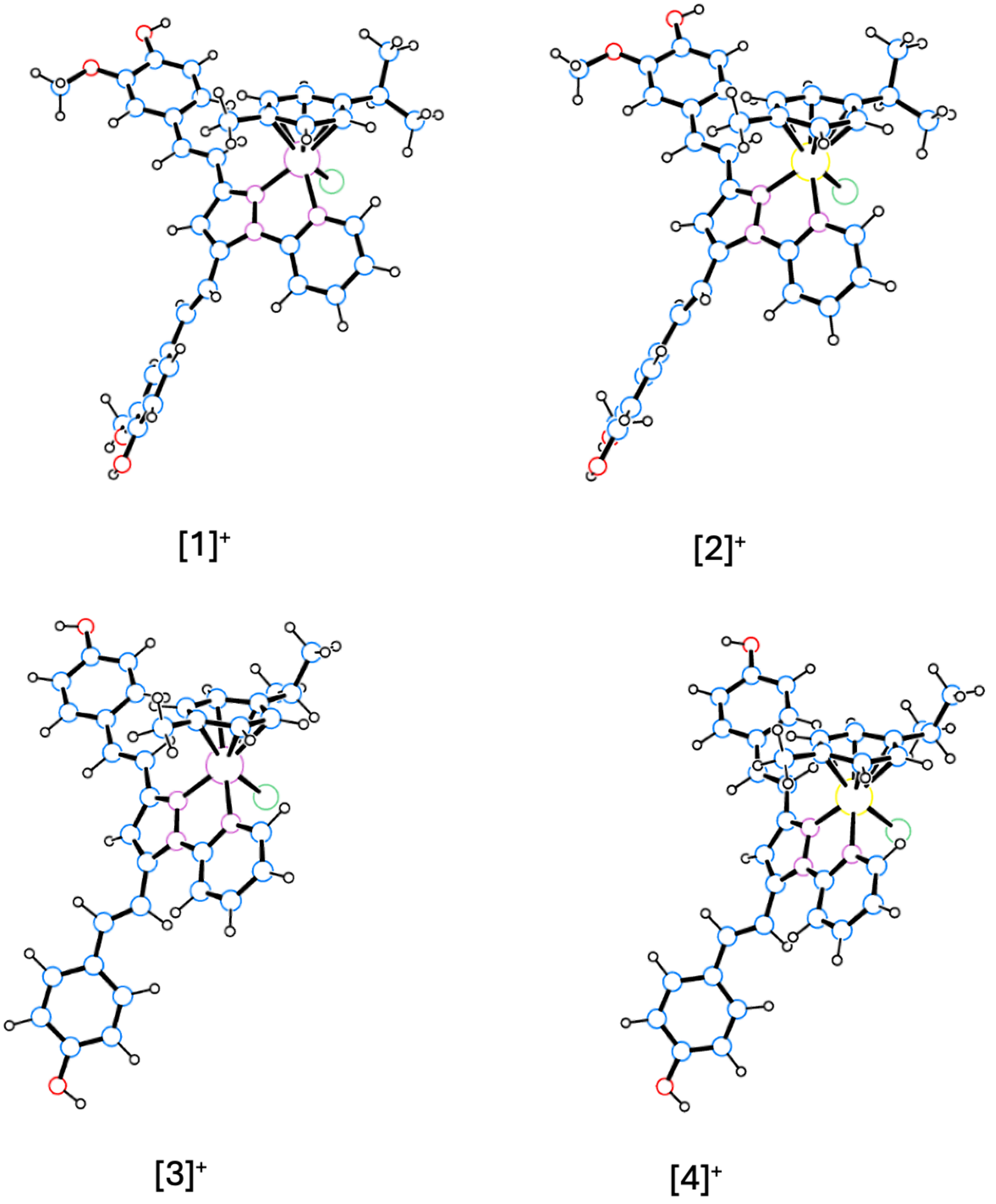

Figure S31. Optimized structures of complexes **[1]+-[4]+**.

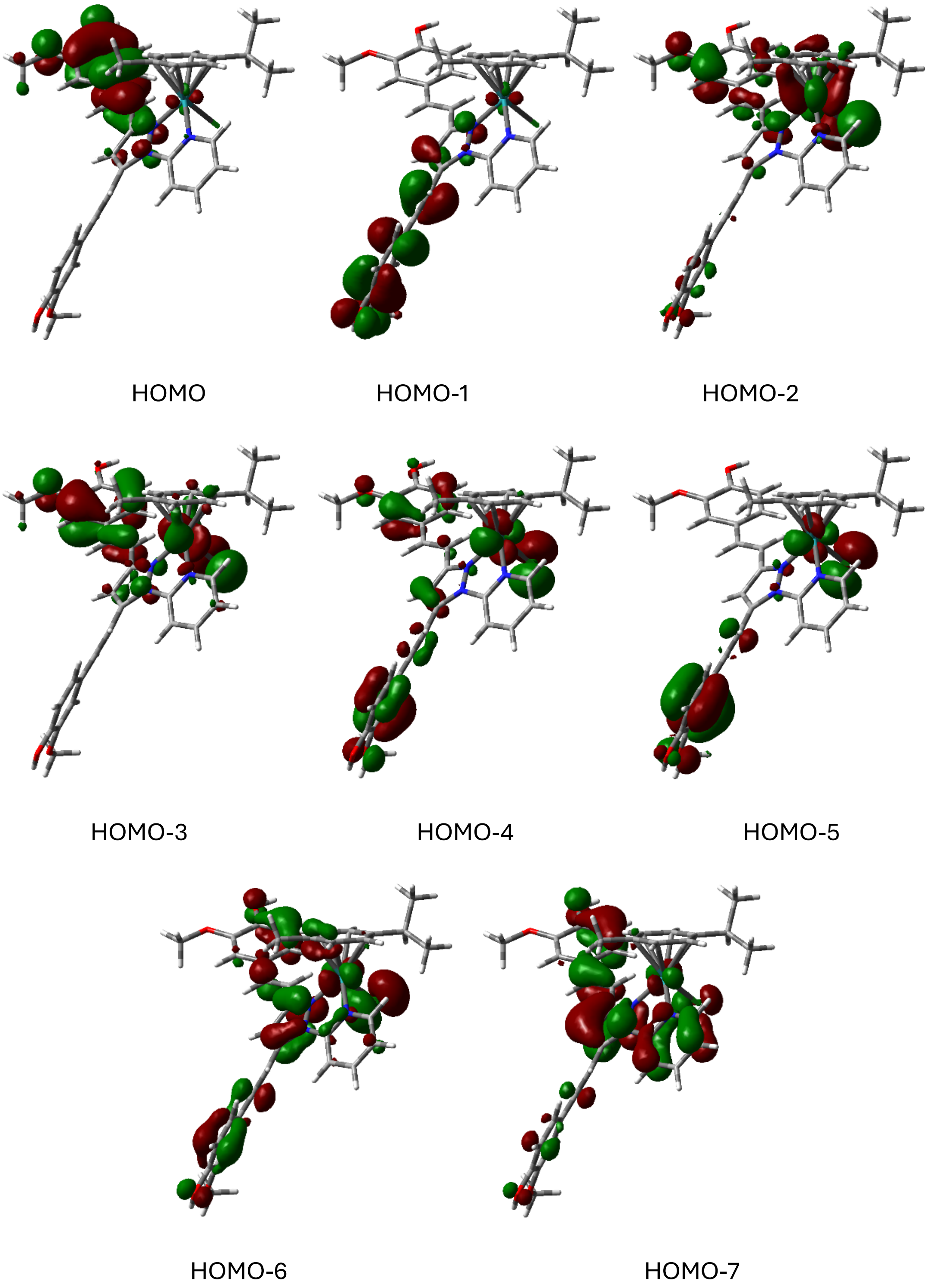

Figure S32. Selected molecular orbitals of **[1]Cl**.

Stability studies

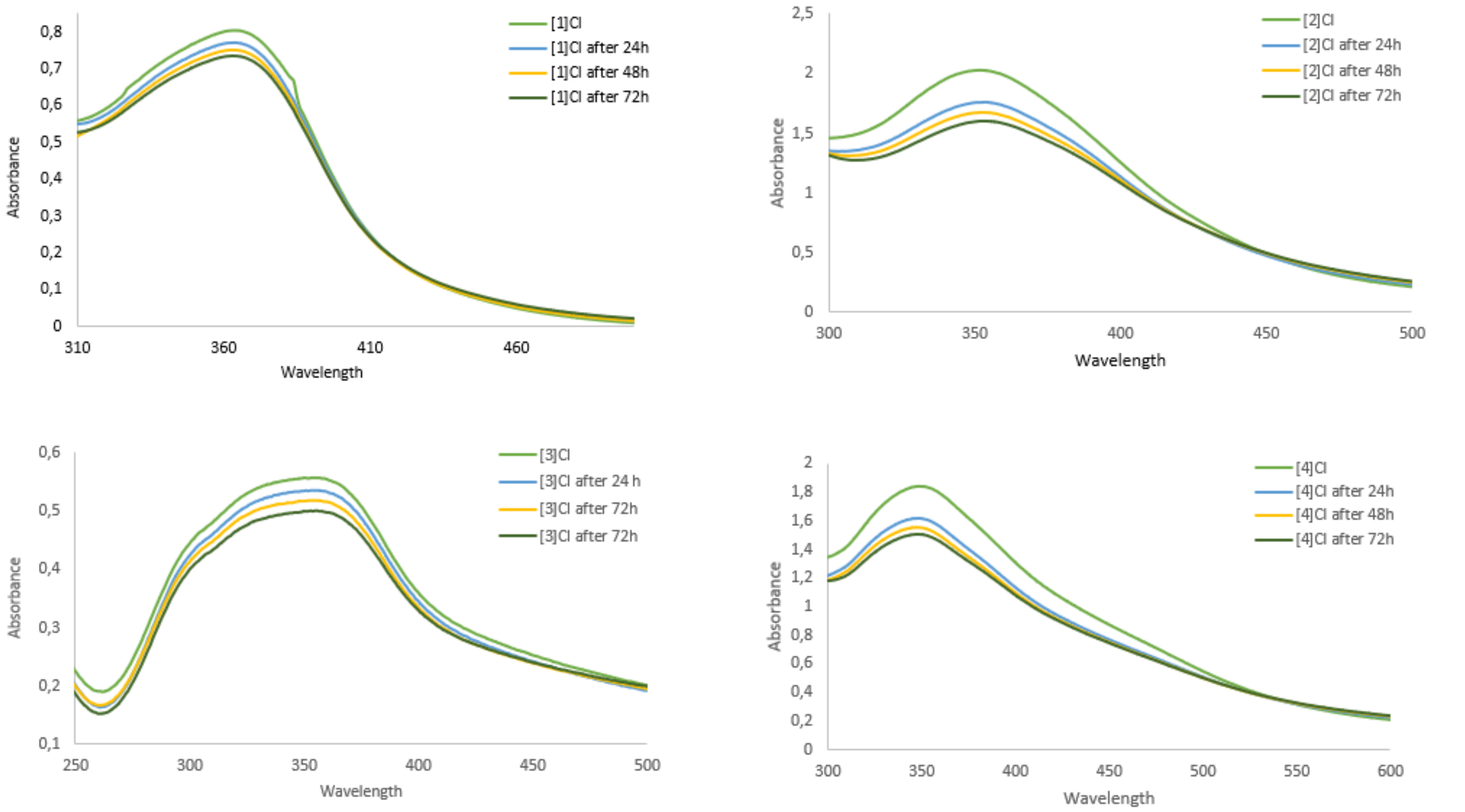

Figure S33. UV-Visible spectra for the stability studies of **[1]Cl-[4]Cl** under physiological conditions.

Molecular docking

The predictive models for DNA complexes with HZPcurc, HZPbdcurc, and their Os and Ru derivatives were generated by separately docking each compound onto the 3'-CCACCCACTACCCTGGTTGGATGCTAATGT-5' double-stranded DNA oligonucleotide. The DNA target was constructed and energy-minimized using Avogadro software,[1] while the 3D structures of the compounds were obtained from their crystallographic .cif files. Hydrogen atoms were added to the protein before analysis. Docking simulations were carried out with Autodock 4.2.6, which uses a Lamarckian genetic algorithm to investigate ligand binding within a pocket (Morris et al., 2009). The docking grid covered both the minor and major grooves of the DNA, with a grid spacing of 0.375 Å, an rms tolerance of 0.8 Å, and a maximum of 2,500,000 energy evaluations; all other parameters were left at their default settings.[2]

(1) Hanwell, M. D.; Curtis, D. E.; Lonie, D. C.; Vandermeersch, T.; Zurek, E.; Hutchison, G. R. Avogadro: An Advanced Semantic Chemical Editor, Visualization, and Analysis Platform. *J. Cheminform.* **2012**, *4*, 1–17.

(2) Mozzicafreddo, M.; Cuccioloni, M.; Cecarini, V.; Eleuteri, A. M.; Angeletti, M. Homology Modeling and Docking Analysis of the Interaction between Polyphenols and Mammalian 20S Proteasomes. *J. Chem. Inf. Model.* **2009**, *49* (2), 401–409.

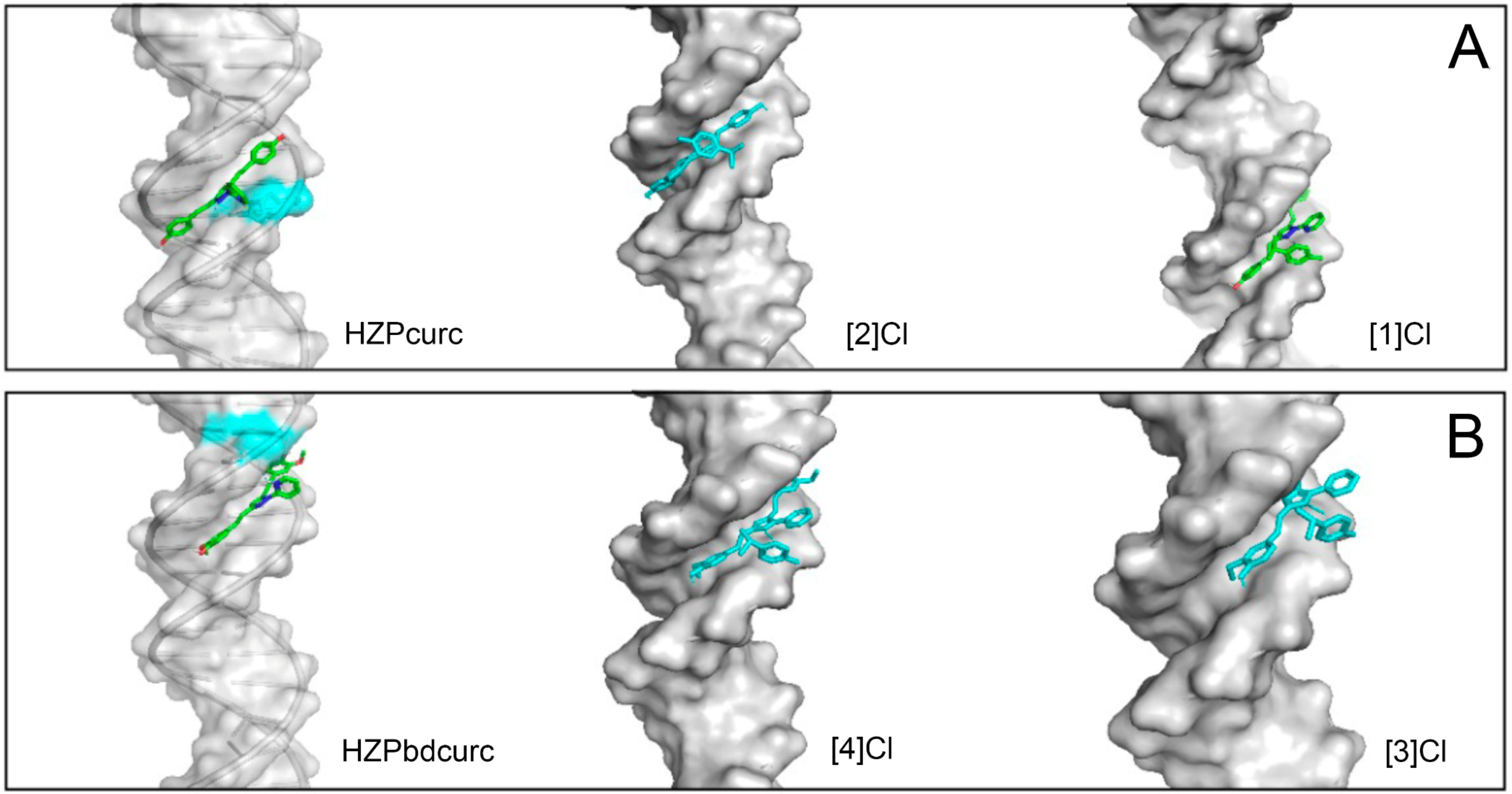

Figure S34. Visualization of the best scoring complexes formed upon docking the HZPcurc and HZPbdcurc ligands and the corresponding Ru and Os complexes on a dsDNA (solid grey surface). The nucleotides of the minor groove involved in the formation of H-bonds with the molecules of interest are coloured in light blue. Images were rendered with PyMOL 3.0.3.

Table S5: Computed ΔG values, number and types of interaction for the complexes with dsDNA obtained by molecular docking.

Molecule	ΔG (kcal/mol)	Number of Ionic interaction	Number of H-bond	Number of Weak H-bond
**HZPcurc**	-9.5	1	1	5
**HZPbdcurc**	-9.5	1	2	2
**[2]Cl**	-9.4	4		1
**[4]Cl**	-9.3	4		2
**[1]Cl**	-9.4	4		1
**[3]Cl**	-9.4	4		2

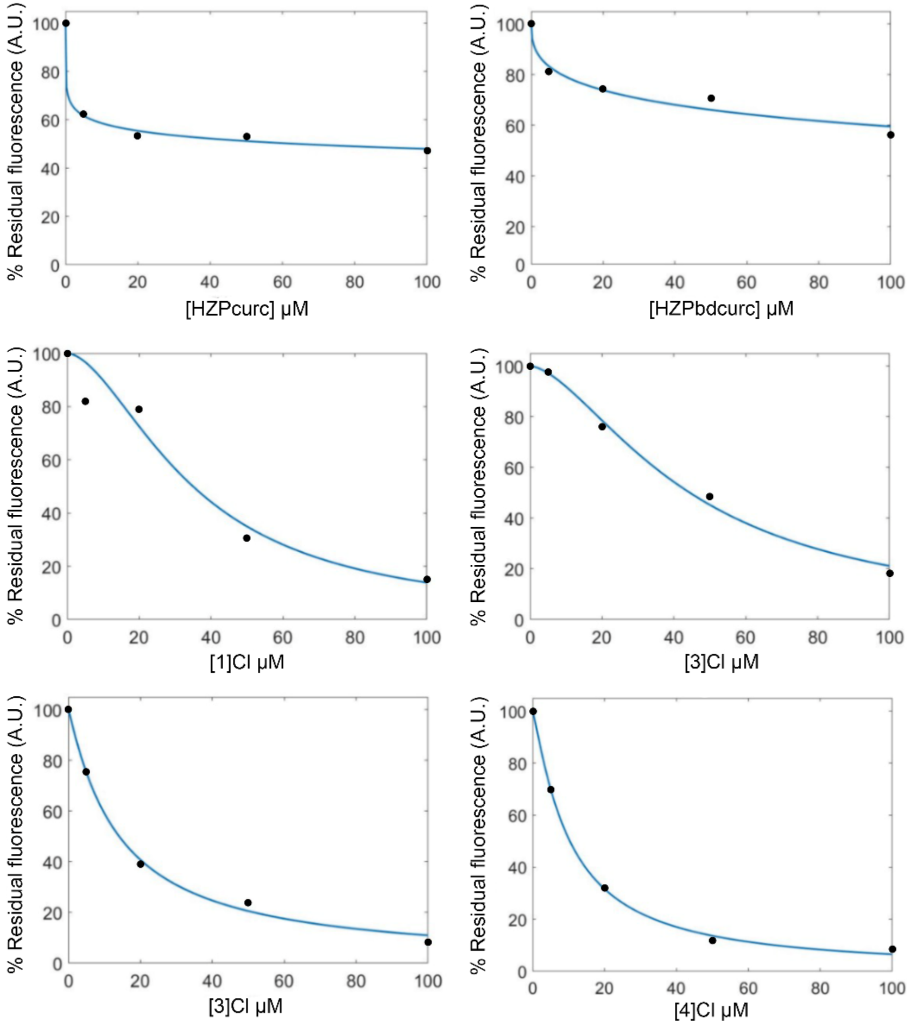

Figure S35. Decrease in the fluorescence emission at 500 nm of the complex between DNA-and GelRed (a minor groove binder) in the presence of increasing concentrations of the HZPcurc and HZPbdcurc ligands and corresponding Os and Ru complexes.
